# Atomic design of dual-metal hetero-single-atoms for high-efficiency synthesis of natural flavones

**DOI:** 10.1038/s41467-022-35598-3

**Published:** 2022-12-22

**Authors:** Xin Zhao, Ruiqi Fang, Fengliang Wang, Xiangpeng Kong, Yingwei Li

**Affiliations:** 1 State Key Laboratory of Pulp and Paper Engineering, School of Chemistry and Chemical Engineering, South China University of Technology, Guangzhou, 510640 China; 2 The School of Materials Science and Engineering, Harbin Institute of Technology, Shenzhen, 518055 China; 3 South China University of Technology–Zhuhai Institute of Modern Industrial Innovation, Zhuhai, 519175 China

**Keywords:** Heterogeneous catalysis, Metal-organic frameworks, Synthetic chemistry methodology

## Abstract

Single-atom (SA) catalysts provide extensive possibilities in pursuing fantastic catalytic performances, while their preparation still suffers from metal aggregation and pore collapsing during pyrolysis. Here we report a versatile medium-induced infiltration deposition strategy for the fabrication of SAs and hetero-SAs (M_a_N_4_/M_b_N_4_@NC; M_a_ = Cu, Co, Ni, Mn, M_b_ = Co, Cu, Fe, NC = N-doped carbon). In-situ and control experiments reveal that the catalyst fabrication relies on the “step-by-step” evolution of M_a_-containing metal-organic framework (MOF) template and M_b_-based metal precursor, during which molten salt acts as both pore generator in the MOF transformation, and carrier for the oriented infiltration and deposition of the latter to eventually yield metal SAs embedded on hierarchically porous support. The as-prepared hetero-SAs show excellent catalytic performances in the general synthesis of 33 kinds of natural flavones. The highly efficient synthesis is further strengthened by the reliable durability of the catalyst loaded in a flow reactor. Systematic characterizations and mechanism studies suggest that the superior catalytic performances of CuN_4_/CoN_4_@NC are attributed to the facilitated O_2_ activating-splitting process and significantly reduced reaction energy barriers over CoN_4_ due to the synergetic interactions of the adjacent CuN_4_.

## Abstract (web summary)

The preparation of single-atom catalysts still suffers from metal aggregation and pore collapsing during pyrolysis. Here the authors report a versatile medium-induced infiltration deposition strategy for the fabrication of a series of single-atom and hetero-single-atom catalysts.

## Introduction

Metal catalysts are widely used in modern chemical industry. Pyrolysis is the most commonly employed protocol to prepare heterogeneous metal-based catalysts with sizes from atom-scale (including single-atom (SA) and subnanocluster) to nanoscale and beyond^1–5^. Nevertheless, structural and compositional evolutions of the pyrolysis precursors at high temperatures are considerably intricate, leading to high difficulty in the controllable construction of uniform sites especially at atomic level. For the metal species, the high surface free energies usually lead to unwilled aggregations (i.e., Ostwald ripening)^6–8^. To improve the dispersion, strategies like reducing the content, partial evaporation or changing the topology of metal precursors have been developed, but the formation of metal-metal bonds is still not fundamentally avoided. In terms of the non-metal species (e.g., carbons and heteroatoms), which are generally transformed into supports or coordinated-atoms after pyrolysis, circumstances are even complex. At high temperatures, the inconsistent physicochemical properties (e.g., chemical environment, thermal stability and spatial distribution) of these non-metal species usually result in uncontrollable decomposition of the precursors^9–12^. Besides, the carbonization and subsequent atom migration processes are also highly random, and the evaporation and removal of non-metal components could possibly cause pore generation/collapsing and even dimension changes (e.g., from 3D to 2D)^13,14^. All these factors make the materials synthesis highly unpredictable. So far, it is still highly demanded to develop versatile and controllable pyrolysis synthesis routes to fundamentally understand the preparation of metal-based catalysts.

In this work, we demonstrate an effective and versatile medium-induced infiltration deposition strategy, which can simultaneously realize the monodispersion of metal species and controllable transformation of non-metal species for the synthesis of a series of SAs or hetero-SAs embedded on N-doped carbons (M_a_N_4_/M_b_N_4_@NC, M_a_ = Cu, Co, Ni, Mn; M_b_ = Co, Cu, Fe). Well-defined M_a_-containing metal-organic frameworks (MOFs) and M_b_-based phthalocyanine (Ph) are employed as pyrolysis precursor with a mixed molten salt (KCl-KBr) as medium. During pyrolysis, in-situ and control experiments directly reveal the morphology evolution of the template inside the medium. Systematical characterizations of M_a_N_4_/M_b_N_4_@NC indicate the homogeneous distribution of both M_a_N_4_ and M_b_N_4_ sites throughout the carbons with inter-connecting hierarchical porosity. Specifically, CuN_4_/CoN_4_@NC shows attractive catalytic performances in the domino synthesis of a wide range of flavones in both batch and flow reactor. A combination of experimental and density functional theory (DFT) results demonstrate that the high reactivity of CuN_4_/CoN_4_@NC is attributable to the enhanced O_2_ activation capability, and reduced reaction energy barriers over CoN_4_ arising from the synergetic interactions of CuN_4_. Moreover, the inter-connecting hierarchical pores can largely facilitate the mass transfer and promote the accessibility of hetero-SA sites.

## Results

### Synthesis and characterization of CuN_4_/CoN_4_@NC

**Fig. 1:**
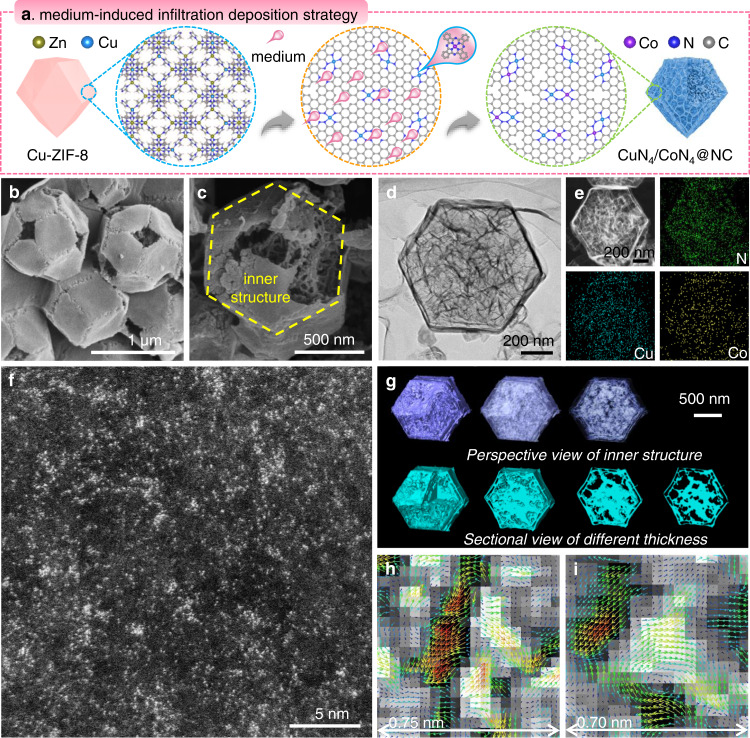
Synthesis and structural characterizations of CuN_4_/CoN_4_@NC. **a** Schematic illustration of the synthesis strategy. **b**, **c** SEM, **d** TEM, **e** HAADF-STEM and corresponding elemental mapping, and **f** AC HAADF-STEM image. **g** 3D reconstruction of a CuN_4_/CoN_4_@NC particle by HRTEM electron tomography. **h**, **i** DPC-STEM images.

The proposed medium-induced infiltration deposition strategy is of considerable flexibility and generality for the fabrication of various dual-metal hetero-SAs (Supplementary Figs. 1–3, Supplementary Tables 1–2) and single metallic SAs (Supplementary Figs. 4, 5, 12f, 13f, and Supplementary Tables 3–4). Preliminary analysis implies the Cu and Co SA sites are active in O_2_ activation and domino reaction (including condensation, cyclization and oxidative dehydrogenation), respectively. Therefore, the preparation of CuN_4_/CoN_4_@NC (schematically illustrated in Fig. 1a) is selected as a proof of concept to clarify some key details. Initially, uniform Cu-modified bimetallic zeolitic imidazolate framework (Cu-ZIF-8) crystals were assembled (Supplementary Figs. 6a, 7b)^15^. Next, Cu-ZIF-8 was mixed with a medium (i.e., a mixture of KCl and KBr salts) and Co-Ph. The obtained mixture was subjected to pyrolysis under Ar at 750 °C, which is higher than the decomposition temperatures of both Cu-ZIF-8 and Co-Ph (as determined by thermogravimetric analysis, Supplementary Fig. 6b, c). At high temperatures, Zn species are partially removed, and the salt medium melts and carries the Co-Ph to infiltrate into the Cu-ZIF-8 interior, during which the decomposition of Cu-ZIF-8 is accelerated with the formation of CuN_4_ and CoN_4_ sites embedded on the N-doped carbon support (CuN_4_/CoN_4_@NC). After pyrolysis, the KCl-KBr gradually seeds out, leaving the hierarchical interior of the as-prepared materials with micro- and meso- as well as macro-pores. The catalyst yield is about 42% (Supplementary Table 5).

Scanning electron microscopy (SEM) images (Fig. 1b) of CuN_4_/CoN_4_@NC show a rhombic dodecahedral morphology with a size of ca. 1 µm, and the high porosity is clearly observed from one broken particle (Fig. 1c). The porous interior and thin walls (ca. 10 nm in thickness) is further verified by the transmission electron microscopy (TEM) photographs (Fig. 1d and Supplementary Fig. 8). The low-dose high-resolution TEM (HRTEM) tomography is additionally conducted upon an individual particle to thoroughly study the topological morphology of CuN_4_/CoN_4_@NC (Fig. 1g, Supplementary Movies 1 and 2). In the perspective view, the shell of dodecahedron is tuned partially transparent to show the topology. As expected, the porous interior with inter-connecting pores is clearly observed. Besides, in the sectional views, TEM photographs of the particle with different thickness also demonstrate the homogeneous distribution of hierarchical pores. N_2_ isotherms and pore size distributions (Supplementary Fig. 9a, b, and Supplementary Table 2) further verify the hierarchical pore structure inside CuN_4_/CoN_4_@NC with a high Brunauer-Emmett-Teller (BET) specific surface area of 1112.2 m^2^ g^−1^.

High-angle annular dark-field scanning transmission electron microscopy (HAADF-STEM) and corresponding elemental mappings (Fig. 1e) indicate the homogeneous distribution of Cu and Co species. The content of Cu and Co in CuN_4_/CoN_4_@NC is 1.46 and 1.42 wt% (Supplementary Table 2), respectively. Spherical aberration-corrected HAADF-STEM (AC HAADF-STEM) image (Fig. 1f) demonstrates the isolated metal single-atoms including Cu and Co (bright dots), as different metals could not be clearly identified through limited Z-contrast differences. Supplementary Fig. 10 The homogeneous distribution of uniform Cu and Co atomic sites contributes to the formation of non-spherical Coulomb field in their adjacent regions (displayed by arrows in Fig. 1h, i, and Supplementary Fig. 11)^16^. The resultant electron transfer tendency in selected areas is clearly revealed via differential phase contrast (DPC) STEM. Under the influence of as-formed Coulomb field, carbonous support tends to donate electrons towards the immobilized metal SA sites.

Powder X-ray diffraction (XRD) patterns (Supplementary Fig. 9c) of CuN_4_/CoN_4_@NC display a broad peak centered at 26.2°, suggesting the MOF ligands have been transformed into graphitic carbon species^17^. The presence of defects is verified by Raman analysis (Supplementary Fig. 9d), showing an *I*_D_/*I*_G_ value of ca. 1.12. Notably, no obvious characteristic diffraction of Cu or Co species is detected (Supplementary Fig. 9c), possibly due to their atomic dispersions. In CuN_4_/CoN_4_@NC, the N content is relatively high (13.4 wt%, Supplementary Table 2) with the dominance of pyridinic-N and graphitic-N species (Supplementary Fig. 9e). The C *1s* X-ray photoelectron spectroscopy (XPS, Supplementary Fig. 9f) suggests the significant fraction of C–N species arising from the high N content^18^. Moreover, the Zn *2p* XPS analysis (Supplementary Fig. 12) indicates negligible Zn residues in the as-synthesized CuN_4_/CoN_4_@NC.

### Atomic structure analysis of CuN_4_/CoN_4_@NC

**Fig. 2:**
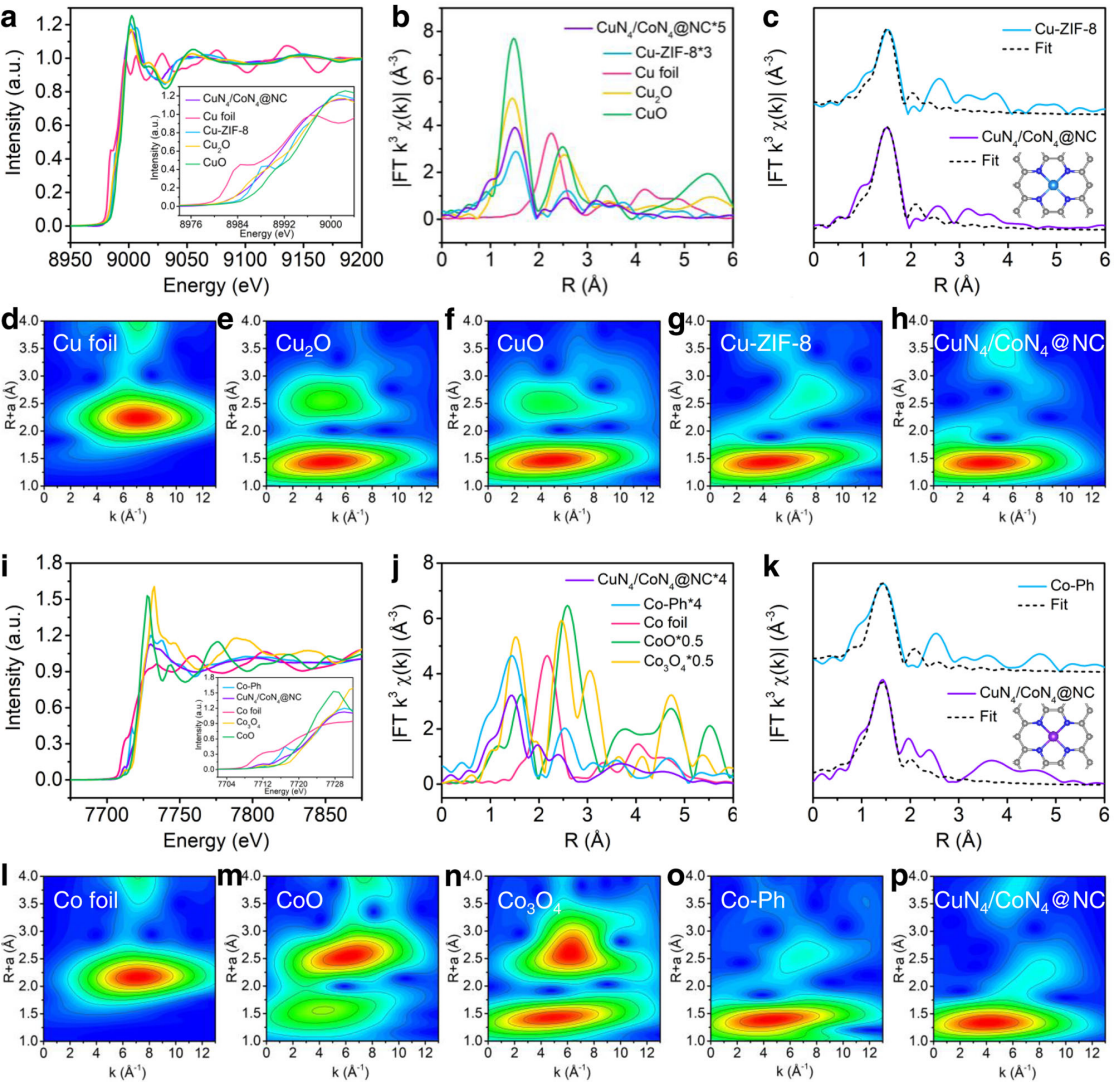
Atomic structural analysis of CuN_4_/CoN_4_@NC. **a** Cu K-edge XANES spectra, **b** Fourier-transformed (FT) k^3^-weighted EXAFS spectra for the Cu K-edge, **c** Cu K-edge EXAFS fitting curves of CuN_4_/CoN_4_@NC and Cu-ZIF-8 in R space (inset: model of CuN_4_ sites), **d**–**h** Wavelet-transformed k^3^-weighted EXAFS spectra. **i** Co K-edge XANES spectra, **j** FT k^3^-weighted EXAFS spectra for the Co K-edge, **k** Co K-edge EXAFS fitting curves of CuN_4_/CoN_4_@NC and Co-Ph in R space (inset: model of CoN_4_ sites), **l**–**p** Wavelet-transformed k^3^-weighted EXAFS spectra.

X-ray absorption fine structure (XAFS) spectra were recorded to elucidate the electronic structures of Cu and Co species in CuN_4_/CoN_4_@NC at atomic level (Fig. 2, Supplementary Figs. 13, 14, and Supplementary Table 6). A few Cu- or Co-based standard samples (e.g., Cu foil, Cu_2_O, CuO, Co foil, CoO, and Co_3_O_4_) and composites (Cu-ZIF-8 and Co-Ph) were utilized as references. Cu K-edge X-ray absorption near-edge structure (XANES) spectrum (Fig. 2a) of CuN_4_/CoN_4_@NC situates between that of Cu^0^ and Cu^2+^, suggesting a valence state of 0 to +2 for the Cu species. The high similarity of CuN_4_/CoN_4_@NC and Cu-ZIF-8 spectra implies similar coordination configurations of their Cu moieties. The Fourier-transformed (FT) k^3^-weighted extended X-ray absorption fine structure (EXAFS) spectrum (Fig. 2b) of Cu in CuN_4_/CoN_4_@NC exhibits a significant peak at 1.5 Å (like Cu-ZIF-8), which can be attributed to the Cu-N coordination. Notably, no Cu–Cu coordination peaks at around 2.24 Å are detected, confirming the single-atom distribution (rather than aggregation) of Cu in CuN_4_/CoN_4_@NC. The EXAFS fitting curves (Fig. 2c) recover a N_4_ coordination configuration of Cu with a Cu–N bond length of 1.96 Å (Supplementary Table 6). The maximum at 4.0 Å^–1^ in wavelet transform (WT)-EXAFS of CuN_4_/CoN_4_@NC (Fig. 2h) is assigned to Cu–N bonding, in good accordance with Cu-ZIF-8.

Similarly, the Co K-edge XANES spectra (Fig. 2i) of CuN_4_/CoN_4_@NC display a near-edge absorption between Co foil and CoO, suggesting a valence state of lower than +2. The similar peak location of CuN_4_/CoN_4_@NC and Co-Ph indicates analogous coordination configurations of their Co species. The increased peak intensity of CuN_4_/CoN_4_@NC is ascribed to the distorted D_*4h*_ symmetry possibly resulting from the Cu–N_4_ sites^19,20^. The FT k^3^-weighted Co EXAFS spectrum (Fig. 2j) of CuN_4_/CoN_4_@NC shows one remarkable peak at 1.43 Å, very close to that of Co-Ph (1.44 Å). As no Co–Co coordination peaks (2.16 Å) are detected, we could infer the Co single-atoms are predominantly coordinating to N. Moreover, the Co K-edge EXAFS fitting curves (Fig. 2k) match well with the proposed structural models. The optimized fitting parameters (Supplementary Table 6) suggest each Co atom is coordinating to four N atoms with a bond length of ca. 1.98 Å, as is also verified by the highly similar WT-EXAFS spectra of CuN_4_/CoN_4_@NC and Co-Ph.

### Possible formation mechanism of CuN_4_/CoN_4_@NC

To interpret the structural evolution process and formation mechanism of CuN_4_/CoN_4_@NC, some control experiments were carried out (Supplementary Table 1). To disclose the role of salt medium, CoN_4_@NC and CuN_4_@NC were prepared via the similar procedures (Supplementary Figs. 4, 5, 13f, 14f, and Supplementary Tables 2–4). Specifically, pristine ZIF-8 was employed as precursor for the preparation of CoN_4_@NC. The obtained material features inter-connecting porosity and thin shells, throughout which atomic Co sites are clearly observed. The Co K-edge XANES and EXAFS spectra of CoN_4_@NC demonstrate the atomic affixing of Co on N-doped carbon via Co–N_4_ coordination. CoN_4_@NC possesses a relatively higher BET specific surface area (1598.7 m^2^ g^–1^) with more micro- and mesopores than CuN_4_/CoN_4_@NC. The successful preparation of CoN_4_@NC implies the carrier role of salt medium in the infiltration of Co-Ph through the pores/channels of ZIF-8. To further elucidate this, CuN_4_@NC was also fabricated in the absence of Co-Ph (Supplementary Fig. 4 and Supplementary Tables 2–5). The enhanced specific surface area (2070.1 m^2^ g^–1^) is possibly ascribing to the absence of Co-Ph (and derivatives) which might block the pores.

However, in the absence of salt medium, significant metallic aggregations are observed over the as-obtained Cu_x_/Co_x_@NC-750 (Supplementary Fig. 15 and Supplementary Table 2). The aggregated Co species around rhombic dodecahedra are derived from Co-Ph, which could not migrate into the interior of Cu-ZIF-8 without a salt medium. The small specific surface area (144.0 m^2^ g^–1^) and reduced micropore fraction is possibly related to the sluggish generation and coalescence process of pores. These results demonstrate the essential roles of salt medium in Co-Ph diffusion and hierarchical pore generation. The successful preparation of CoN_4_@NC and CuN_4_@NC suggests the high potential of the present strategy in fabricating single metallic SAs through simply modifying the MOF or metal precursor.

On the basis of the above results, a plausible mechanism is proposed for the synthesis of CuN_4_/CoN_4_@NC via the developed medium-induced infiltration deposition strategy. At the beginning of the heating process, KCl-KBr plays the role of carrier, which melts and carries the Co-Ph precursor to infiltrate into the pores/channels of Cu-ZIF-8 by capillarity (as disclosed by in-situ TEM experiment, Supplementary Fig. 16 and Supplementary Movie 3). When the temperature reaches 600 °C, the decomposition of Cu-ZIF-8 begins (Supplementary Fig. 6b), during which the KCl-KBr medium acts as both carbonization booster and metal stabilizer. As a result, the graphitization of organic ligands is remarkably accelerated (Supplementary Figs. 9d and 15n), and the agglomeration of metal nodes is suppressed due to the formation of M-X complexes (M = Zn, Cu and Co; X = Cl and Br). Notably, the presence of KCl-KBr leads to a time difference between the decomposition of Cu-ZIF-8 and Co-Ph, which is crucial to avoid the formation of metal-metal bonds during the “step-by-step” evolution from Cu-ZIF-8 and Co-Ph to CuN_4_/CoN_4_@NC. On the other hand, the molten salt could also facilitate the generation of defects, which gradually migrate and coalesce into micro-, meso- and even macropores at high temperature. In the cooling process, KCl-KBr gradually seeds out and helps retaining the as-formed inter-connecting channels within the carbon support (Supplementary Movies 1 and 2). Eventually, the CuN_4_ and CoN_4_ sites are affixed on the N-doped carbons with hierarchical porosity.

**Fig. 3:**
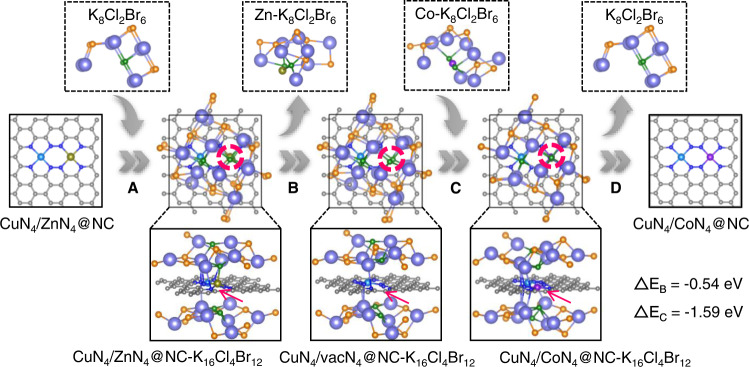
Mechanism of the synthesis strategy. Proposed mechanism for the formation of CuN_4_/CoN_4_@NC.

DFT calculations (Fig. 3, Supplementary Fig. 17, and Supplementary Table 7) are additionally performed to verify the key issues of the proposed formation mechanism of CuN_4_/CoN_4_@NC. The structural model of pristine Cu-ZIF-8 is simplified as CuN_4_/ZnN_4_@NC, and the KCl-KBr medium in molten state is established as K_8_Cl_2_Br_6_ (or K_16_Cl_4_Br_12_). In CuN_4_/ZnN_4_@NC, the positively charged Cu and Zn atoms tend to coordinate with Cl^−^ and Br^−^ anions. The adsorption energy of Cu atom to Cl^−^ and Br^−^ is −0.63 eV (Supplementary Fig. 17), higher than that of Zn atom (−1.25 or −1.20 eV, respectively). In this concern, it is relatively easier for Zn, in comparison with Cu, to bond with Cl^−^ and Br^−^. Due to the lower energy, Zn is more inclined to combine with Cl^−^ to form CuN_4_/ZnN_4_@NC-K_16_Cl_4_Br_12_ (Fig. 3, step A), as indicated by red circle and arrow. Subsequently, Zn species are removed from CuN_4_/ZnN_4_@NC by molten salt in the form of Zn-K_8_Cl_2_Br_6_ (Fig. 3, step B), and the energy change of this process is −0.54 eV. Next, the Co-K_8_Cl_2_Br_6_ (generated in a similar way to Zn-K_8_Cl_2_Br_6_) locates onto the as-formed vacancy (vac) in the residue CuN_4_/vacN_4_@NC-K_16_Cl_4_Br_12_ with Co atom coordinating to four N atoms to form CuN_4_/CoN_4_@NC-K_16_Cl_4_Br_12_ (Fig. 3, step C), and the energy change of this process is −1.59 eV. Finally, CuN_4_/CoN_4_@NC is obtained after the removal of K_8_Cl_2_Br_6_ by thorough washing.

Some control experiments were then conducted to uncover the key details in the formation of CuN_4_/CoN_4_@NC. The molten salt could facilitate the removal of Zn (rather than Cu) through coordination. To elucidate this, eight possible circumstances (including single/double metallic counterparts with and without K_8_Cl_2_Br_6_ in the system, Supplementary Fig. 18) are simulated, and results indicate the presence of K_8_Cl_2_Br_6_ significantly reduce the energy barrier in the removal of metal species in MOF (a, c, e, g *vs*. b, d, f, h). According to the results, the removal of Zn from MOF to form CuN_4_/vacN_4_@NC-K_16_Cl_4_Br_12_ is most favourable because of its lower energy barrier (−0.54 vs 0.12 eV). Specifically, the remarkable decomposition energy differences (Supplementary Fig. 19) between Zn atom (−0.54 eV) and ZnN_4_ (20.99 eV) suggest the Zn species are removed dominantly in atomic state.

### Electronic property of CuN_4_/CoN_4_@NC

Considering the possible configurations of CuN_4_ and CoN_4_ sites in CuN_4_/CoN_4_@NC, three potential model structures (CuN_4_/CoN_4_@NC, CuN_4_/CoN_4_@NC(*2N*), CuN_4_/CoN_4_@NC(*0N*)) were established (Supplementary Fig. 20). The molecular dynamics simulations were conducted at 450 K for 20 ps upon these models to evaluate the thermodynamic stability (Supplementary Fig. 21). The fluctuation of total energy is clearly observed oscillating around the equilibrium state without structural disruption, verifying the reliable thermodynamic stability of these models. Considering the homogeneous distribution throughout the composite, the CuN_4_/CoN_4_@NC model (Supplementary Fig. 20c) is selected for subsequent investigations. The charge distributions of CuN_4_@NC, CoN_4_@NC, and CuN_4_/CoN_4_@NC are simulated (Supplementary Fig. 22) along with the corresponding Bader charges calculated (Supplementary Table 8). These results imply the synergistic interactions between CuN_4_ and CoN_4_ in CuN_4_/CoN_4_@NC lead to remarkable electron accumulations and shifting in the density of states (DOS, Supplementary Fig. 23) of Co and Cu.

### Catalytic performances in the synthesis of flavone

Flavone (also known as 2‐phenylchromone) and derivatives are a class of natural compounds possessing fantastic biological activities like anticancer, antitumor, antibacterial, antimicrobial, antioxidant, estrogenic, and antiestrogenic properties, therefore are widely utilized in drug-relevant and biomedical fields^21–24^. Accordingly, tremendous efforts have been devoted to synthesizing such β-*O*-substituted unsaturated ketones. Homogeneous routes like Baker-Venkataraman rearrangement, Kostanecki-Robinson reaction, Allan-Robinson, and Claisen condensation attained significant advances while suffering from harsh reaction conditions and the use of corrosive additives (e.g., strong acids or bases) or toxic oxidants (e.g., iodine-containing compounds, chromate, permanganate, pypocholoride, and 2,3-dichloro-5,6-dicyano-1,4-benzoquinone (DDQ)). In heterogeneous systems, palladium-based catalysts are demonstrated effective, but it is difficult to directly synthesize flavones with various substituted functional groups (to adjust the corresponding medicinal effects). In view of synthetic versatility, atom economy and environmental benign, it is highly desired to develop heterogeneous catalytic systems for highly efficient preparation of flavones.

**Fig. 4:**
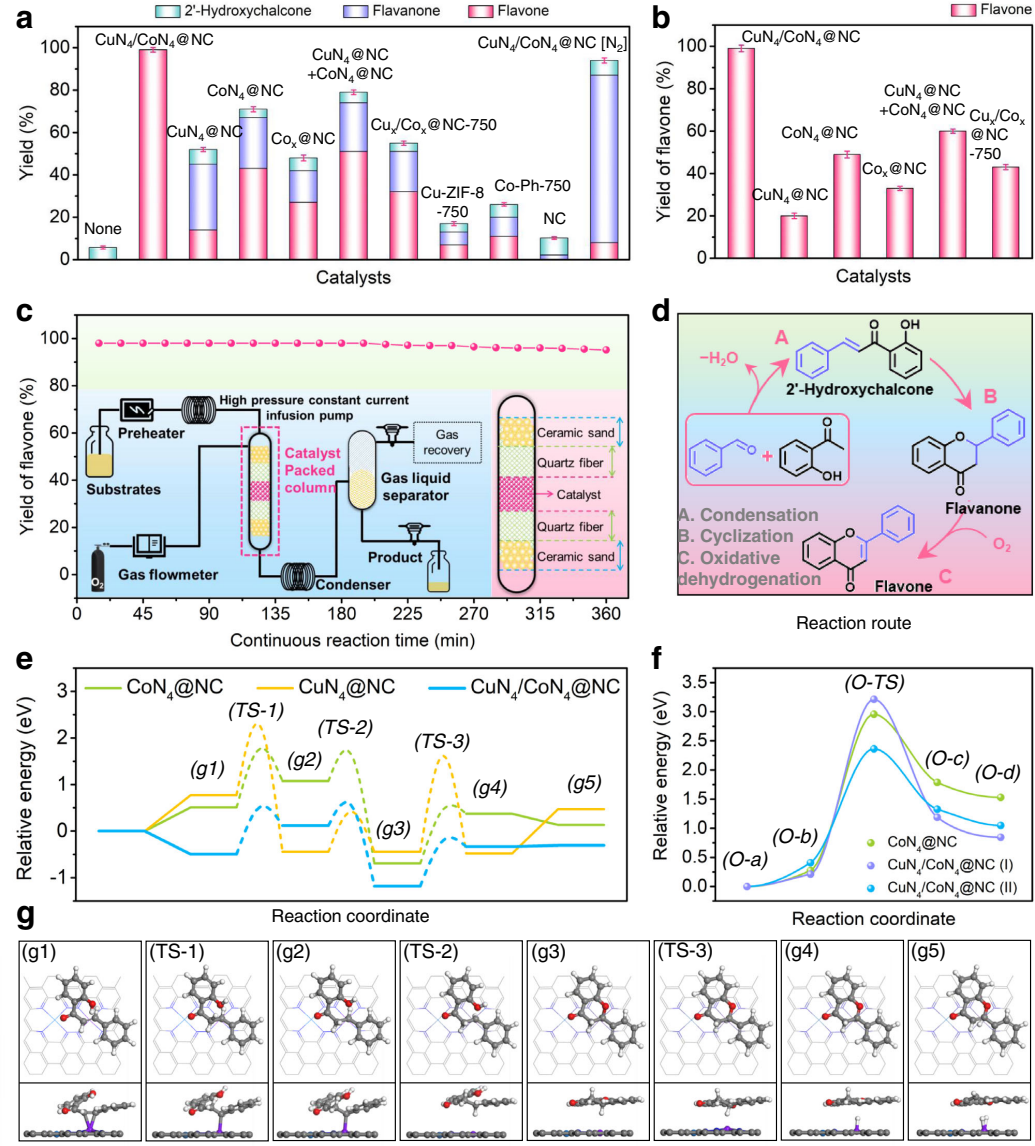
Catalytic performance of CuN_4_/CoN_4_@NC and reaction mechanistic studies. **a** Domino reaction of benzaldehyde and 2'-hydroxyacetophenone to flavone over different catalysts. **b** Oxidative dehydrogenation of flavanone to flavone over different catalysts. **c** Reaction results obtained in the continuous flow reactor (inset: simplified diagram of the reactor (left) and detailed description of catalyst packed column (right)). **d** Proposed reaction routes. **e** Free energy diagram and **g** atomistic structures of the initial state, transition state, and final state for the reaction pathways from 2'-hydroxychalcone to flavone on CuN_4_/CoN_4_@NC. The grey, blue, sky blue, purple, red, and white balls represent C, N, Cu, Co, O, and H atoms, respectively. “TS” denotes a transition state. **f** CI-NEB results of O_2_ disassociation. The error bars represent standard deviation based on three measurements.

Here, a domino catalytic system is developed for the general synthesis of flavones, as exemplified by the conversion of benzaldehyde and 2'-hydroxyacetophenone into flavone (Supplementary Figs. 24–26) over CuN_4_/CoN_4_@NC. The domino transformation consists of aldol condensation, intramolecular cyclization and oxidative dehydrogenation (ODH) with 2'-hydroxychalcone and flavanone as the intermediates (Fig. 4a, d). The benzaldehyde conversion, flavone selectivity and yield are calculated according to Eqs. (1–3). Typically, the reaction was carried out at 140 °C under 2 bar O_2_. No flavone product is detected in the blank run without catalyst (Supplementary Table 9, entry 1). The negligible flavone yield over NC obtained via the pyrolysis of ZIF-8 (Supplementary Fig. 27) excludes the contribution of possible Zn residue (Supplementary Table 9, entry 2). To our delight, CuN_4_/CoN_4_@NC is highly active and selective in this reaction, giving a complete benzaldehyde conversion and 99% flavone yield (Supplementary Table 9, entry 3). In contrast, Cu- and Co-based counterparts including CuN_4_@NC, CoN_4_@NC, Co_x_@NC, Cu_x_/Co_x_@NC-750, Cu-ZIF-8-750, Co-Ph-750, Co-ZIF-8-750, 600-Cu/Co@NC and 900-Cu/Co@NC, (Supplementary Figs. 4, 5, 14, 28–33, and Supplementary Tables 1, 2) all show unsatisfied catalytic performances (Supplementary Table 9, entries 4–12). In particular, only 55% benzaldehyde conversion and 32% flavone yield are obtained over Cu_x_/Co_x_@NC-750 (Supplementary Table 9, entry 7), indicating the key role of well-defined atomic sites in the catalytic reaction. Similar tendency is also observed for Cu-ZIF-8-750 or Co-Ph-750, on which the flavone yields are much lower as compared with CuN_4_@NC or CoN_4_@NC, respectively. The moderate activity of a physical mixture of CuN_4_@NC and CoN_4_@NC (Supplementary Table 9, entry 8) suggests the essential role of synergistic interactions between the adjacent CuN_4_ and CoN_4_ sites in pursuing high flavone yields. The unsatisfied catalytic performances of 600-Cu/Co@NC and 900-Cu/Co@NC indicate the critical impact of pyrolysis temperature. The reaction under N_2_ atmosphere gives negligible flavone while significant amount of flavanone, implying the transformation of the flavanone intermediate requires aerobic condition (Supplementary Table 9, entry 13). As expected, the reaction using flavanone as the substrate proceeds smoothly and affords similar flavone yield over CuN_4_/CoN_4_@NC (Fig. 4b and Supplementary Table 10).

### Continuous flow synthesis

To demonstrate the applicability of the catalytic system, CuN_4_/CoN_4_@NC was loaded into a continuous flow reactor (Fig. 4c). To our delight, nearly quantitative flavone yield is obtained within 360 min of reaction without any by-product detected, achieving a turnover number (TON) as high as 3842 (Eq. 4) and a space time yield (STY) of 66.7 g·g_cat_^–1^·h^–1^ (Eq. 5). To the best of our knowledge, this work represents an example of heterogeneous domino catalysis synthesis of flavones in a continuous flow reactor, showing great potential for practical application.

### Reusability of CuN_4_/CoN_4_@NC

After reaction, CuN_4_/CoN_4_@NC was easily collected by centrifugation and directly reused for next runs. No obvious activity loss is observed after reused for at least six times (Supplementary Fig. 34a), indicating its good recyclability. Characterization results including AC HAADF-STEM and XANES (Supplementary Figs. 35, 36, Supplementary Table 11) confirm the unchanged structural and compositional properties of the used CuN_4_/CoN_4_@NC in comparison with the fresh one. In addition, the metal leaching examination (Supplementary Table 2) and hot filtration experiment (Supplementary Fig. 34b) results also demonstrate the reliable durability of CuN_4_/CoN_4_@NC and the heterogeneous nature of the developed domino reaction system. These results indicate the obtained hetero-SAs are stable under the investigated reaction conditions.

### Reaction mechanism

DFT calculations were conducted to gain theoretical insights into the reaction mechanism of the domino synthesis of flavone over CuN_4_/CoN_4_@NC. Considering the aldol condensation of benzaldehyde and 2'-hydroxyacetophenone to 2'-hydroxychalcone is a common and simple reaction, the mechanism investigation begins with 2'-hydroxychalcone transformation. The entire reaction process consists of eight elementary steps (Fig. 4e, g, and Supplementary Figs. 37–39). At first, one 2'-hydroxychalcone molecule is adsorbed onto the CoN_4_ site of CuN_4_/CoN_4_@NC (CoN_4_ for CoN_4_@NC and CuN_4_ for CuN_4_@NC) via the C=C group to form (g1). The adsorption energy over CuN_4_/CoN_4_@NC is negative (−0.50 eV), significantly lower than that of CuN_4_@NC (0.77 eV) and CoN_4_@NC (0.51 eV). The adsorbed 2'-hydroxychalcone molecule undergoes an intramolecular reaction through (TS-1) to form (g2), overcoming a relatively low energy barrier of 1.01 eV (1.25 eV for CoN_4_@NC and 1.51 eV for CuN_4_@NC). Afterwards, (g2) is easily transformed into (g3) (flavanone) via an intramolecular cyclization process through (TS-2), the energy barrier is as low as 0.43 eV (0.57 and 0.86 eV for CoN_4_@NC and CuN_4_@NC, respectively). Subsequently, (g3) is dehydrogenated into (g4) through (TS-3). This step is also facilitated over CuN_4_/CoN_4_@NC in comparison with CoN_4_@NC and CuN_4_@NC due to the reduced energy barrier (0.99 vs 1.16 and 2.06 eV). Finally, the generation of (g5) (*flavone) undergoes a dehydrogenation process, which is endothermic over CuN_4_/CoN_4_@NC (0.13 eV) and CuN_4_@NC (0.47 eV) while exothermic over CoN_4_@NC (−0.31 eV).

Moreover, the O_2_ activation mechanism was also investigated (Supplementary Table 11). Projected density-of-states (PDOS) are calculated to elucidate interactions between O_2_ molecular orbitals and *d* orbitals of the metal center in the catalysts (Supplementary Fig. 40). When O_2_ is adsorbed on CuN_4_, the O and Cu atom orbitals contribute to PDOS separately, indicating the physical adsorption of O_2_. DFT results reveal an O_2_ adsorption energy of −0.22 eV over CuN_4_@NC with a 0.125 nm O–O bond length. On the other hand, after O_2_ adsorption upon CoN_4_, characteristic peaks of O atom orbitals (−7.5 to −5 eV for π bond and −2 to 2 eV for π* bond) become broad and weak. Besides, the π* bond of O atom orbitals contributed by *p*_*z*_ and *p*_*x*_ orbitals of O atoms are overlapped by *d*_*z*_^*2*^ and *d*_*xz*_ orbital of Co center, respectively, and all these orbitals contribute to the PDOS. These results suggest the formation of Co–O bonds, in other words, O_2_ is chemically adsorbed on CoN_4_. The chemical adsorption of O_2_ over CoN_4_@NC and CuN_4_/CoN_4_@NC leads to reduced adsorption energy (−0.88 eV and −0.82 eV, respectively) and increased O–O bond length (0.129 nm). Moreover, O_2_ adsorption causes remarkable charge redistribution in O_2_ molecule and Co sites, and the O–O bond is weakened with the Co–O bonds newly formed (Supplementary Fig. 41). After O_2_ adsorption, the electron depletion around CoN_4_ is enhanced in CuN_4_/CoN_4_@NC relative to CoN_4_@NC, which is concerned beneficial for the subsequent O_2_ disassociation.

Climbing Image Nudged Elastic Band (CI-NEB) method was utilized to explore the transition state and activation energy during O_2_ dissociation (Fig. 4f, Supplementary Fig. 42 and Supplementary Table 12). For CoN_4_@NC, the adsorbed O_2_ is dissociated into two O atoms overcoming the activation energy barrier of 2.96 eV. One of the resultant O atoms locates at the top of CoN_4_ and the other moves to the bridge site of the closest C–C bond, and the dissociation energy was 1.53 eV. In terms of CuN_4_/CoN_4_@NC, two potential configurations (I and II) are proposed requiring different activation energy barriers (3.21 eV and 2.36 eV, respectively), i.e., one O atom is stabilized on CoN_4_ and the other may be captured by the second closest C–C bond (configuration I) or the closest carbon (configuration II), with a dissociation energy of 0.85 eV and 1.05 eV, respectively. Hence, the O_2_ dissociation is much easier to take place over CuN_4_/CoN_4_@NC through configuration II due to its lower energy barrier. To identify the reactive oxygen species (ROS) generated during the reaction, electron paramagnetic resonance (EPR) experiments were conducted over the reaction solution fetched online with the addition of 5,5-dimethyl-1-pyrroline N-oxide (DMPO) (Supplementary Fig. 43). The remarkable sextet EPR signal of DMPO–OOH (a spin adduct derived from DMPO–•O_2_^-^) confirms the formation of •O_2_^–^ radicals^25^, indicating the disassociation of molecular O_2_ features •O_2_^–^ radicals as the intermediate.

Based on the above results and discussions, the domino reaction could be divided into two main steps, O_2_ activation and 2'-hydroxyacetophenone transformation. The two steps are almost equally important to attain the overall catalytic efficiency. The as-synthesized CuN_4_/CoN_4_@NC exhibits improved capability in reducing energy barriers for both steps as compared with the counterparts, thus achieving significantly enhanced catalytic performance in the entire domino reaction.

### Substrate scope

Substrate adaptability is a critical parameter to evaluate the versatility and efficiency of a catalytic system. In this work, various aromatic aldehydes and ketones with one to three substituted functional groups were employed as substrates to investigate the general applicability of CuN_4_/CoN_4_@NC. For the aldehydes with one substituted group (including electron-donating and electron-withdrawing ones), excellent yields (89–99%) of the corresponding flavones are obtained (Figs. 5, 1a–15a). With two substituted groups on the aromatic aldehydes, the products yields are 83–91% (Figs. 5, 16a–20a). On the other hand, the domino reactions between benzaldehyde and substituted 2'-hydroxyacetophenone also proceed smoothly (Figs. 5, 21b–29b), further demonstrating the substrate scope of flavones synthesis. Moreover, flavone derivatives with multiple substituents can also be obtained in moderate to good yields (70–78%) over CuN_4_/CoN_4_@NC (Figs. 5, 30c–32c).

**Fig. 5:**
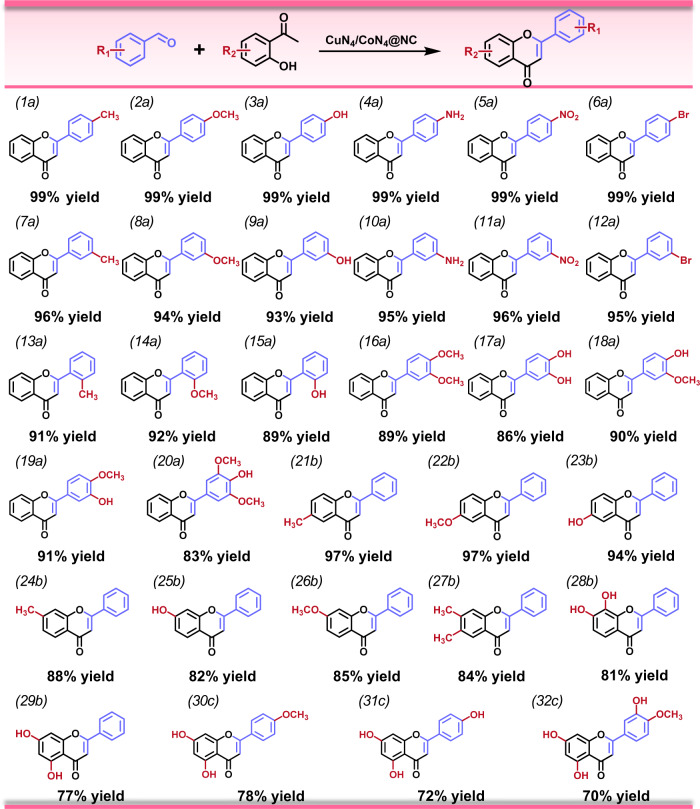
Scope of the domino reaction to various flavones. Reaction conditions: aldehyde (1 mmol), ketone (1.5 mmol), catalyst (total metal, 1.4 mol%), *n*-hexanol (4 mL), O_2_ (2 bar), 140 °C, 12 h. Conversion and yield were determined by GC-MS based on aldehydes.

The wide substrate scope of CuN_4_/CoN_4_@NC further demonstrates its high efficiency in synergistically catalyzing the domino reaction to produce substituted flavones with fantastic biological activities. For instance, chrysin (Figs. 5, 29b) features a wide range of pharmacological activities including anti-tumor, anti-allergic, anti-viral, and hypoglycemic effects. It is also confirmed effective in inhibiting HIV activation in latent infection model. Acacetin (Figs. 5, 30c) is naturally synthesized inodoratum and acacia, which shows effective anti-cancer activity via inducing cancer cell cycle arrest, apoptosis and autophagy. Apigenin (Figs. 5, 31c) is a natural antioxidant existing in chamomile tea, which is effective in lowering blood pressure, relaxing blood vessels, and inhibiting tumors. Diosmetin (Figs. 5, 32c) is a natural flavonoid in lemon possessing anti-oxidant, and anti-mutation biological activities, which can suppress the activation of carcinogens by inhibiting CYP1A1 enzyme. Notably, ^1^H-NMR and ^13^C-NMR spectra (Supplementary Figs. 44–51) are employed to identify these products.

## Discussion

In summary, a versatile medium-induced infiltration deposition strategy is developed for the fabrication of a variety of metal SAs or dual-metal hetero-SAs affixed on hierarchically porous N-doped carbons. The underlying mechanism of composition and structure evolution during the materials preparation uncovers the essential roles of molten salt medium as the carrier of precursor, pore generator and metal stabilizer. The high catalytic efficiencies of the as-obtained hetero-SA catalysts (especially CuN_4_/CoN_4_@NC) are demonstrated in the domino synthesis of flavone and 32 kinds of derivatives from substituted benzaldehydes and 2'-hydroxyacetophenones, achieving an STY of 66.7 g·g_cat_^–1^·h^–1^ for flavone in a flow reactor. Reaction mechanism studies suggest the attractive catalytic performance over CuN_4_/CoN_4_@NC is ascribed to the promoted O_2_ activation-dissociation capability and reduced energy barriers of critical elementary steps in the synthesis of flavones. This work might provide insights into the highly efficient synthesis of natural products like flavones as demonstrated here.

## Methods

### Chemicals

All chemicals were purchased from commercial sources and directly used without further purification. Zinc acetate dihydrate (Zn(OAc)_2_·2H_2_O, 99.99%, Aladdin), copper acetate monohydrate (Cu(OAc)_2_·H_2_O, 99%, Aladdin), cobalt acetate tetrahydrate (Co(OAc)_2_·4H_2_O, 99.99%, Aladdin), nickel acetate tetrahydrate (Ni(OAc)_2_·4H_2_O, 99%, Aladdin), manganese acetate tetrahydrate (Mn(OAc)_2_·4H_2_O), 2-methylimidazole (2-MI, 98%, Aladdin), potassium bromide (KBr, 99.5%, Aladdin), potassium chloride (KCl, 99.5%, Aladdin), cobalt phthalocyanine (Co-Ph, 95%, Aladdin), copper phthalocyanine (Cu-Ph, 90%, Aladdin), and iron phthalocyanine (Fe-Ph, 97%, Aladdin).

### Synthesis of ZIF-8, Co-ZIF-8, Cu-ZIF-8, Ni-ZIF-8, and Mn-ZIF-8

In a typical synthesis, 2-methylimidazole (2-MI, 0.068 mol) was dissolved in 25 mL deionized water and stirred for 5 min at room temperature. Afterwards, a 25 mL aqueous solution containing Zn(OAc)_2_·2H_2_O (0.0068 mol) was added to the above solution. After a 5-h aging process, the white powder was collected through centrifugation and washing with methanol for three times to obtain ZIF-8.

Cu-ZIF-8, Co-ZIF-8, Ni-ZIF-8, and Mn-ZIF-8 were synthesized by the same recipe with the addition of 0.15 g of corresponding metal source (Co(OAc)_2_·4H_2_O, Cu(OAc)_2_·H_2_O, Ni(OAc)_2_·4H_2_O, Mn(OAc)_2_·4H_2_O).

### Synthesis of CuN_4_/CoN_4_@NC, M_a_N_4_/M_b_N_4_@NC, 600-Cu/Co@NC and 900-Cu/Co@NC

Typically, the as-prepared Cu-ZIF-8 was mixed with 0.02 g Co-Ph and 60 equivalent weights of KCl-KBr salt (KCl: KBr = 1: 3 by weight). The mixture was placed in a tubular furnace and heated to 750 °C at a ramp rate of 2 °C min^–1^ and kept for 60 min under Ar atmosphere. CuN_4_/CoN_4_@NC was obtained after thoroughly washing by deionized water.

The M_a_N_4_/M_b_N_4_@NC composites were synthesized by the same recipe except for using different ZIFs and/or metal-Ph sources (e.g., Co-ZIF-8, Cu-ZIF-8, Ni-ZIF-8, Mn-ZIF-8, Cu-Ph, Co-Ph, and Fe-Ph).

The 600-Cu/Co@NC and 900-Cu/Co@NC were synthesized by the same recipe except for heated to 600 °C and 900 °C, respectively.

### Synthesis of CuN_4_@NC

Typically, the as-prepared Cu-ZIF-8 was mixed with 60 equivalent weights of KCl-KBr salt (KCl: KBr = 1: 3 by weight). The mixture was heated to 750 °C at a ramp rate of 2 °C min^–1^ and kept for 60 min under Ar atmosphere in a tubular furnace. CuN_4_@NC was obtained after thoroughly washing by deionized water.

### Synthesis of CoN_4_@NC

Typically, the as-prepared ZIF-8 was mixed with 0.02 g Co-Ph and 60 equivalent weights of KCl-KBr salt (KCl: KBr = 1: 3 by weight). The mixture was heated to 750 °C at a ramp rate of 2 °C min^–1^ and kept for 60 min under Ar atmosphere in a tubular furnace. CoN_4_@NC was obtained after thoroughly washing by deionized water.

### Synthesis of Co_x_@NC

Typically, 0.02 g Co-Ph was mixed with KCl-KBr salt (KCl: KBr = 1: 3 by weight). The mixture was placed in a tubular furnace and heated to 750 °C at a ramp rate of 2 °C min^–1^ and kept for 60 min under Ar atmosphere. Co_x_@NC was obtained after thoroughly washing with deionized water.

### Synthesis of NC

Typically, the as-prepared ZIF-8 was mixed with 60 equivalent weights of KCl-KBr salt (KCl: KBr = 1: 3 by weight). The mixture was loaded to a tubular furnace and heated to 750 °C at a ramp rate of 2 °C min^–1^ and kept for 60 min under Ar atmosphere. NC was obtained after thoroughly washing by deionized water.

### Synthesis of Cu_x_/Co_x_@NC-750

Typically, a mixture of Cu-ZIF-8 (0.13 g) and Co-Ph (0.02 g) was placed in a tubular furnace and heated to 750 °C using a ramp rate of 2 °C min^–1^ and kept for 60 min under Ar atmosphere to obtain Cu_x_/Co_x_@NC-750.

### Synthesis of Cu-ZIF-8-750 and Co-Ph-750

Typically, Cu-ZIF-8 was placed in a tubular furnace and heated to 750 °C at a ramp rate of 2 °C min^–1^ and kept for 60 min under Ar atmosphere to obtain Cu-ZIF-8-750.

The Co-Ph-750 was prepared according to the same recipe except for using Co-Ph as the precursor.

### Materials characterization

The size and morphology of materials were studied by scanning electron microscopy (SEM) and transmission electron microscopy (TEM). SEM was carried out on a HITACHI SU8220 instrument. TEM and high-angle annular dark-field scanning transmission electron microscopy (HAADF-STEM) were recorded on a FEI Titan Cubed Themis G^2^ 300 S/TEM with a probe corrector and a monochromator at 200 kV. Powder X-ray diffraction (XRD) patterns of the samples were obtained on a Rigaku diffractometer (D/max-IIIA,3 kW) with CuKα radiation (λ = 1.5406 Å) at a voltage of 40 kV and a current of 10 mA at room temperature. Raman spectra were recorded on a LabRAM ARAMIS Raman spectrometer (HORIBA Jobin Yvon). Brunauer EmmettTeller (BET) surface area and pore size measurements were performed on a Micromeritics ASAP 2020 M instrument at 77 K. Before the analysis, the samples were degassed at 50 °C for 12 h. X-ray photoelectron spectroscopy (XPS) was collected on a Thermo Scientific K-Alpha system with the C *1s* peak (284.6 eV) as reference. The metal contents of the samples were determined by ICP-OES on an Optima 8300 instrument. Thermogravimetric analysis (TG) was performed on a NETZSCH STA449C instrument loaded with 5 mg sample using a heating rate of 5 °C/min under argon atmosphere. The C and N elemental contents of the samples were measured on a Euro Vector EA3000 instrument (EA). The X-ray absorption experiments were carried out at the XAS station (BL14W1) of the Shanghai Synchrotron Radiation Facility (SSRF). The electron storage ring was operated at 3.5 GeV. Si (311) double-crystal was used as the monochromator, and the data was collected using solid-state detector under ambient conditions. The beam size was limited by the horizontal and vertical slits with the area of 1 × 4 mm^2^ during XAS measurements.

### Calculation details

Molecular dynamics (MD) simulations were carried out with Forcite Plus of Materials Studio software (Biovia Inc.). The temperature was controlled at 450 K by a Nose thermostat. The dynamics of 10 ps was carried out with NVT ensemble, and the time step was set as 1fs. Van der Waals interactions were calculated by an atom-based method with a cutoff distance of 12.5 Å. Electrostatic interactions were calculated by the Ewald method, which takes a long time but is accurate for long-range interactions. Finally, the change of the model structure in the kinetic process is analyzed to judge the thermodynamic stability of the model at 450 K.

Spin-polarized density functional theory (DFT) calculations were performed with the Vienna ab initio simulation package (VASP), using projector augmented wave (PAW) pseudopotential for the core electrons, a cutoff energy (450 eV) for the valence electrons, and the generalized gradient approximation (GGA) in the form of Perdew-Burke-Ernzerhof (PBE) for the exchange correlation potentials. All models were constructed based on a rectangle graphene supercell with lattice constants *a* and *b* as 12.78 and 12.30 Å, containing 60 carbon atoms. To avoid interactions between adjacent images the c axis of these models is set to be 20 Å. The atoms were relaxed fully until the energy convergence reaches 0.00001 eV and the force acting on each atom was <0.01 eV/Å. The lattice constants of all models are relaxed, as well. Van der Waals interaction was considered at the DFT-D2 level as proposed by Grimme.

Coordinating tendency of metal ions to halogen anions was compared via the adsorption energy of KX ion pairs (X = Cl, Br), which is calculated according to the reaction,

$$\ast+{{{{{\rm{KX}}}}}}=\ast {{{{{\rm{KX}}}}}}$$

$${{{{{{\rm{E}}}}}}}_{{{{{{\rm{ads}}}}}}}={{{{{\rm{E}}}}}}\_(\ast {{{{{\rm{KX}}}}}})-{{{{{\rm{E}}}}}}\_\ast -{{{{{\rm{E}}}}}}\_{{{{{\rm{KX}}}}}}$$

where E_*, E_KX, and E_(*KX) are total energy of catalyst, KX ion pair, and catalyst with KX adsorbed. The leaving of metal ions (Cu, Zn) from the catalyst to molten salts were characterized by energy change of the following reaction,

$$\ast+{{{{{{\rm{K}}}}}}}_{8}{{{{{{\rm{Cl}}}}}}}_{2}{{{{{{\rm{Br}}}}}}}_{6}=\ast ^{\prime}+{{{{{{\rm{K}}}}}}}_{8}{{{{{{\rm{Cl}}}}}}}_{2}{{{{{{\rm{Br}}}}}}}_{6}{\prime}$$

$$\varDelta {{{{{\rm{E}}}}}}={{{{{\rm{E}}}}}}\_(\ast ^{\prime} )+{{{{{\rm{E}}}}}}\_({{{{{{\rm{K}}}}}}}_{8}{{{{{{\rm{C}}}}}}}_{{{{{{\rm{l}}}}}}2}{{{{{{\rm{Br}}}}}}}_{6}{\prime} )-{{{{{\rm{E}}}}}}\_\ast -{{{{{\rm{E}}}}}}\_{{{{{{\rm{K}}}}}}}_{8}{{{{{{\rm{Cl}}}}}}}_{2}{{{{{{\rm{Br}}}}}}}_{6}$$

where * and K_8_Cl_2_Br_6_ represent catalyst with metal ions and pure molten salts, while *‘and K8Cl2Br6’ represent catalyst without a metal ion and molten salts with a metal ion. The insertion of metal ion to the catalyst is defined as the reverse reaction of the above reaction.

### Catalytic reaction of benzaldehyde and 2'-hydroxyacetophenone

In a typical run, benzaldehyde (1 mmol), 2'-hydroxyacetophenone (1.5 mmol), catalyst (1.4 mol% total metal relative to benzaldehyde), and *n*-hexanol (4 mL) were sealed in a high-pressure reactor (NSG25-P5-T3-SS1-SV, Anhui Kemi Machinery Technology Co., Ltd). The reactor was evacuated, refilled with 2 bar O_2_, and heated to 140 °C for 12 h under magnetic stirring. After reaction, the reactor was cooled to room temperature. The catalyst was isolated by centrifugation and directly reused after washing and drying. The product was quantified by a GC-MS spectrometer (Agilent, 7890B GC/5977 A MS) equipped with a DB-35 MS UI capillary column (0.25 mm × 30 m).

The conversion and selectivity are calculated using the following equations:

1 $${Benzaldehyde\; conversion}=\left(1-\frac{{Moles\; of\; benzaldehyde}}{{Moles\; of\; benzaldehyde\; loaded}}\right)\times 100\%$$

2 $${Flavone\; selectivity}=\left(\frac{{Moles\; of\; flavone}}{{Moles\; of\; benzaldehyde\; converted}}\right)\times 100\%$$

3 $${Flavone\; yield}=\left(\frac{{Moles\; of\; flavone}}{{Moles\; of\; benzaldehyde\; loaded}}\right)\times 100\%$$

### Continuous flow synthesis of flavone

The continuous flow reactor is illustrated in the maintext (Fig. 4c). Before reaction, the CuN_4_/CoN_4_@NC powder is pelletized, crushed, and sieved into particles in sizes of 20–30 mesh. And the flowing phase is formulated with reactant (benzaldehyde and 2'-hydroxyacetophenone in a molar ratio of 1:1.2) and *n*-hexanol solvent. In a typical run, the catalyst (100 mg) is loaded in the packed column and heated to 150 °C, followed by simultaneously feeding the flowing phase at 0.5 mL min^–1^ and bubbling the O_2_ at 30 mL (standard temperature and pressure, STP) min^–1^ while maintaining the reaction pressure at 0.6 MPa.

The turnover number (TON) is calculated using the following equation:

4 $$TON=\frac{Moles\,of\,{{{{{\rm{benzaldehyde}}}}}}\,converted}{Moles\,of\,Co}$$

The space time yield (STY) is calculated using the following equation:

5 $${STY}=\frac{{{{{{\rm{Flavone\; mass}}}}}}}{{Catalyst\; mass}}\times \frac{1}{{Reaction\; time}}$$

## Supplementary information

Supplementary Information

Description of Additional Supplementary Files

Supplementary Movie 1

Supplementary Movie 2

Supplementary Movie 3

## Data Availability

The experimental data generated in this study are provided in the Supplementary Information file.
