# Supplementary material for: Atomic design of dual-metal hetero-single-atoms for high-efficiency synthesis of natural flavones
Source: Nat Commun. 2022 Dec 22;13:7873. doi: 10.1038/s41467-022-35598-3 (PMC9780242; doi:10.1038/s41467-022-35598-3)
Supplement: Supplementary file 1 — Supplementary Information [file 41467_2022_35598_MOESM1_ESM.pdf]

Supplementary Information for

Atomic design of dual-metal hetero-single-atoms for high-efficiency synthesis of natural flavones

Xin Zhao[1], Ruiqi Fang[1,*], Fengliang Wang[1], Xiangpeng Kong[2], Yingwei Li[1,3,*]

[1]State Key Laboratory of Pulp and Paper Engineering, School of Chemistry and Chemical Engineering, South China University of Technology, Guangzhou 510640, China

[2]The School of Materials Science and Engineering, Harbin Institute of Technology, Shenzhen 518055, China

[3]South China University of Technology–Zhuhai Institute of Modern Industrial Innovation, Zhuhai, 519175, China

Email: fangrq@scut.edu.cn; liyw@scut.edu.cn

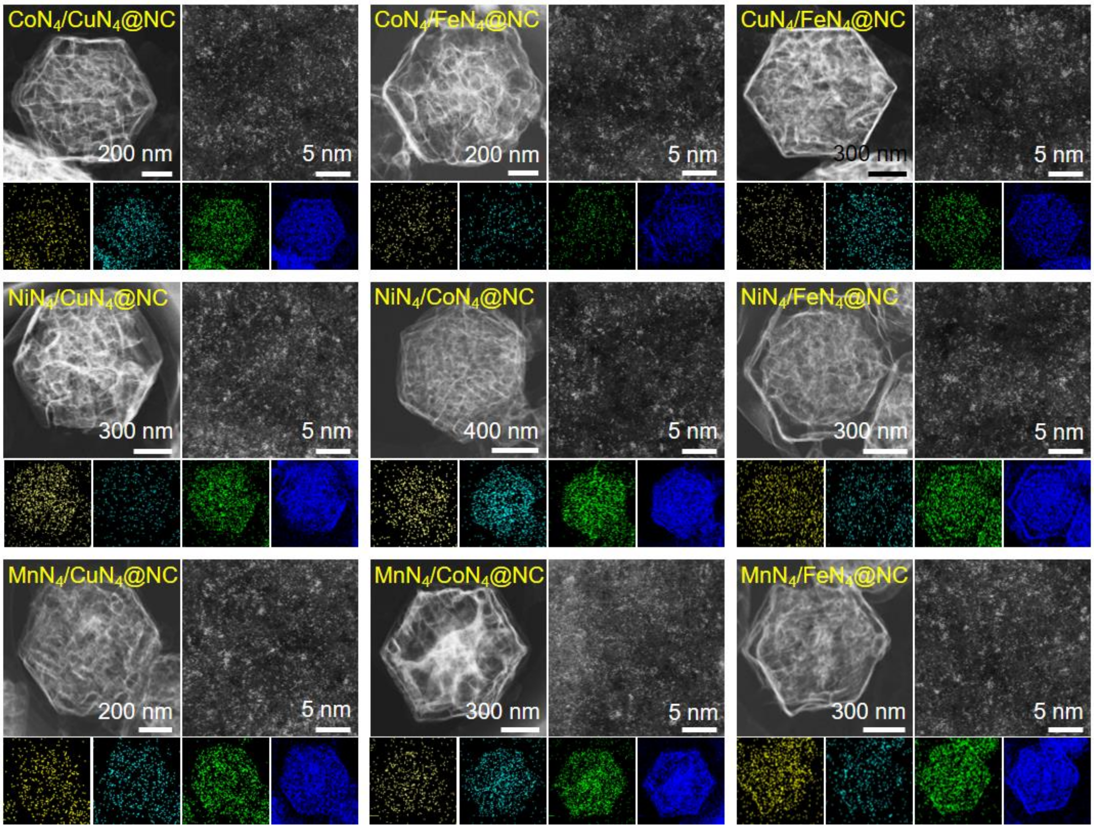

Supplementary Figure 1. HAADF-TEM and elemental mappings of the obtained samples. HAADF-STEM, AC HAADF-STEM, and corresponding elemental mapping (M_a: yellow, M_b: cyan, N: green, C: blue) images of M_aN_4/M_bN_4@NC. The proposed medium-induced infiltration deposition strategy is of considerable flexibility and generality for the fabrication of dual-metal hetero-SAs and SAs. To demonstrate this, various M_a-ZIF-8 MOF templates (M_a = Cu, Co, Ni, Mn) and M_b-Ph precursors (M_b = Co, Cu, Fe) are cross-grouped for the construction of corresponding DSACs, including CoN_4/CuN_4@NC, CoN_4/FeN_4@NC, CuN_4/FeN_4@NC, NiN_4/CuN_4@NC, NiN_4/CoN_4@NC, NiN_4/FeN_4@NC, MnN_4/CuN_4@NC, MnN_4/CoN_4@NC, and MnN_4/FeN_4@NC.

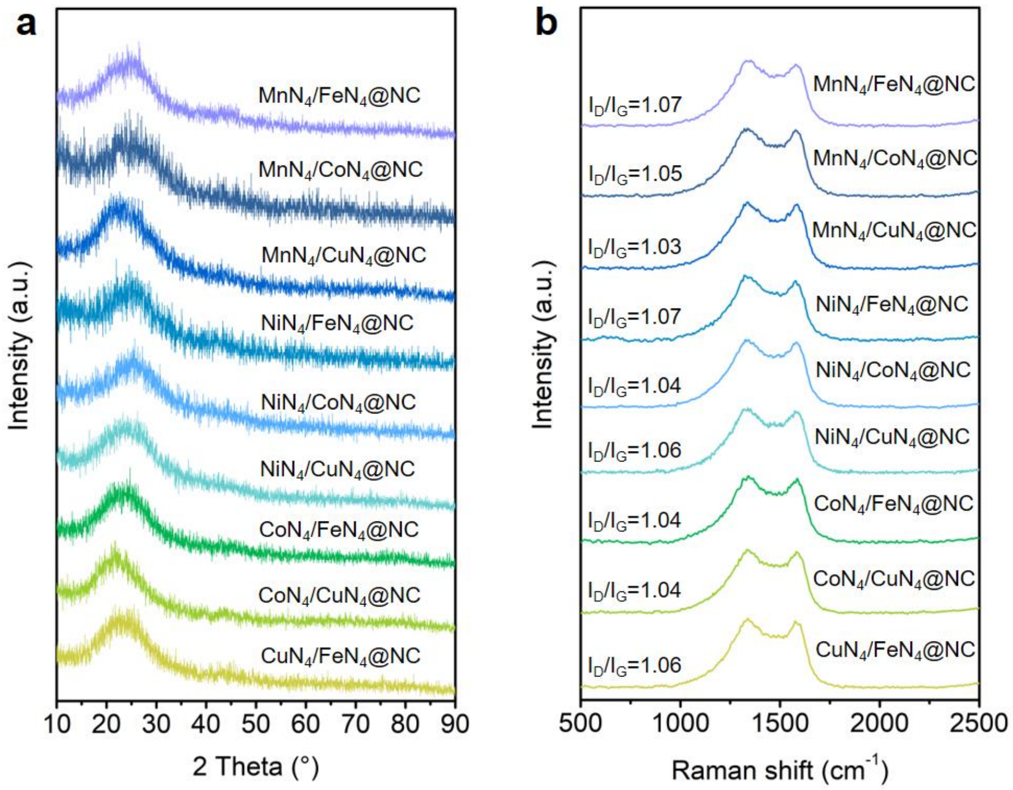

Supplementary Figure 2. XRD patterns and Raman spectra of the obtained samples. (a) XRD patterns, (b) Raman spectra of the obtained samples.

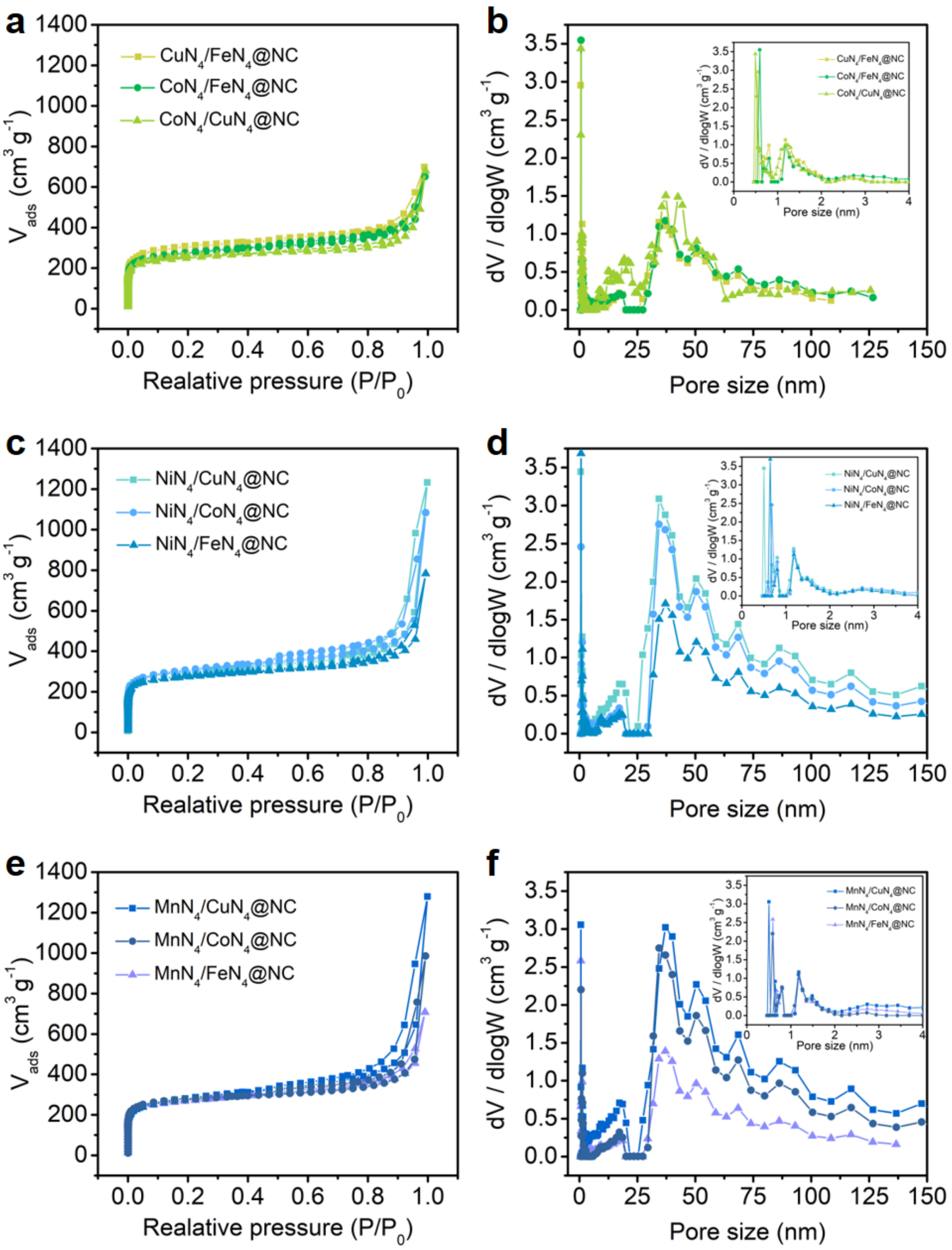

Supplementary Figure 3. Porosity characterizations of the obtained samples. (a, c, e) N_2 adsorption-desorption isotherms, and (b, d, f) corresponding pore-size distributions of the as-synthesized composites. The obtained samples feature hierarchical pores.

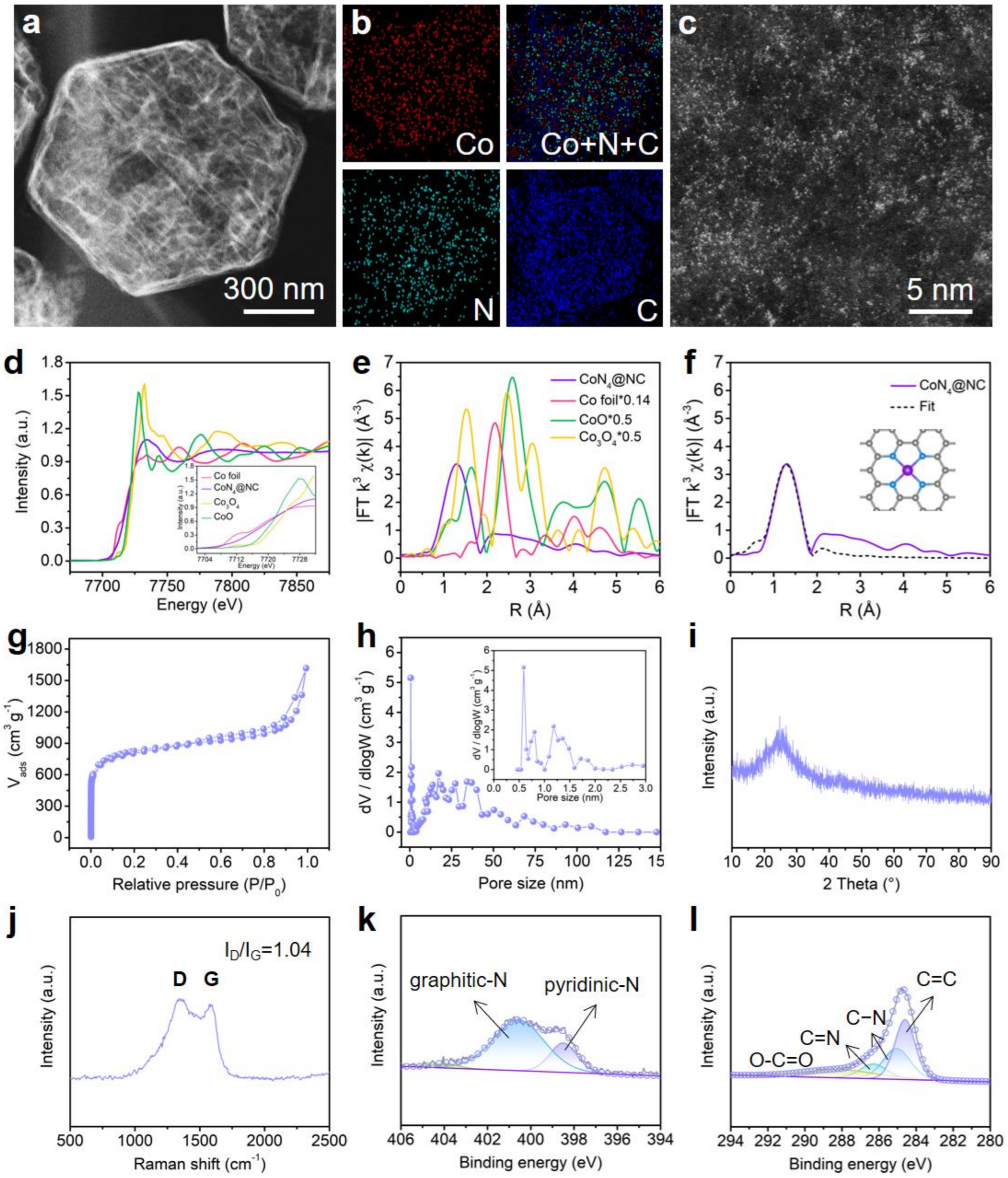

Supplementary Figure 4. Characterizations of CoN_4@NC. (a) HAADF-STEM, (b) corresponding elemental mapping images, and (c) AC HAADF-STEM image of CoN_4@NC. (d) Co K-edge XANES spectra of CoN_4@NC, Co foil, CoO, and Co_3O_4. (e) FT k^3-weighted EXAFS spectra for the Co K-edge of CoN_4@NC, Co foil, CoO, and Co_3O_4. (f) Corresponding Co K-edge EXAFS fitting curves of CoN_4@NC in R space (inset: model of CoN_4 site). (g) N_2 adsorption-desorption isotherms, (h) corresponding pore-size distributions, (i) XRD pattern, (j) Raman spectrum, XPS spectra in N 1s region (k) and C 1s region (l) of the CoN_4@NC.

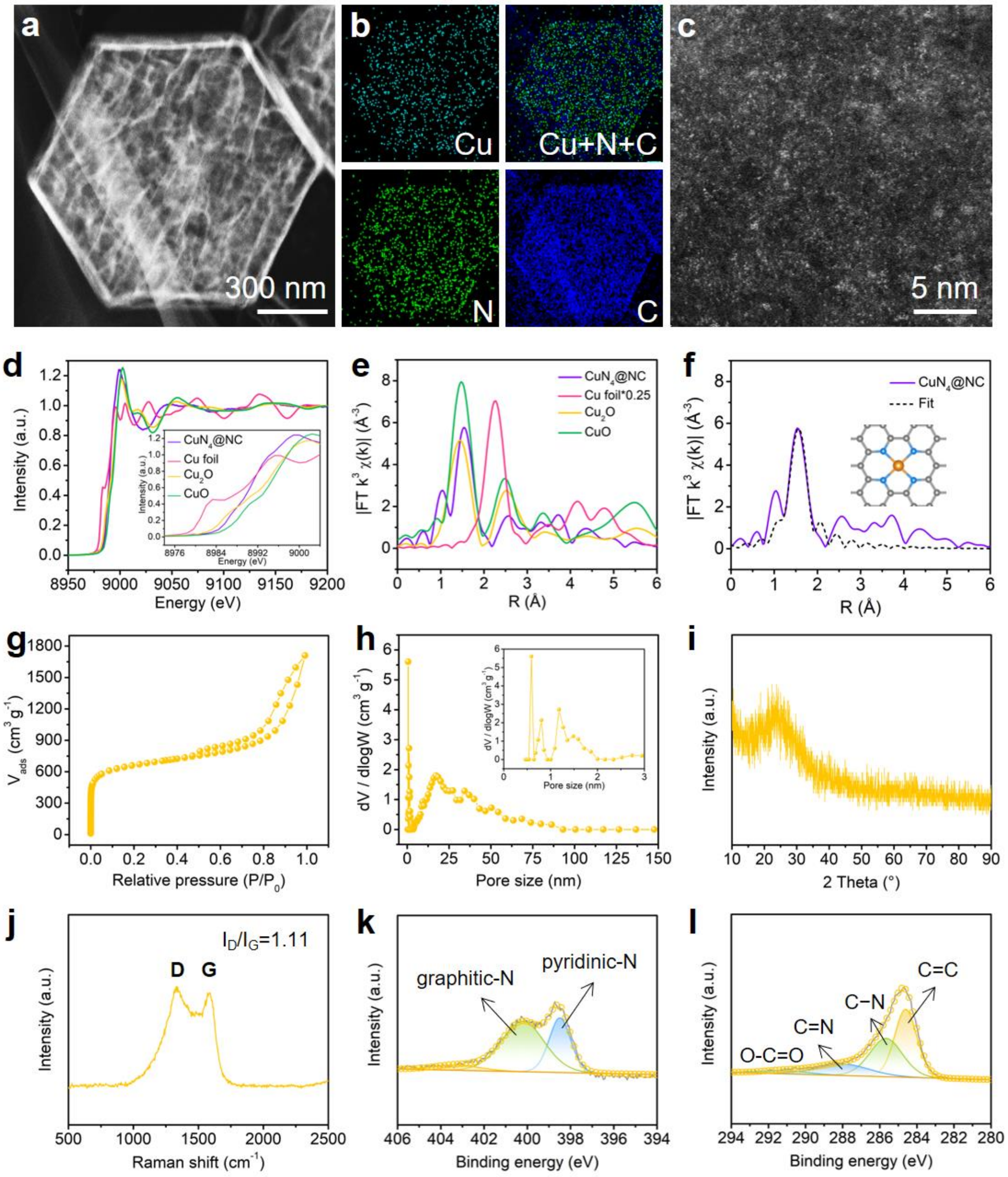

Supplementary Figure 5. Characterizations of CuN_4@NC. (a) HAADF-STEM, (b) corresponding elemental mapping images, and (c) AC HAADF-STEM images of CuN_4@NC. (d) Cu K-edge XANES spectra of CuN_4@NC, Cu foil, Cu_2O, and CuO. (e) Fourier-transformed (FT) k^3-weighted EXAFS spectra for the Cu K-edge of CuN_4@NC, Cu foil, Cu_2O, and CuO. (f) Corresponding Cu K-edge EXAFS fitting curves of CuN_4@NC in R space (inset: model of CuN_4 site). (g) N_2 adsorption-desorption isotherms, (h) corresponding pore-size distributions, (i) XRD pattern, (j) Raman spectrum, XPS spectra in N 1s region (k), and C 1s region (l) of the CuN_4@NC.

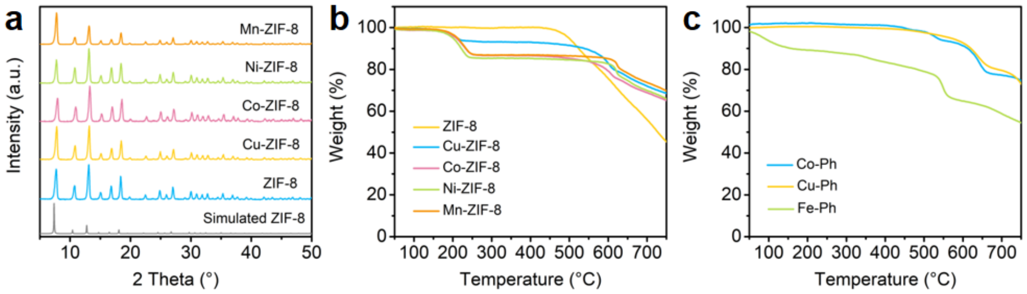

Supplementary Figure 6. XRD patterns and TG curves of the precursors. (a) XRD patterns of the as-synthesized ZIF-8, M-ZIF-8, and the simulated ZIF-8. TG curves of (b) ZIF-8, and (c) phthalocyanine (Ph) samples under Ar.

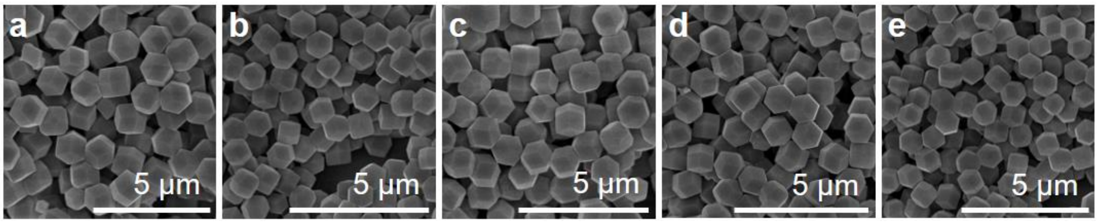

Supplementary Figure 7. SEM images of MOF precursors. SEM images of the as-synthesized (a) ZIF-8, (b) Cu-ZIF-8, (c) Co-ZIF-8, (d) Ni-ZIF-8, and (e) Mn-ZIF-8.

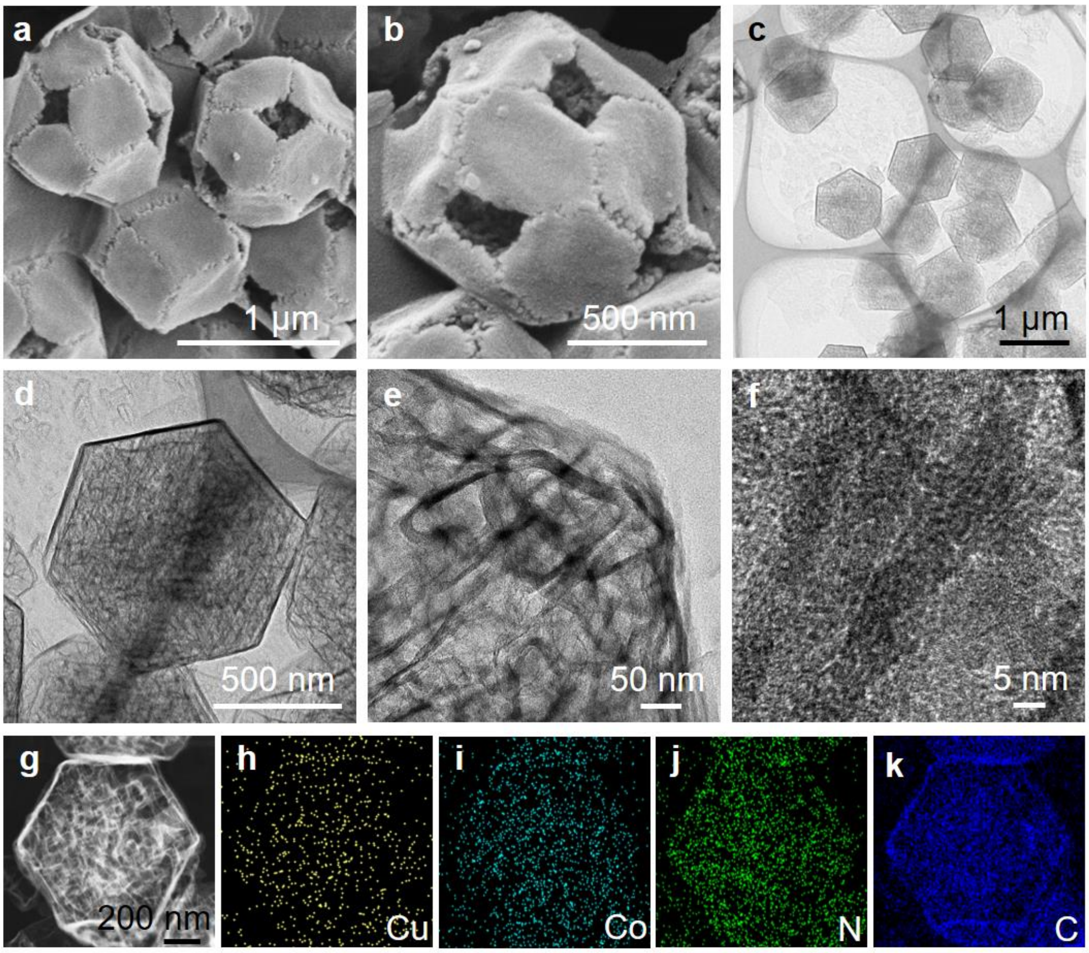

Supplementary Figure 8. Morphology characterizations of CuN_4/CoN_4@NC. (a, b) SEM, (c–e) TEM, (f) HRTEM, (g) HAADF-STEM and (h–k) corresponding elemental mapping images of the CuN_4/CoN_4@NC.

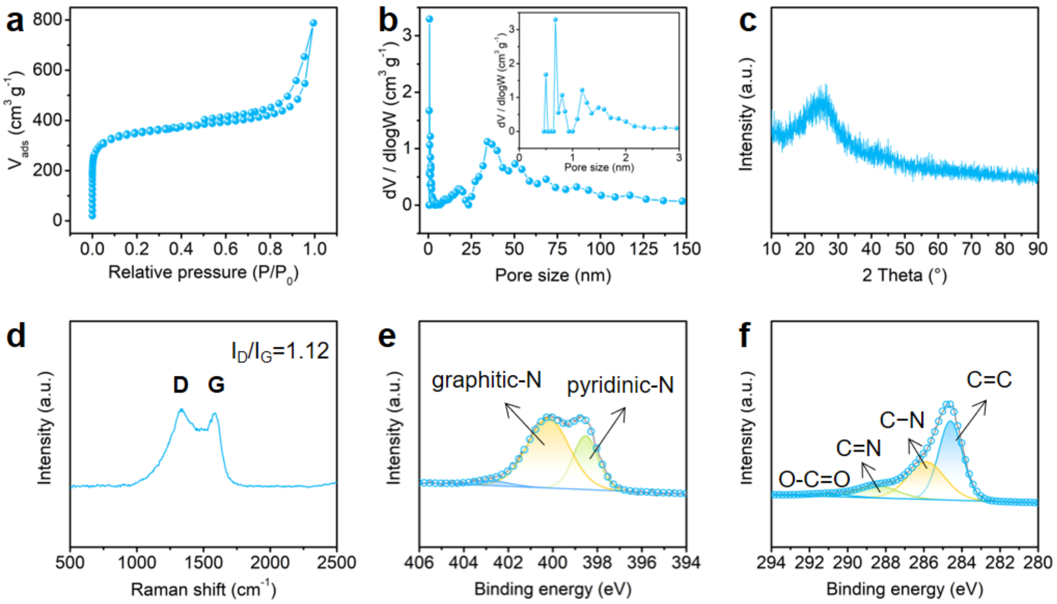

Supplementary Figure 9. Porosity characterizations of CuN_4/CoN_4@NC. (a) N_2 adsorption-desorption isotherms, (b) corresponding pore-size distributions, (c) XRD pattern, (d) Raman spectrum, XPS spectra in N 1s region (e), and C 1s region (f) of the CuN_4/CoN_4@NC.

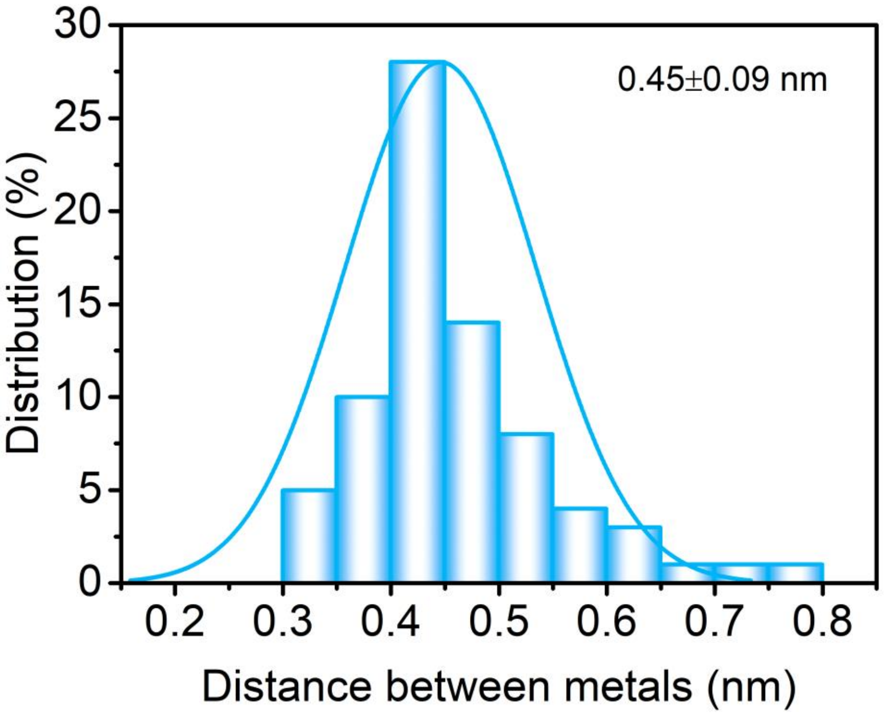

Supplementary Figure 10. Histogram of the distance between metals in CuN_4/CoN_4@NC.

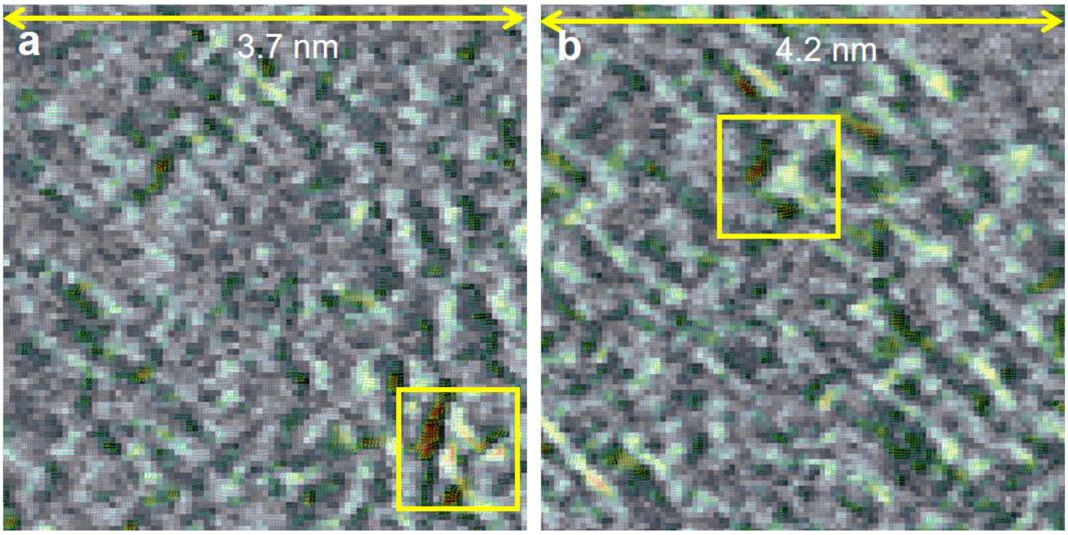

Supplementary Figure 11. DPC-STEM images of the CuN_4/CoN_4@NC. (a, b) Large scale DPC-STEM images of CuN_4/CoN_4@NC, in which the selected area is displayed in Fig. 1h and 1i, respectively.

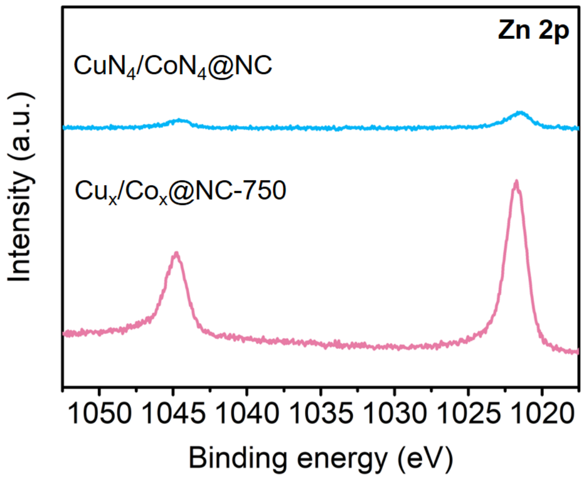

Supplementary Figure 12. Zn 2p XPS spectra of CuN_4/CoN_4@NC and Cu_x/Co_x@NC-750.

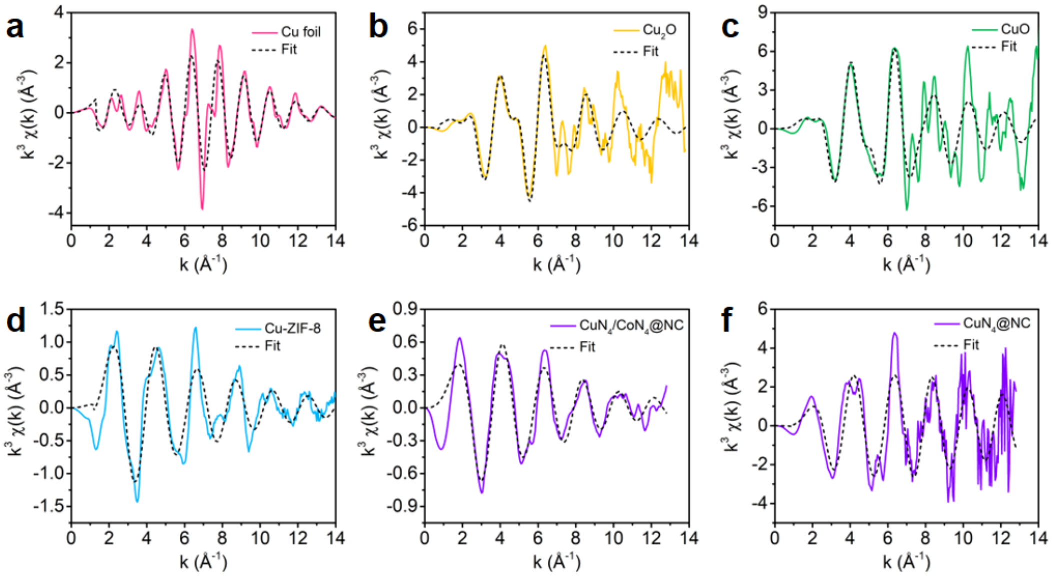

Supplementary Figure 13. Cu K-edge EXAFS fitting curves of CuN_4/CoN_4@NC and references. (a) Cu foil, (b) Cu_2O, (c) CuO, (d) Cu-ZIF-8, (e) CuN_4/CoN_4@NC, and (f) CuN_4@NC.

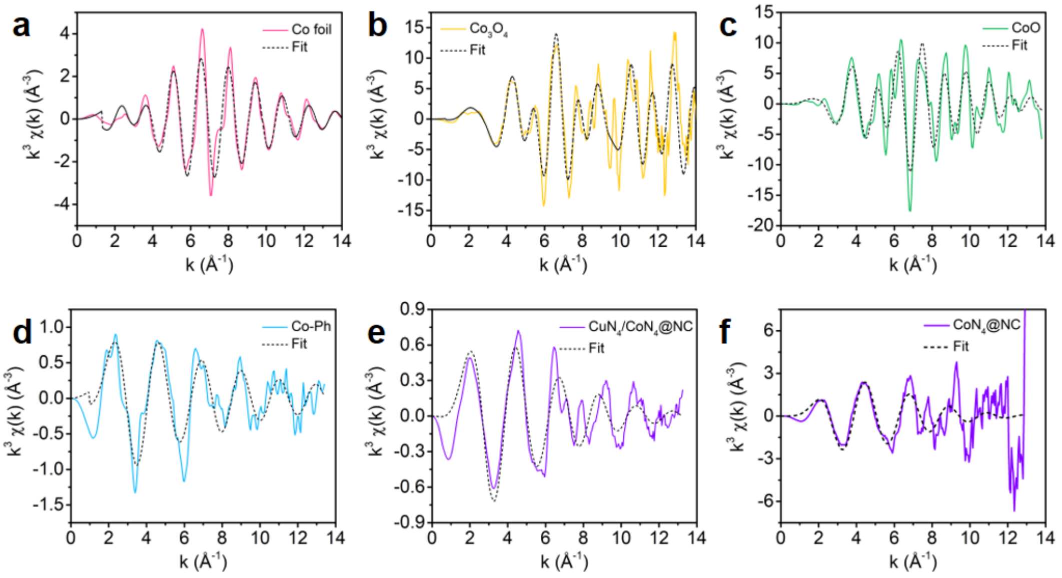

Supplementary Figure 14. Co K-edge EXAFS fitting curves of CuN_4/CoN_4@NC and references. (a) Co foil, (b) Co_3O_4, (c) CoO, (d) Co-Ph, (e) CuN_4/CoN_4@NC, and (f) CoN_4@NC.

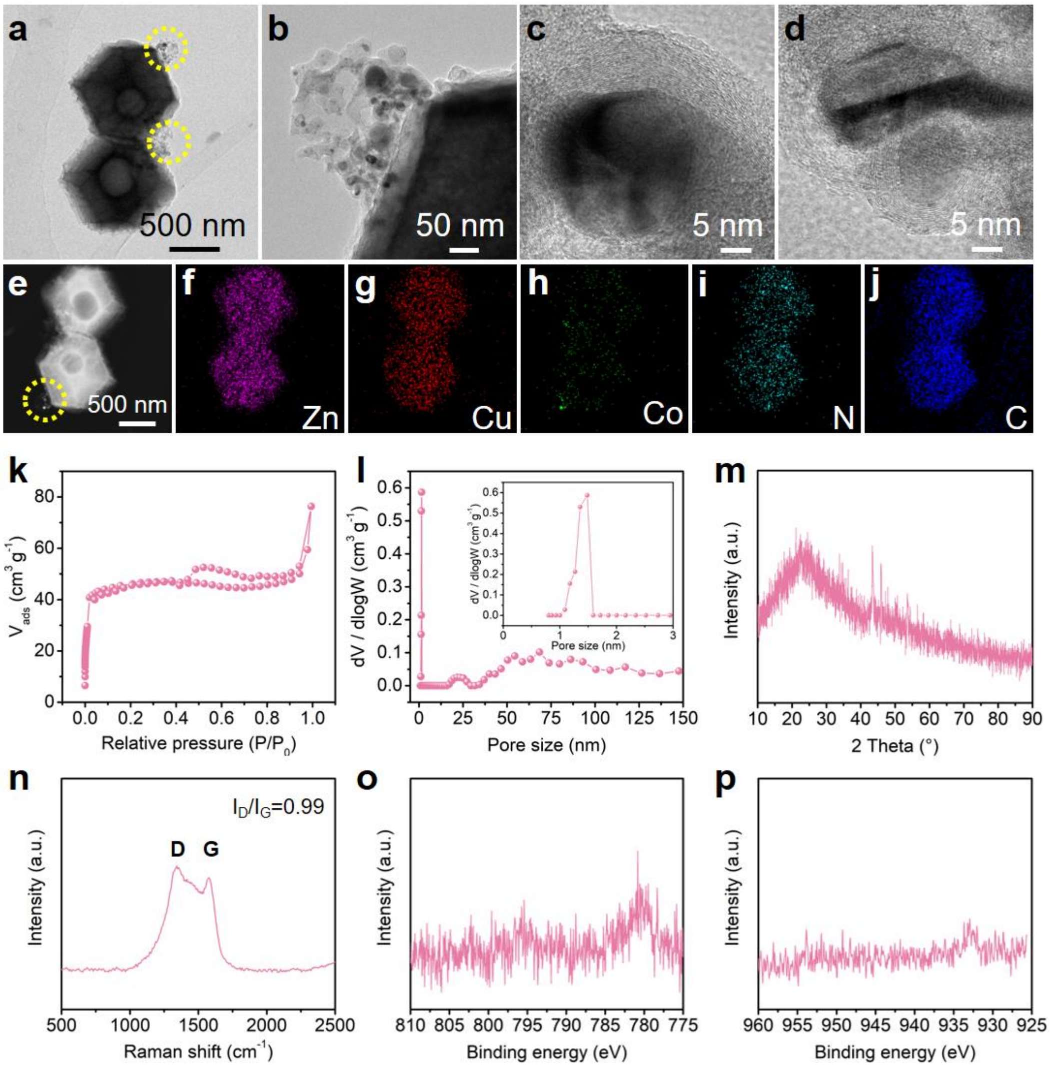

Supplementary Figure 15. Characterizations of Cu_x/Co_x@NC-750. (a, b) TEM, (c, d) HRTEM, (e) HAADF-STEM, (f-j) corresponding elemental mapping images, (k) N_2 adsorption-desorption isotherms, (l) corresponding pore-size distributions, (m) XRD pattern, (n) Raman spectrum, XPS spectra in Co 2p region (o), and Cu 2p region (p) of the Cu_x/Co_x@NC-750.

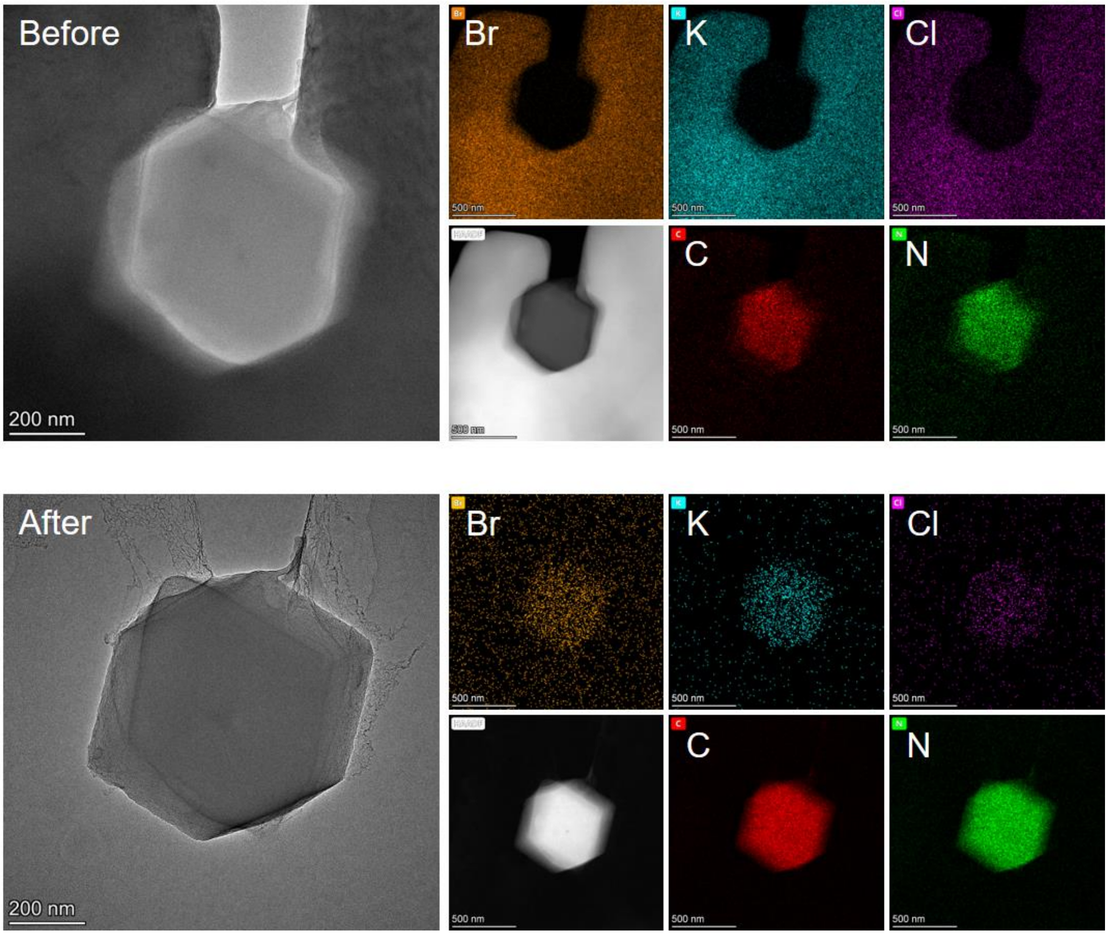

Supplementary Figure 16. *In-situ* TEM observations before and after pyrolysis. TEM, HAADF-STEM, and corresponding elemental mapping images of the *in-situ* TEM observations before and after the pyrolysis process.

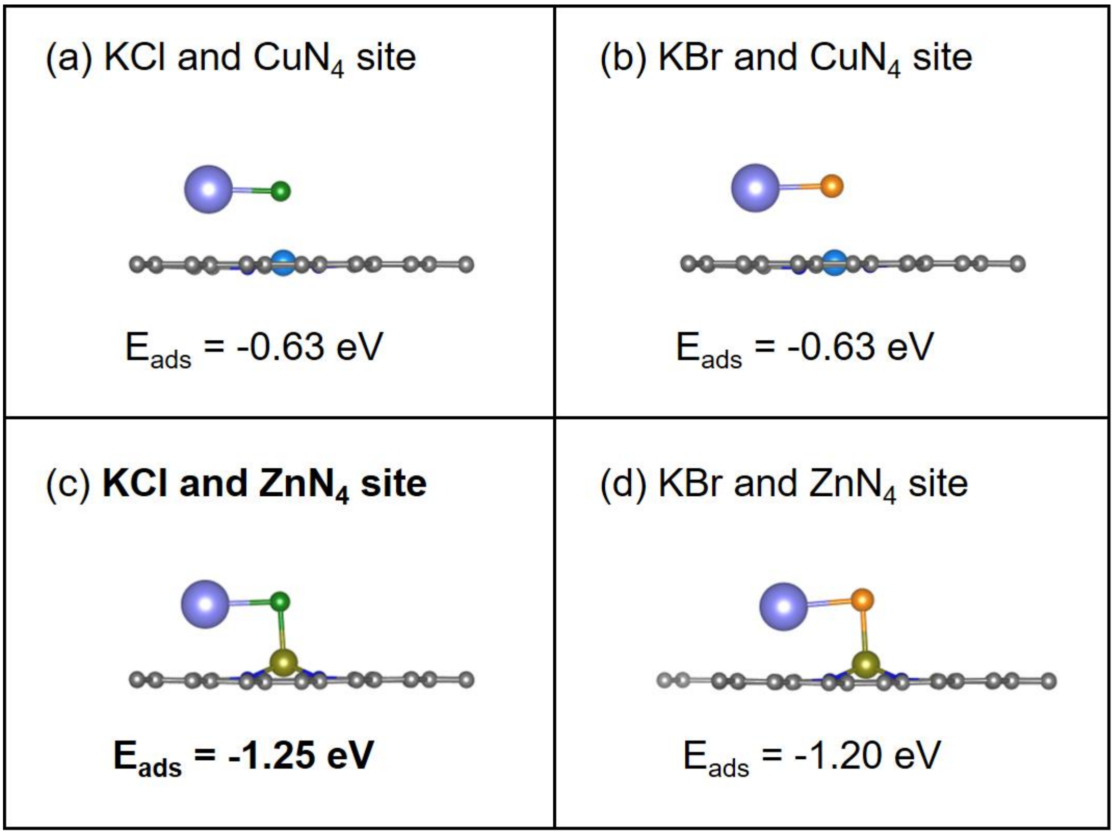

Supplementary Figure 17. The adsorption configuration of molten salt. (a) KCl on CuN_4 site, (b) KBr on CuN_4 site, (c) KCl on ZnN_4 site, (d) KBr on ZnN_4 site.

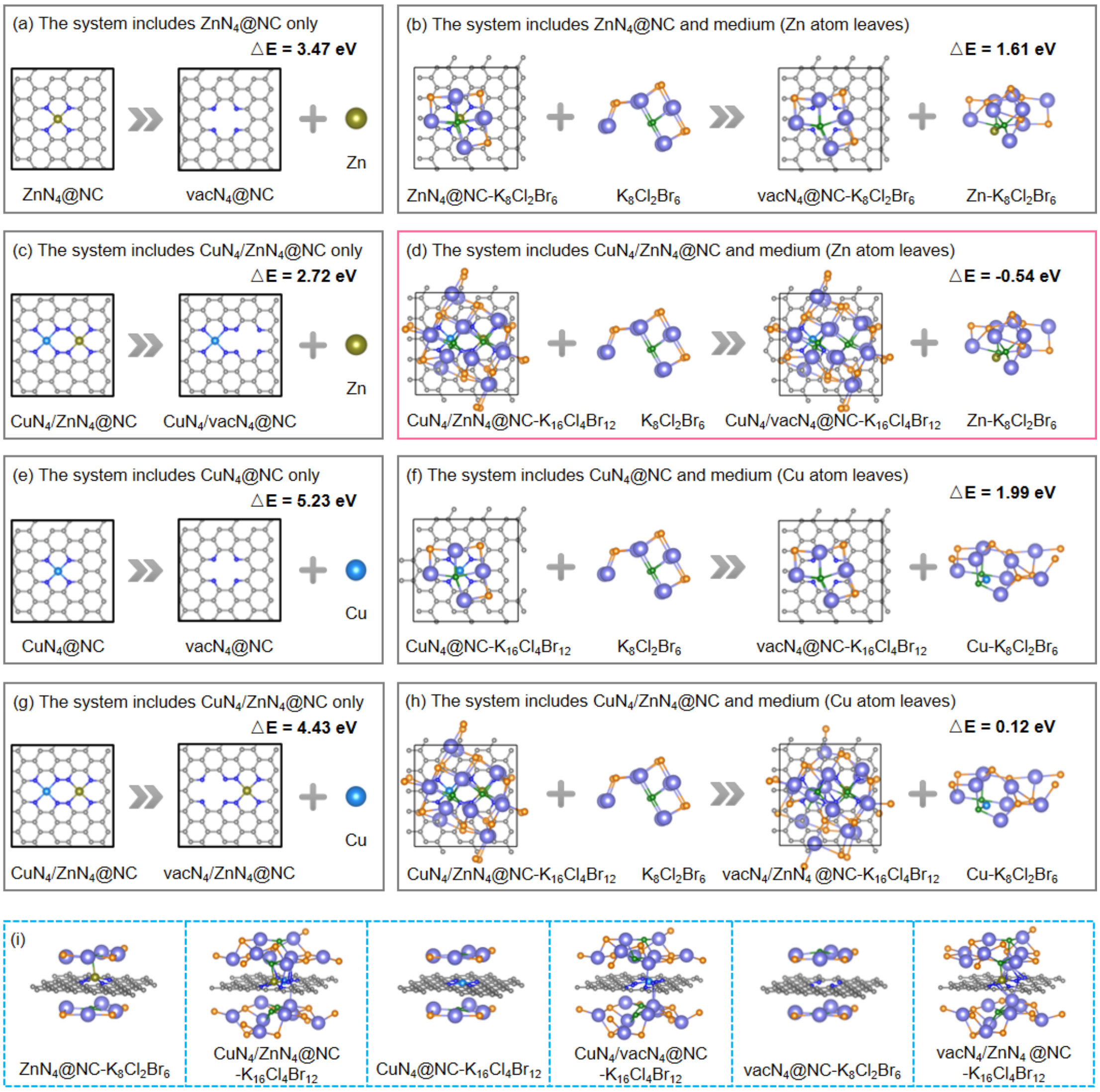

Supplementary Figure 18. Possible conditions and energy barriers during the removal of Cu or Zn atoms from the precursor. The system includes (a) ZnN_4@NC only, (b) ZnN_4@NC and medium, (c) CuN_4/ZnN_4@NC only (Zn leaves), (d) CuN_4/ZnN_4@NC and medium, (e) CuN_4@NC only, (f) CuN_4@NC and medium, (g) CuN_4/ZnN_4@NC only (Cu leaves), (h) CuN_4/ZnN_4@NC and medium (Cu leaves), (i) possible configurations of molten salt and samples.

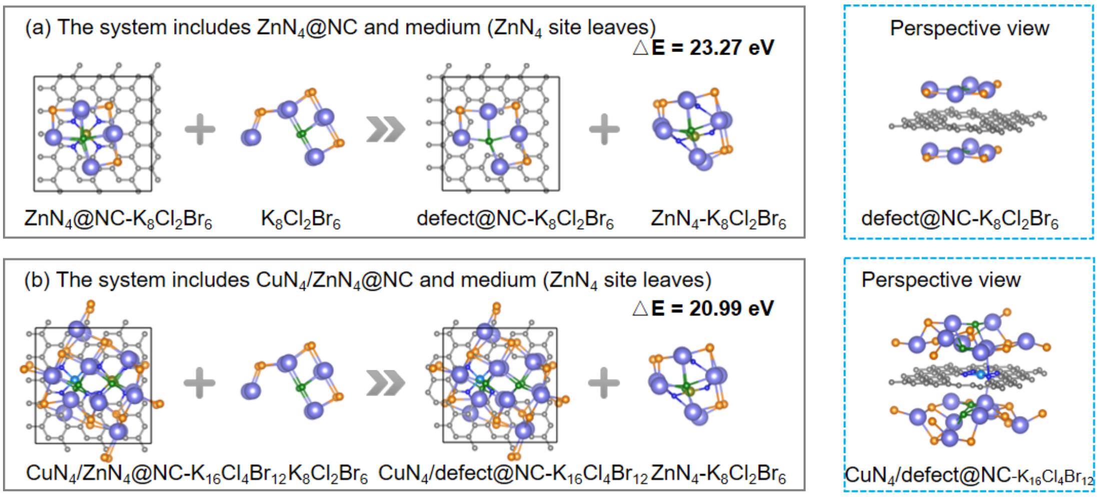

Supplementary Figure 19. Possible conditions during removal of ZnN_4 site from the substrate. The system includes (a) ZnN_4@NC and medium (ZnN_4 site leaves), (b) CuN_4/ZnN_4@NC and medium (ZnN_4 site leaves).

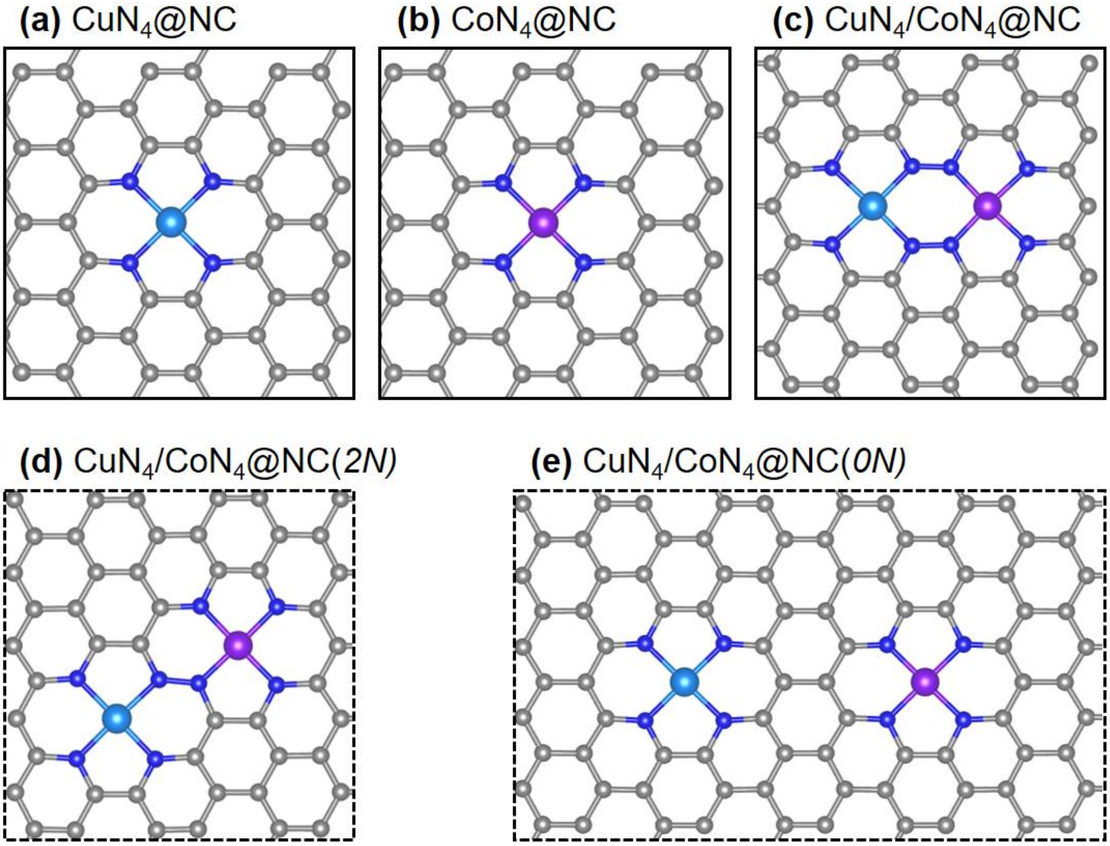

Supplementary Figure 20. DFT models of possible CuN_4 and CoN_4 configurations. (a) CuN_4@NC, (b) CoN_4@NC, (c) CuN_4/CoN_4@NC, (d) CuN_4/CoN_4@NC(*2N*), and (e) CuN_4/CoN_4@NC(*0N*). Cu (sky blue), Co (purple), N (blue), and C (gray).

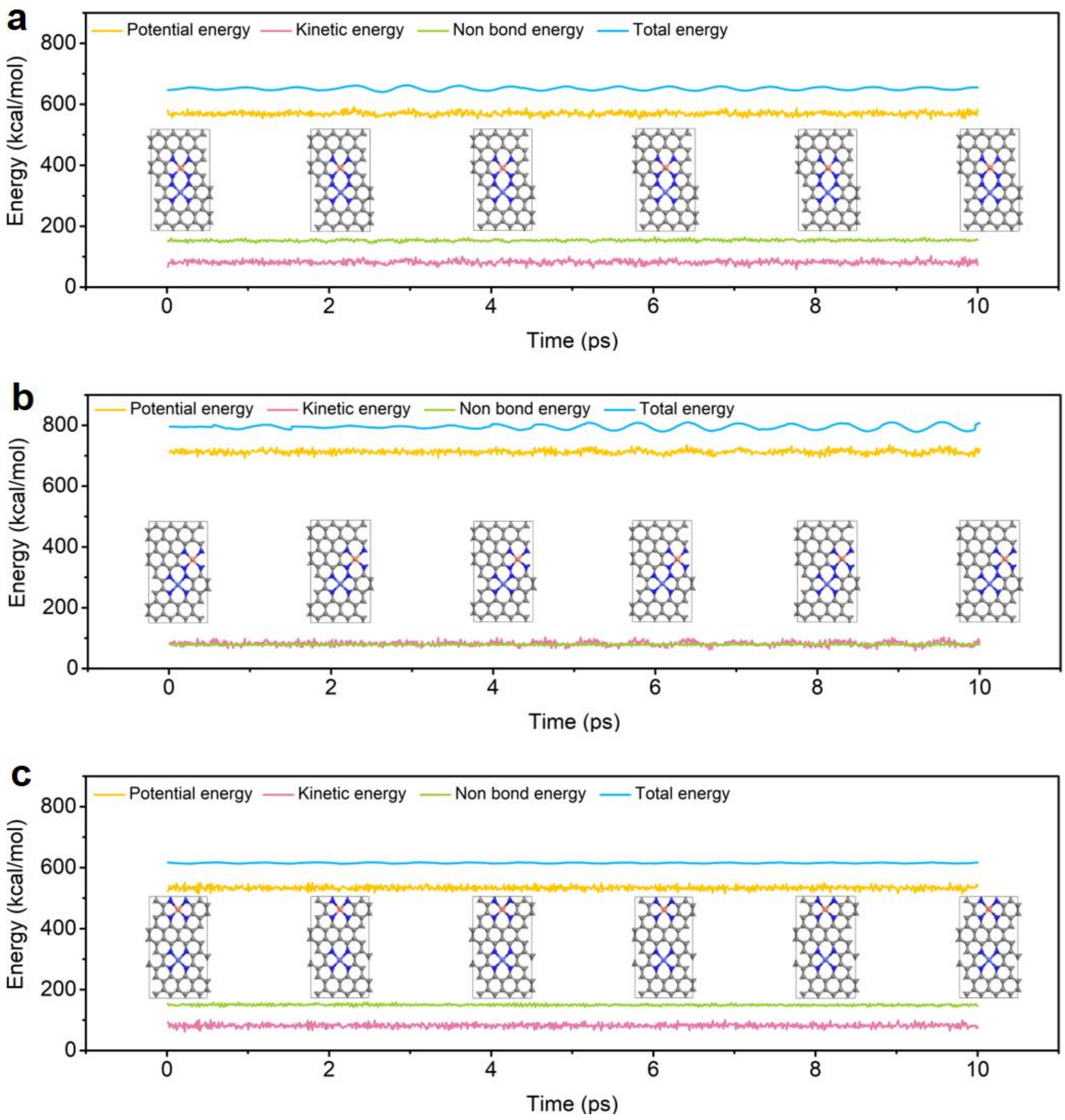

Supplementary Figure 21. The molecular dynamics simulations of the obtained samples. The DFT profile of total energy, potential energy, kinetic energy, and non-bounded energy vs. time at 450 K of (a) CuN_4/CoN_4@NC, (b) CuN_4/CoN_4@NC(2N) and (c) CuN_4/CoN_4@NC(0N) structures. The insets represent the structures at 0, 2, 4, 6, 8, and 10 ps. Cu (orange), Co (light purple), N (blue), and C (gray).

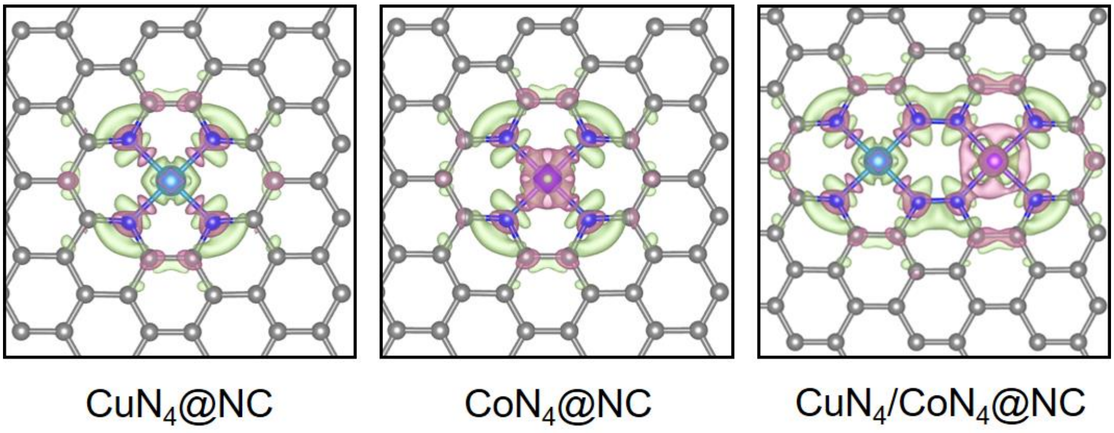

Supplementary Figure 22. Calculated charge distributions of different samples. Top view of the differential charge densities of CuN_4@NC, CoN_4@NC, and CuN_4/CoN_4@NC; pink and green isosurfaces with an isosurface level of 0.004 e/a_0^3 represent electron accumulation and depletion areas, respectively. Cu (sky blue), Co (purple), N (blue), and C (gray). For single metallic CuN_4@NC and CoN_4@NC, electron accumulations are observed atop the Co atom. In terms of CuN_4/CoN_4@NC, synergistic interactions between the adjacent CuN_4 and CoN_4 sites lead to modified electron distributions, the electron depletion over both CuN_4 and CoN_4 are remarkably reduced, while electron accumulations atop Co are also deconcentrated.

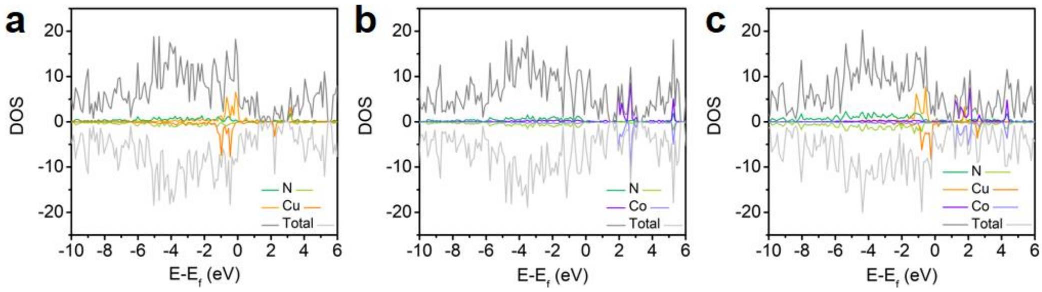

Supplementary Figure 23. Density of states of different samples. Density of states of (a) CuN_4@NC, (b) CoN_4@NC, and (c) CuN_4/CoN_4@NC. The density of states (DOS) is simulated concentrating on the states of Cu and Co *d* orbitals. In CuN_4@NC, the dominant contribution of Cu DOS is away from the Fermi level since the *d* orbitals are fully occupied by electrons. In CoN_4@NC, the dominant contribution of the Co DOS is near the Fermi level due to its partially filled *d* orbitals.

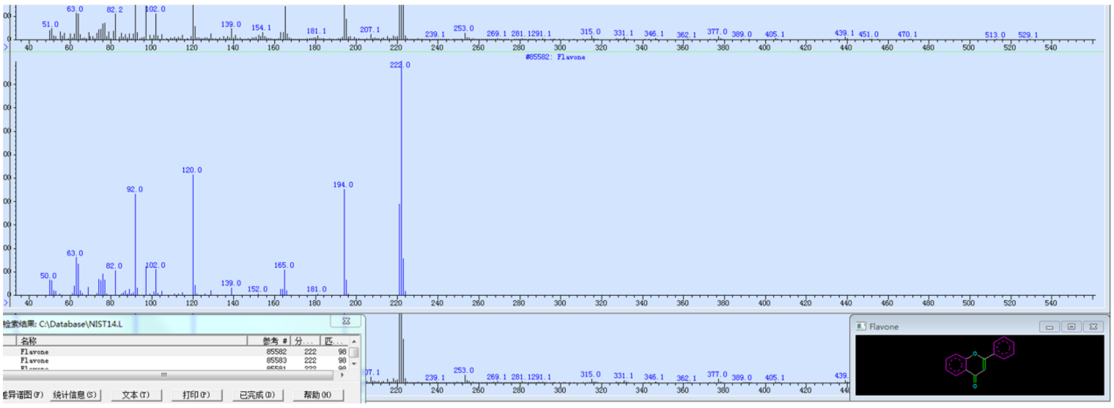

Supplementary Figure 24. GC–MS spectrum of flavone. A representative GC–MS spectrum for the determination of the products from one-pot cascade reaction of benzaldehyde and 2'-hydroxyacetophenon.

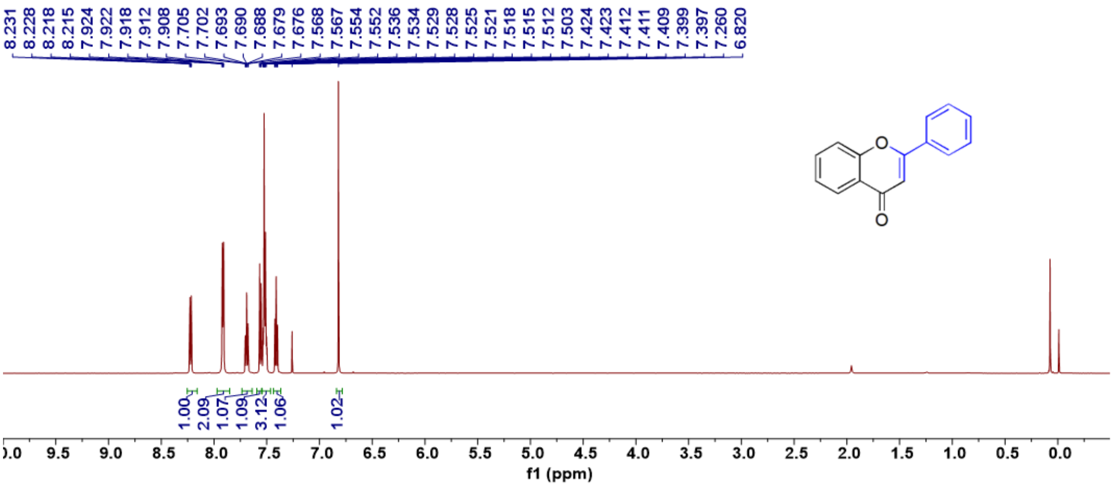

Supplementary Figure 25. ^{1}H-NMR spectrum of the as-synthesized flavone.

^{1}H-NMR (600 MHz, Chloroform-*d*) δ 8.22 (dd, *J* = 7.9, 1.7 Hz, 1H), 7.97 – 7.85 (m, 2H), 7.69 (ddd, *J* = 8.6, 7.1, 1.7 Hz, 1H), 7.56 (dd, *J* = 8.5, 1.0 Hz, 1H), 7.55 – 7.46 (m, 3H), 7.41 (ddd, *J* = 8.1, 7.1, 1.1 Hz, 1H), 6.82 (s, 1H).

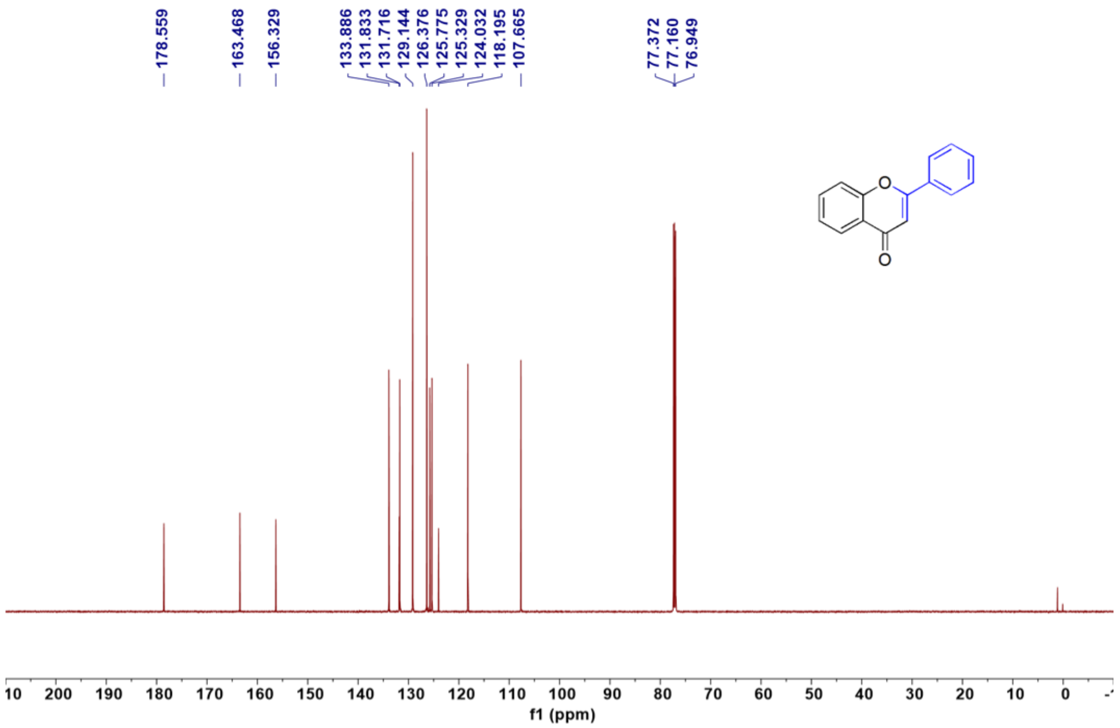

Supplementary Figure 26. ^{13}C-NMR spectrum of the as-synthesized flavone.

^{13}C-NMR (151 MHz, Chloroform-*d*) δ 178.56, 163.47, 156.33, 133.89, 131.83, 131.72, 129.14, 126.38, 125.78, 125.33, 124.03, 118.19, 107.67.

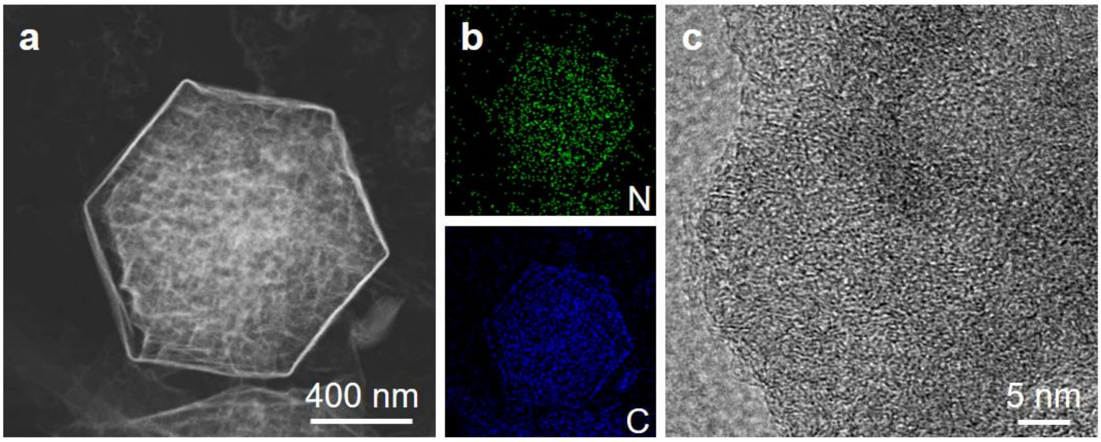

Supplementary Figure 27. Morphology characterizations of NC. (a) HAADF-STEM, (b) corresponding elemental mapping images, and (c) HR-TEM image of the as-prepared NC.

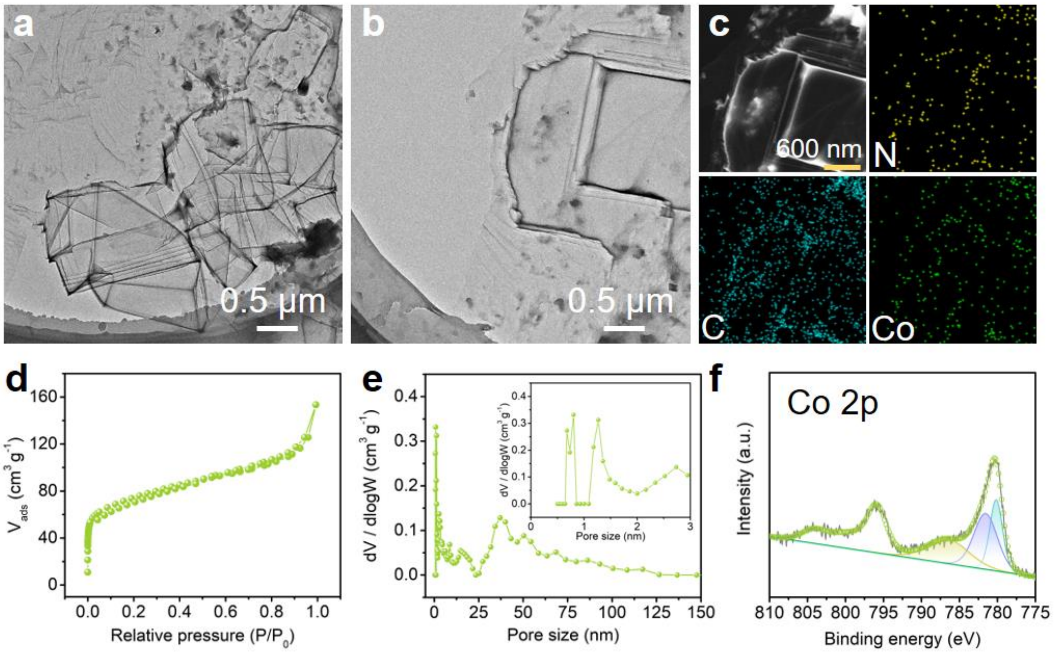

Supplementary Figure 28. Characterizations of Co_x@NC. (a, b) TEM, (c) HAADF-STEM and corresponding elemental mapping images, (d) N_2 adsorption-desorption isotherms, (e) corresponding pore-size distributions, and (f) XPS spectrum in Co 2p region of the Co_x@NC.

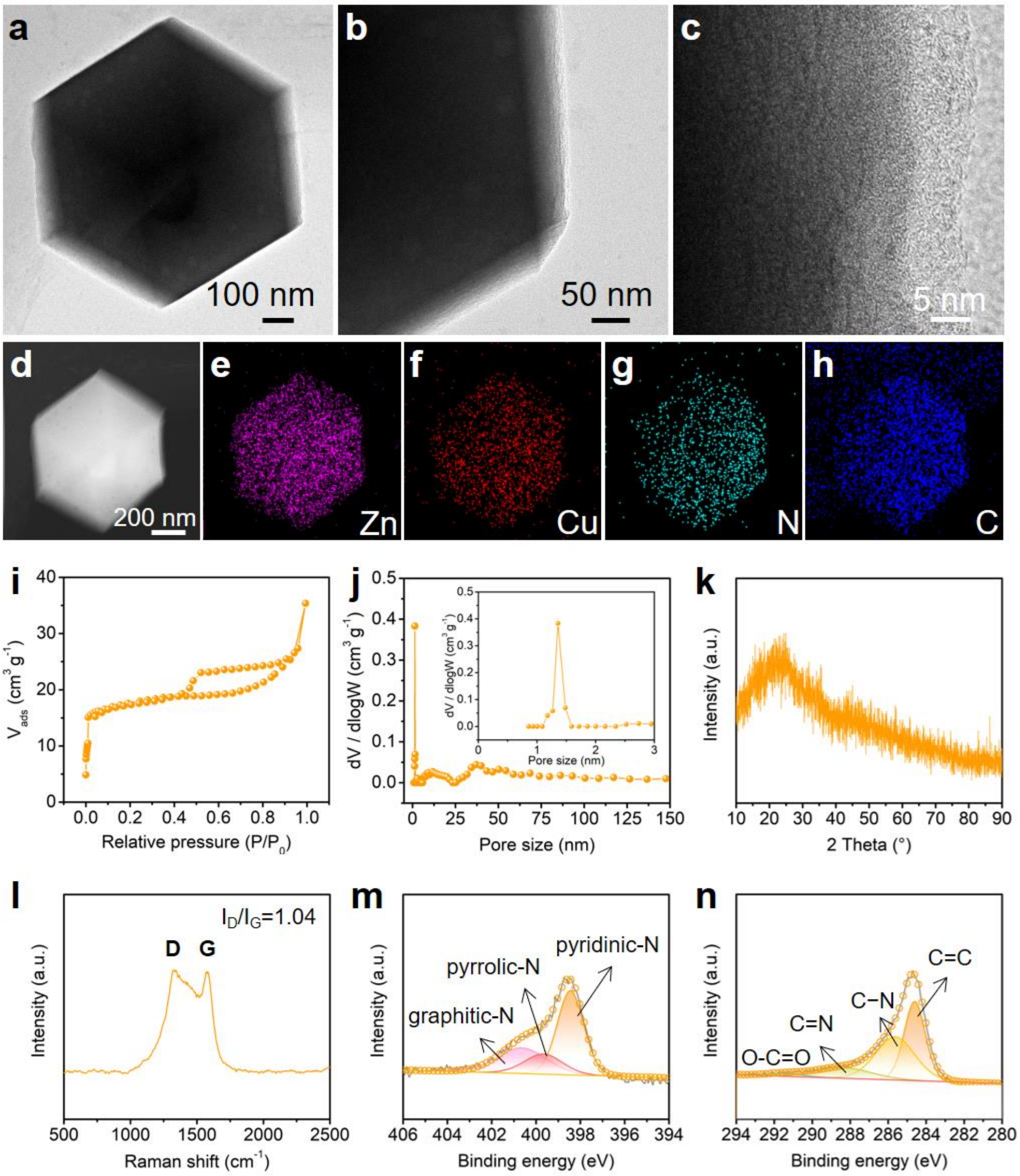

Supplementary Figure 29. Characterizations of Cu-ZIF-8-750. (a, b) TEM, (c) HRTEM, (d) HAADF-STEM, (e-h) corresponding elemental mapping images, (i) N_2 adsorption-desorption isotherms, (j) corresponding pore-size distributions, (k) XRD pattern, (l) Raman spectrum, XPS spectra in N 1s region (m), and C 1s region (n) of the Cu-ZIF-8-750.

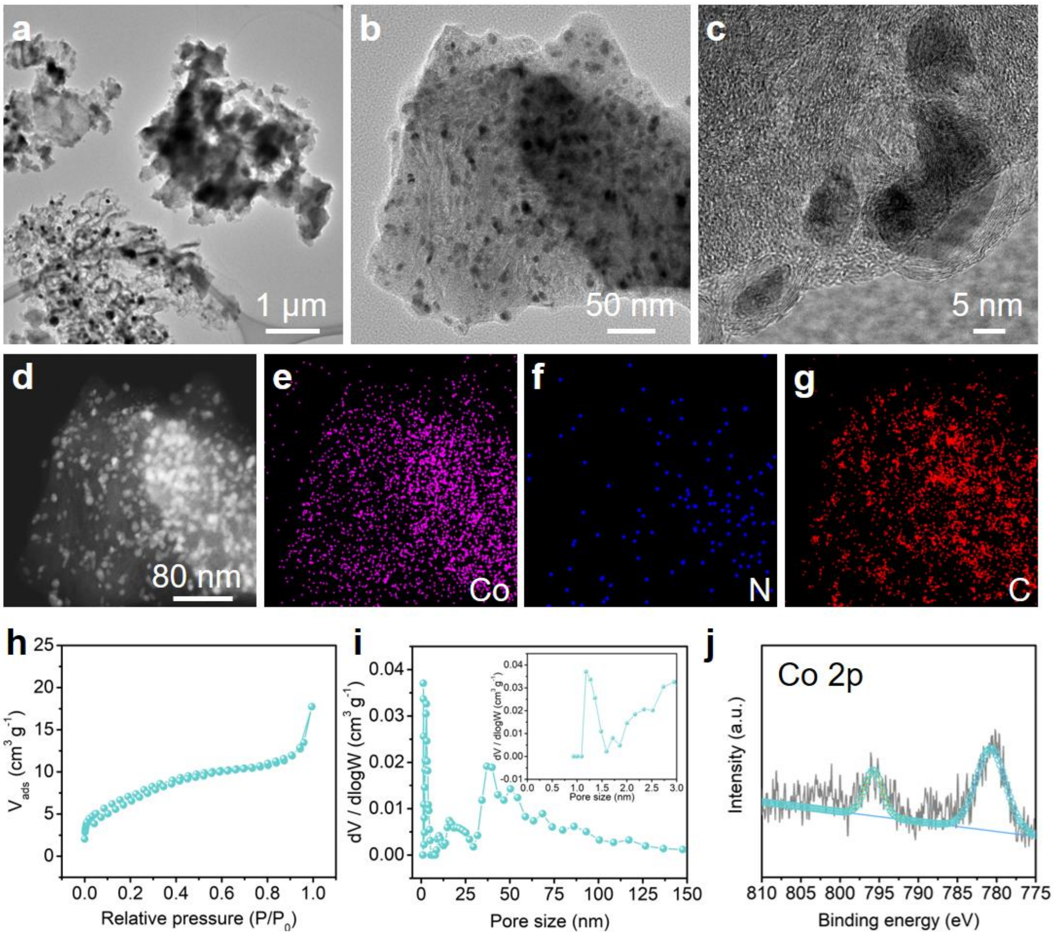

Supplementary Figure 30. Characterizations of Co-Ph-750. (a, b) TEM, (c) HRTEM, (d) HAADF-STEM, (e–g) corresponding elemental mapping images, (h) N_2 adsorption-desorption isotherms, (i) corresponding pore-size distributions, and (j) XPS spectrum in Co 2p region of the Co-Ph-750.

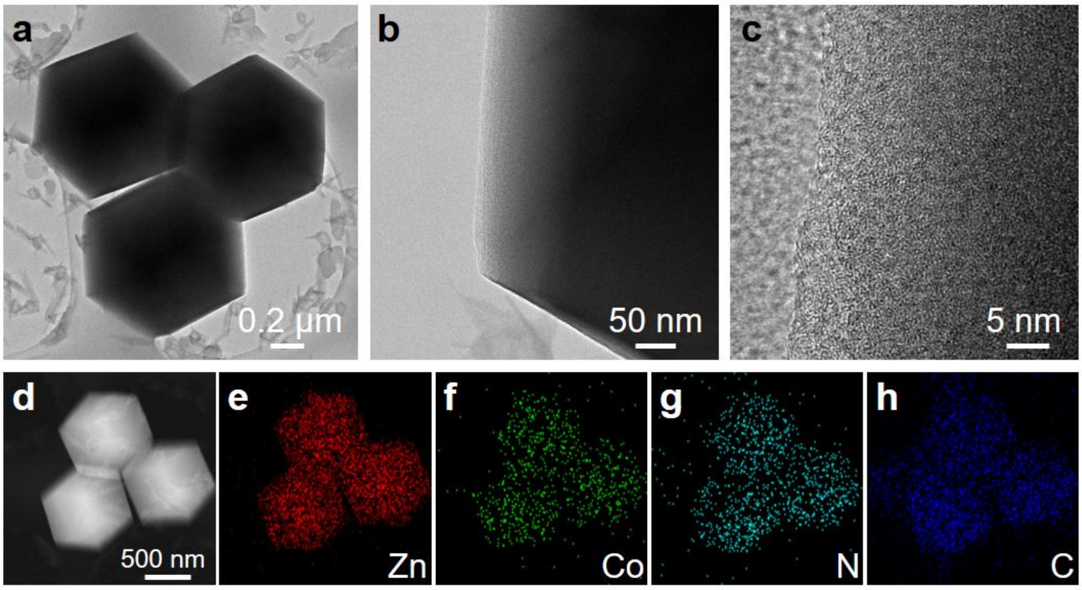

Supplementary Figure 31. Morphology characterizations of Co-ZIF-8-750. (a, b) TEM, (c) HRTEM, (d) HAADF-STEM, (e-h) corresponding elemental mapping images of the as-prepared Co-ZIF-8-750.

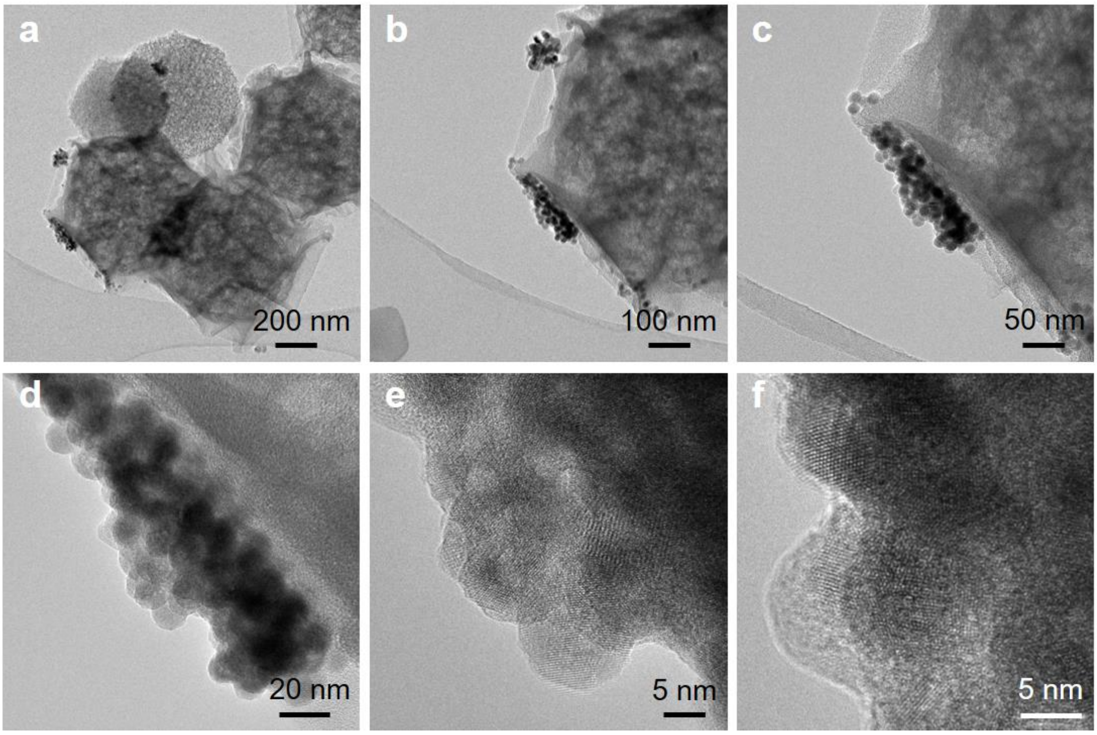

Supplementary Figure 32. Morphology characterizations of 600-Cu/Co@NC. (a–d) TEM, (e, f) HR-TEM images of the 600-Cu/Co@NC.

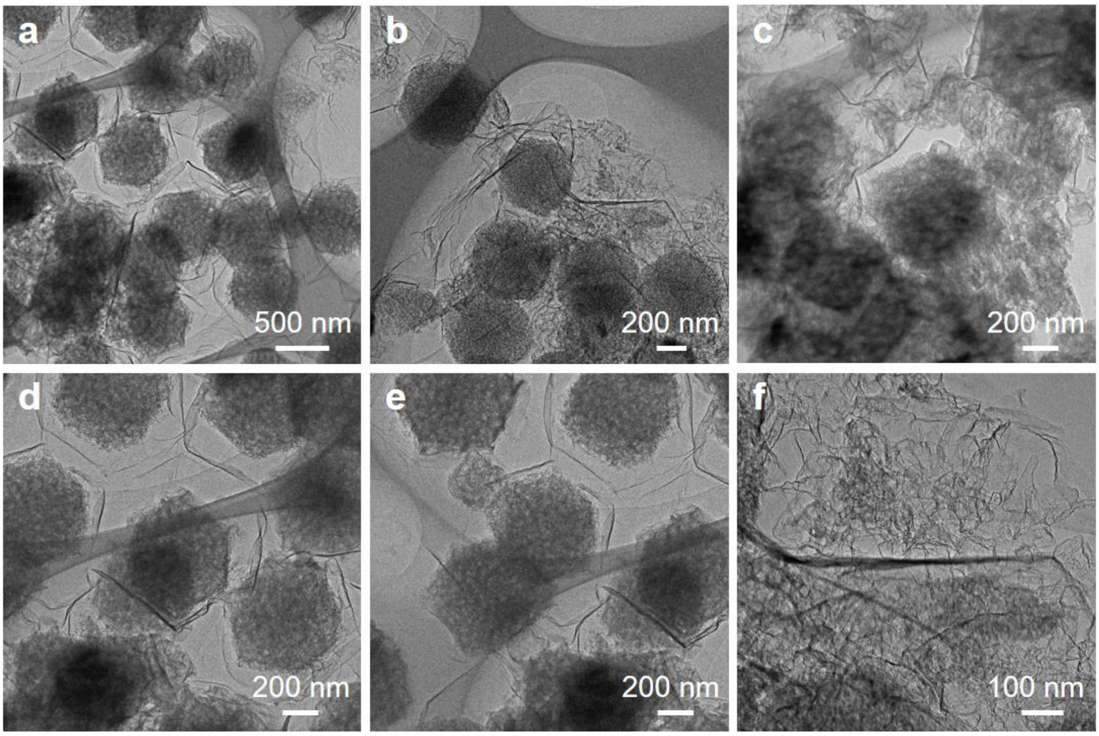

Supplementary Figure 33. Morphology characterizations of 900-Cu/Co@NC. (a–f) TEM images of randomly selected areas of the 900-Cu/Co@NC.

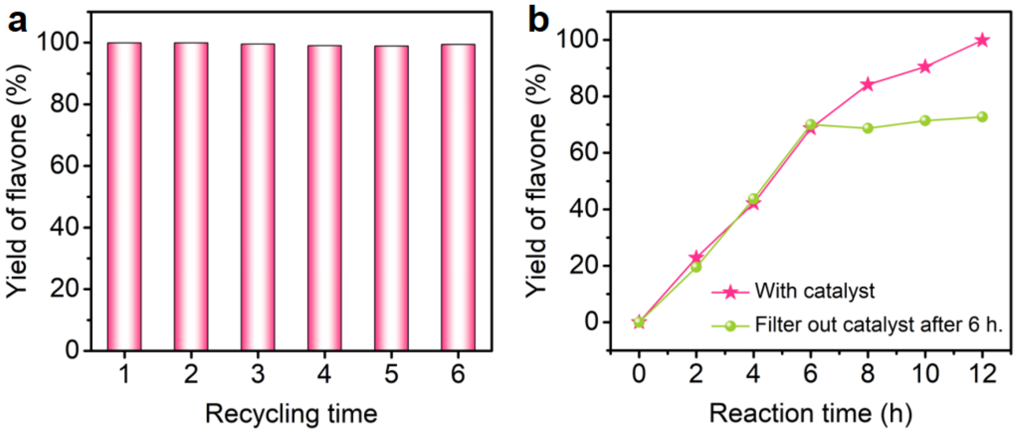

Supplementary Figure 34. Results of reusability and hot filtration experiments. (a) Reusability results of the CuN_4/CoN_4@NC catalyst in the synthesis of flavone. Reaction conditions: benzaldehyde (1 mmol), 2'-hydroxyacetophenone (1.5 mmol), catalyst (total metal, 1.4 mol% relative to benzaldehyde), *n*-hexanol (4 mL), O_2 (2 bar), 140 °C, 12 h. Conversion and yield were determined by GC–MS based on benzaldehyde. (b) The hot filtration experiment results over CuN_4/CoN_4@NC.

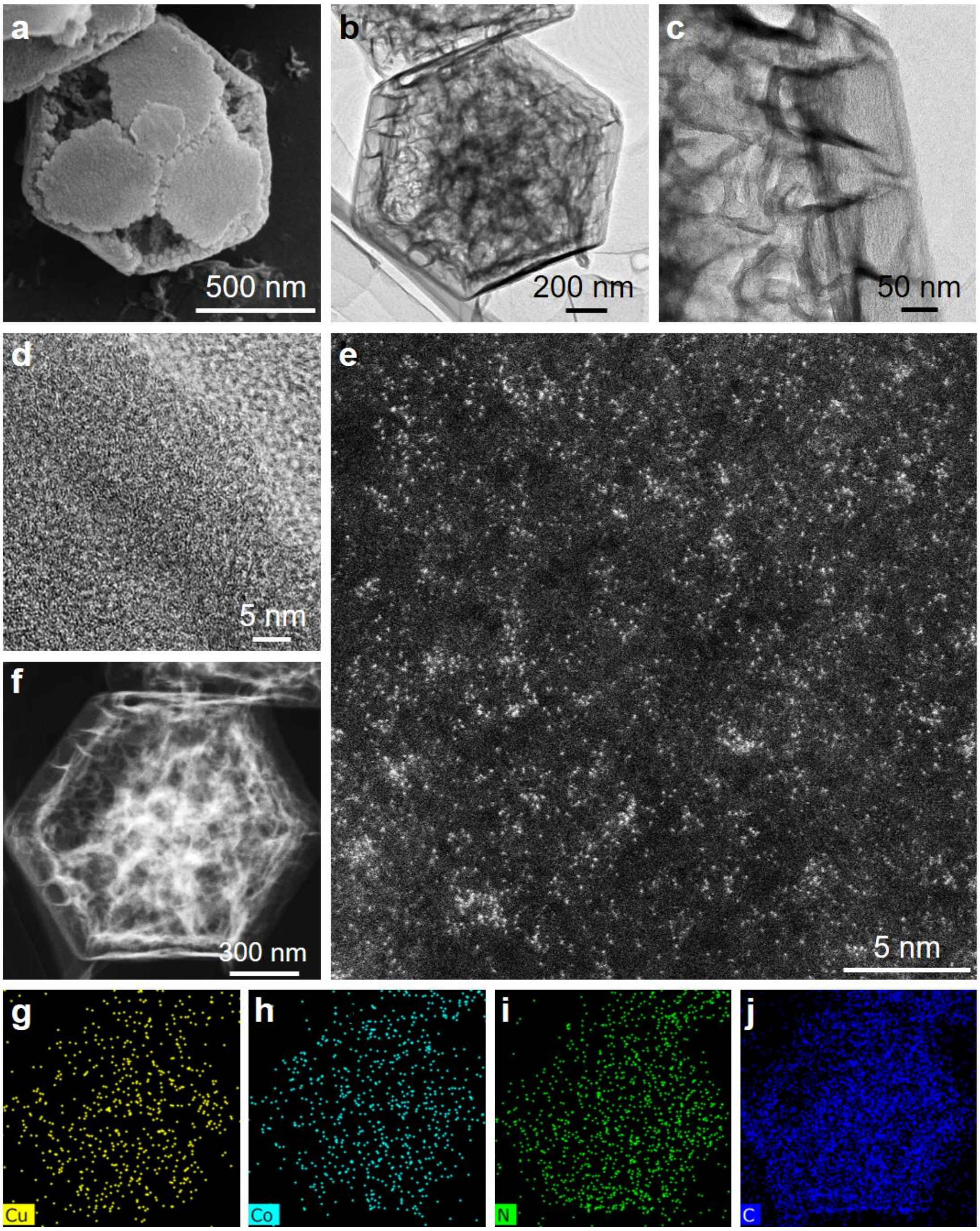

Supplementary Figure 35. Morphology characterizations of the recycled CuN_4/CoN_4@NC. (a) SEM, (b, c) TEM, (d) HRTEM, (e) AC HAADF-STEM, (f) HAADF-STEM, and (g–j) corresponding elemental mapping images of the recycled CuN_4/CoN_4@NC.

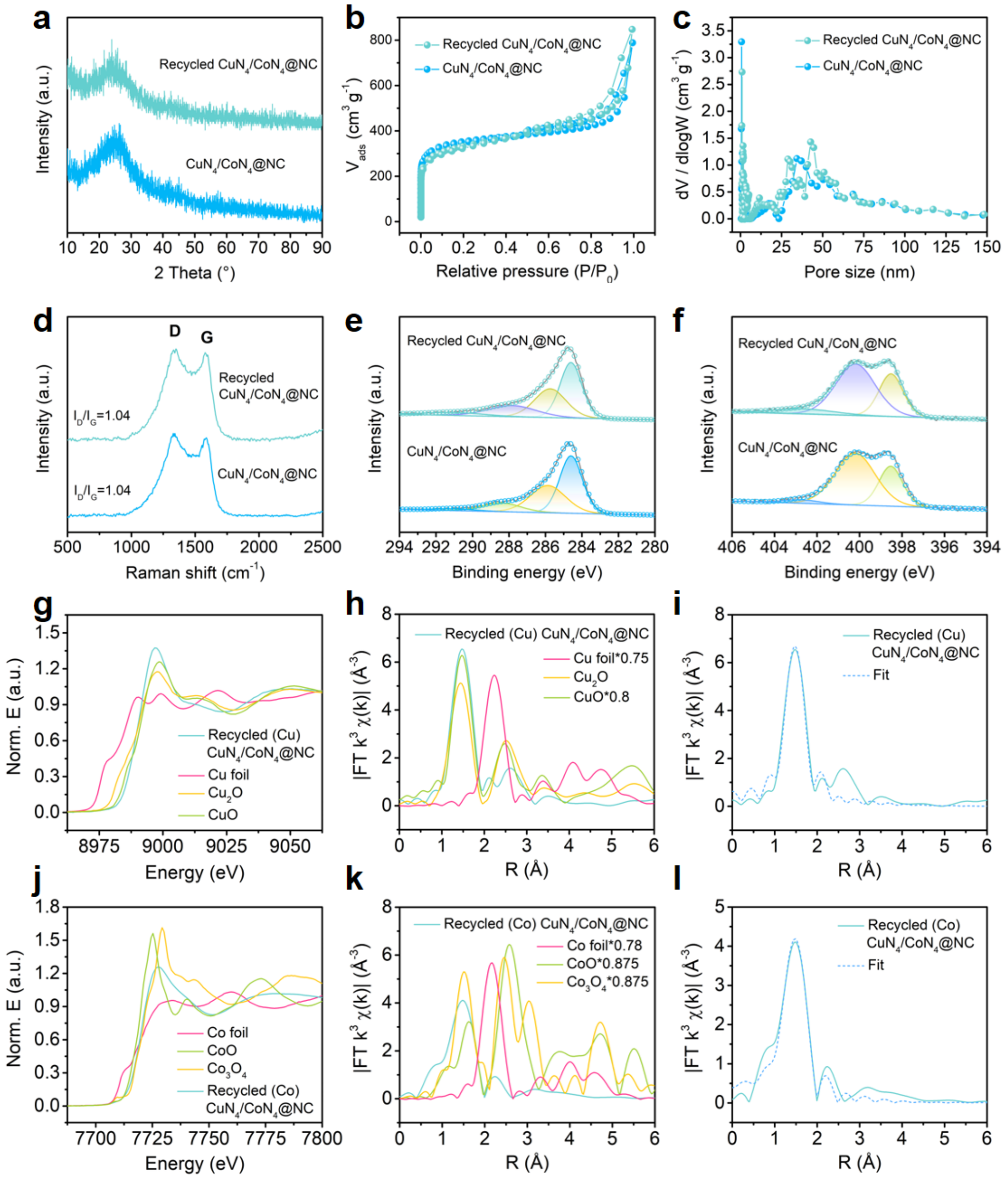

Supplementary Figure 36. Porosity characterizations of the recycled CuN_4/CoN_4@NC. (a) XRD patterns, (b) N_2 adsorption-desorption isotherms, (c) corresponding pore-size distributions, (d) Raman spectra, (e) XPS spectra in C 1s region, and (f) N 1s region of the fresh and recycled CuN_4/CoN_4@NC. (g) Cu K-edge XANES spectra, (h) Fourier-transformed (FT) k^3-weighted EXAFS spectra for the Cu K-edge, (i) Cu K-edge EXAFS fitting curves of recycled CuN_4/CoN_4@NC in R space. (j) Co K-edge XANES spectra, (k) FT k^3-weighted EXAFS spectra for the Co K-edge, (l) Co K-edge EXAFS fitting curves of recycled CuN_4/CoN_4@NC R space.

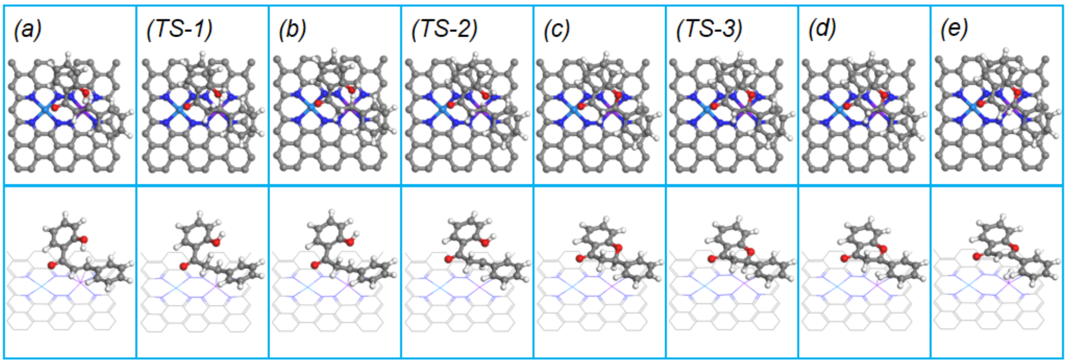

Supplementary Figure 37. Atomistic structures in Figure 4g through different views. TOP and simplified views of the atomistic structures for the reaction pathways from 2'-hydroxychalcone to flavone over CuN_4/CoN_4@NC. The gray, blue, sky blue, purple, red, and white balls represent C, N, Cu, Co, O, and H atoms, respectively. “TS” denotes a transition state.

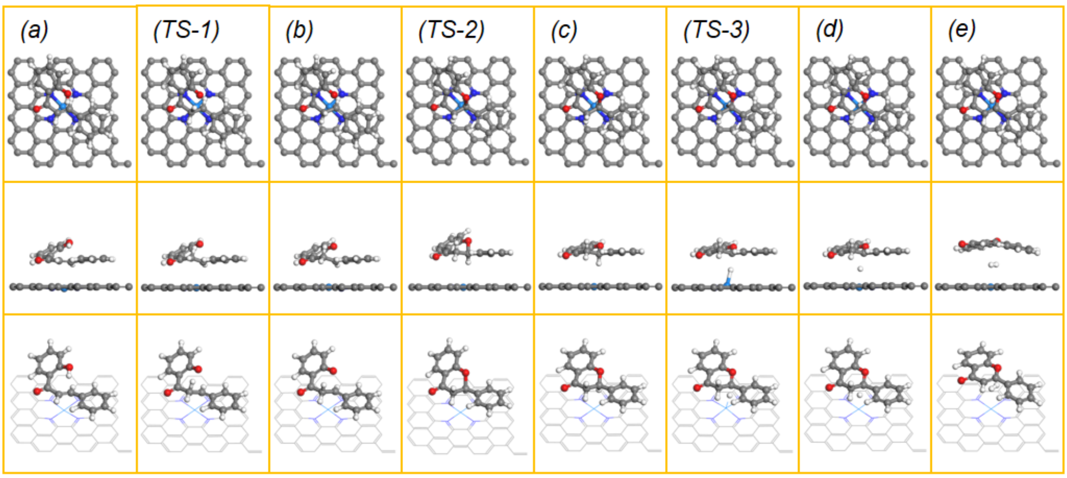

Supplementary Figure 38. Possible atomistic structures during 2'-hydroxychalcone transformation over CuN_4@NC. TOP, side, and simplified views of the atomistic structures for the reaction pathways from 2'-hydroxychalcone to flavone over CuN_4@NC. The gray, blue, sky blue, red, and white balls represent C, N, Cu, O, and H atoms, respectively. "TS" denotes a transition state.

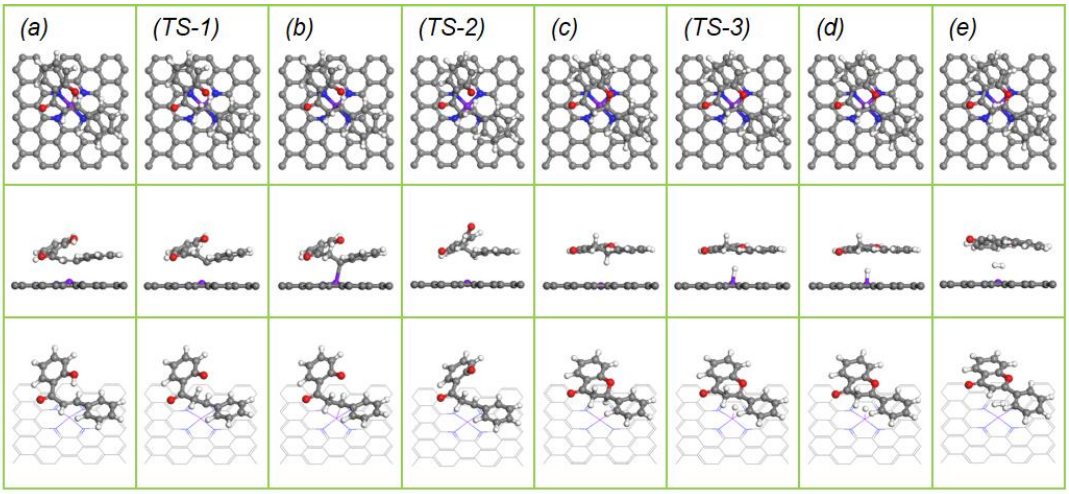

Supplementary Figure 39. Possible atomistic structures during 2'-hydroxychalcone transformation over CoN_4@NC. TOP, side, and simplified views of the atomistic structures for the reaction pathways from 2'-hydroxychalcone to flavone over CoN_4@NC. The gray, blue, purple, red, and white balls represent C, N, Co, O, and H atoms, respectively. “TS” denotes a transition state.

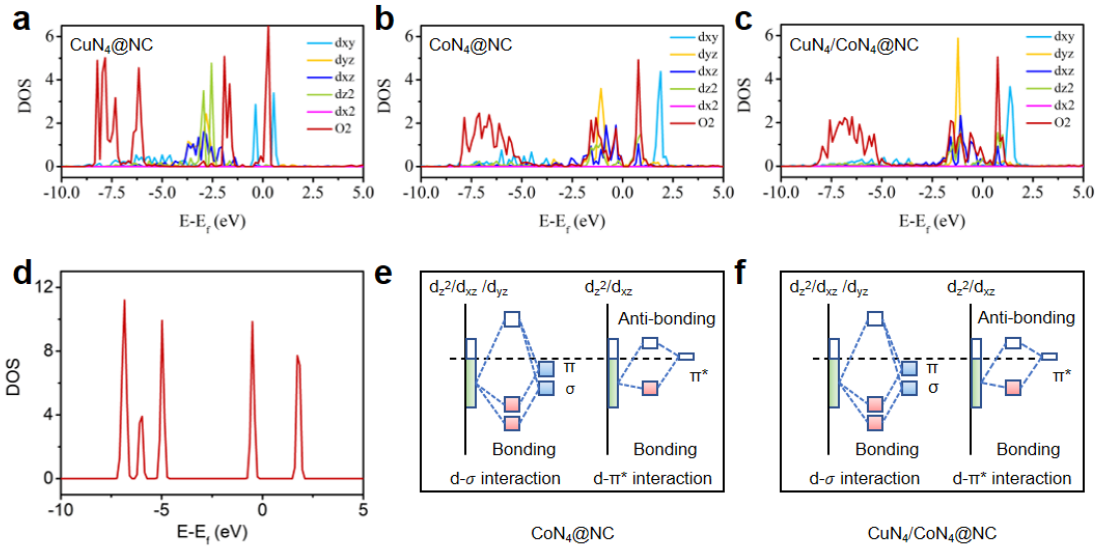

Supplementary Figure 40. Calculated density of states. Density of states of Cu or Co 3d (d_{z^2}, d_{xz}, and d_{yz}) and O_2 (p_z, p_x, and p_y) in (a) CuN_4@NC, (b) CoN_4@NC, and (c) CuN_4/CoN_4@NC. (d) Density of states of free O_2. Orbital interactions between O* and Co site (d_{z^2}–p_z, d_{xz}–p_x, and d_{yz}–p_y) in (e) CoN_4@NC and (f) CuN_4/CoN_4@NC; the schematic illustrations are extracted from corresponding DOS of catalysts.

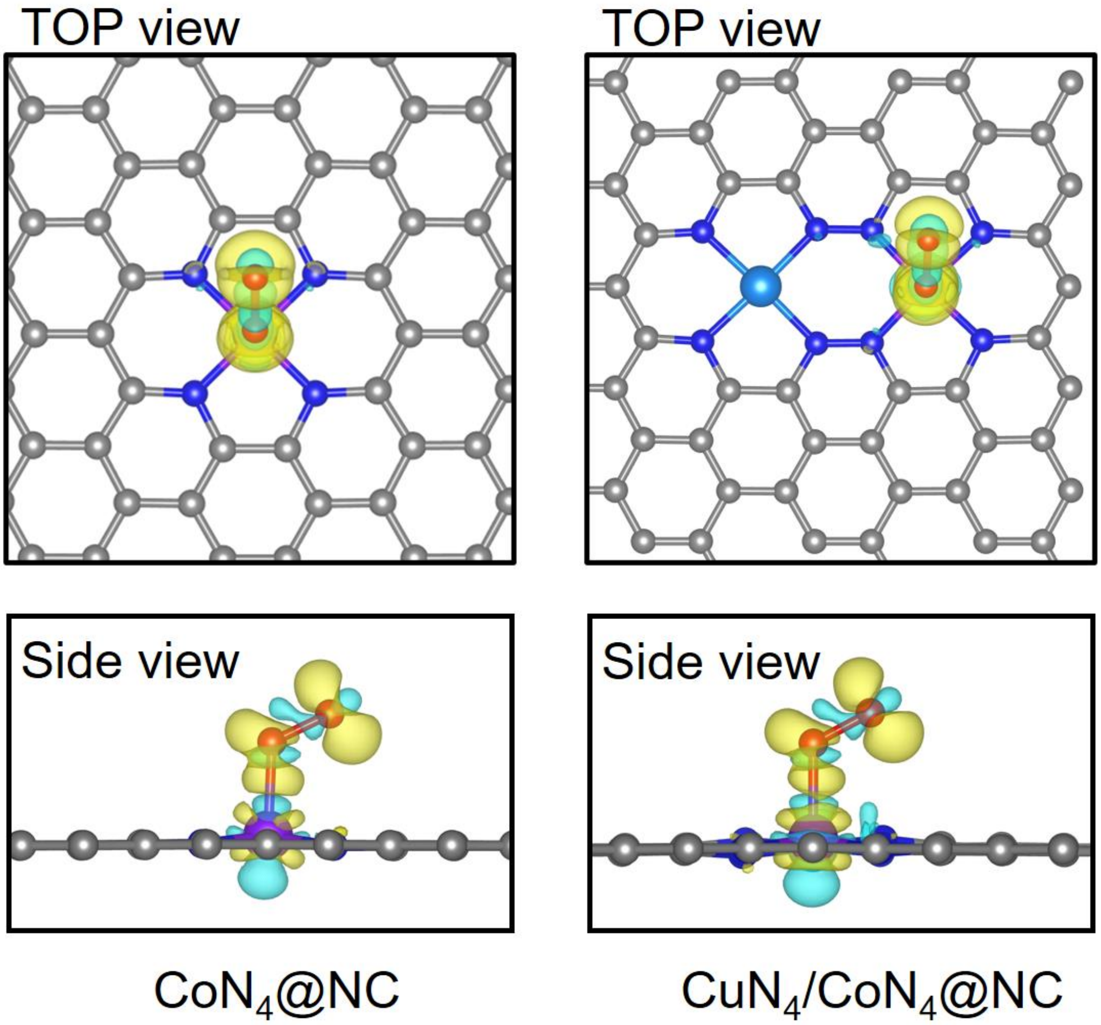

Supplementary Figure 41. Calculated charge difference plots for O_2 adsorption. Top and side view of charge difference plots for O_2 adsorption on CoN_4@NC and CuN_4/CoN_4@NC, yellow and cyan isosurfaces with an isosurface level of 0.003 e/a_0^3 represent electron accumulation and depletion areas, respectively. Cu (sky blue), Co (purple), N (blue), and C (gray).

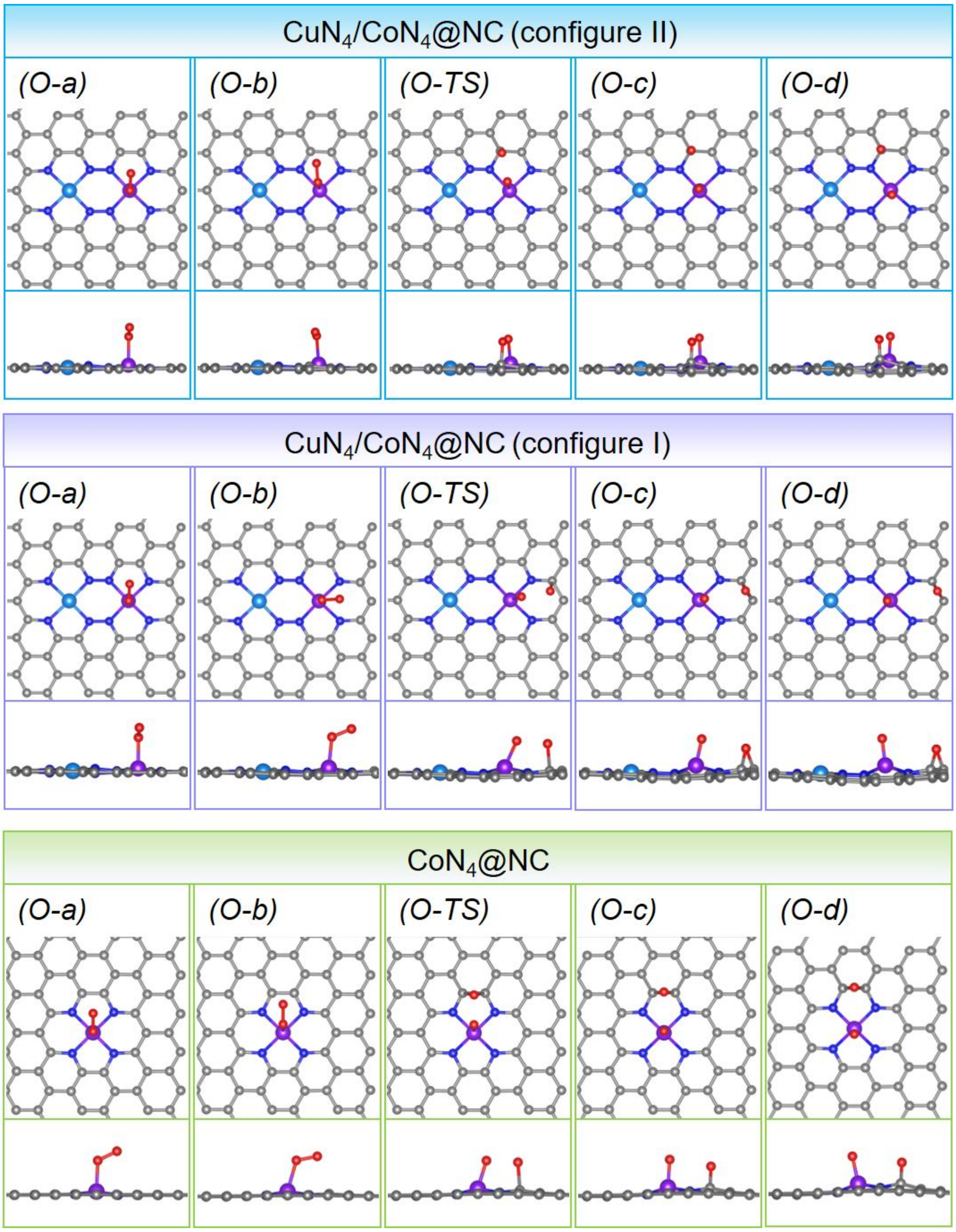

Supplementary Figure 42. Calculated atomistic structures over different samples. Top and side views of the atomistic structures of the initial state, transition state, and final state for the O_2 molecular adsorption, activation, and disassociation. The gray, blue, sky blue, purple, and red balls represent C, N, Cu, Co, and O atoms, respectively. "TS" denotes a transition state.

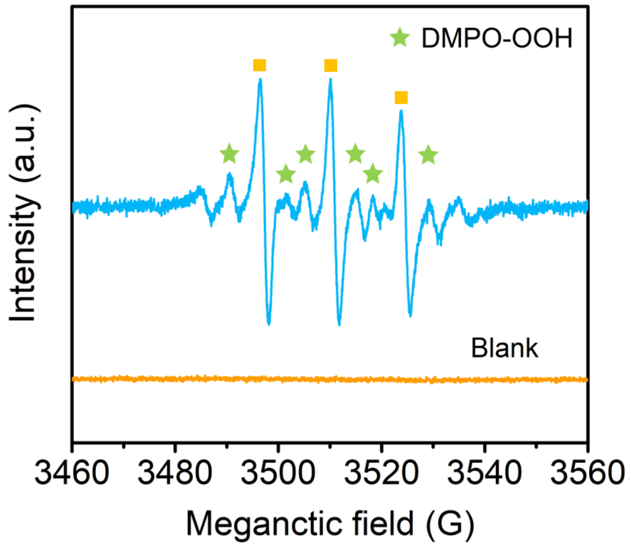

Supplementary Figure 43. EPR spectra of the reaction mixture. EPR spectra of the blank run and optimized reaction route. The strong sextet EPR signals (labeled as asterisks) are assigning to DMPO–OOH, indicating the generation of $\bullet O_2^-$. The three-line DMPOX signals (labeled as blocks) are assigning to the oxidation product of DMPO. No obvious signals are detected in the blank run.

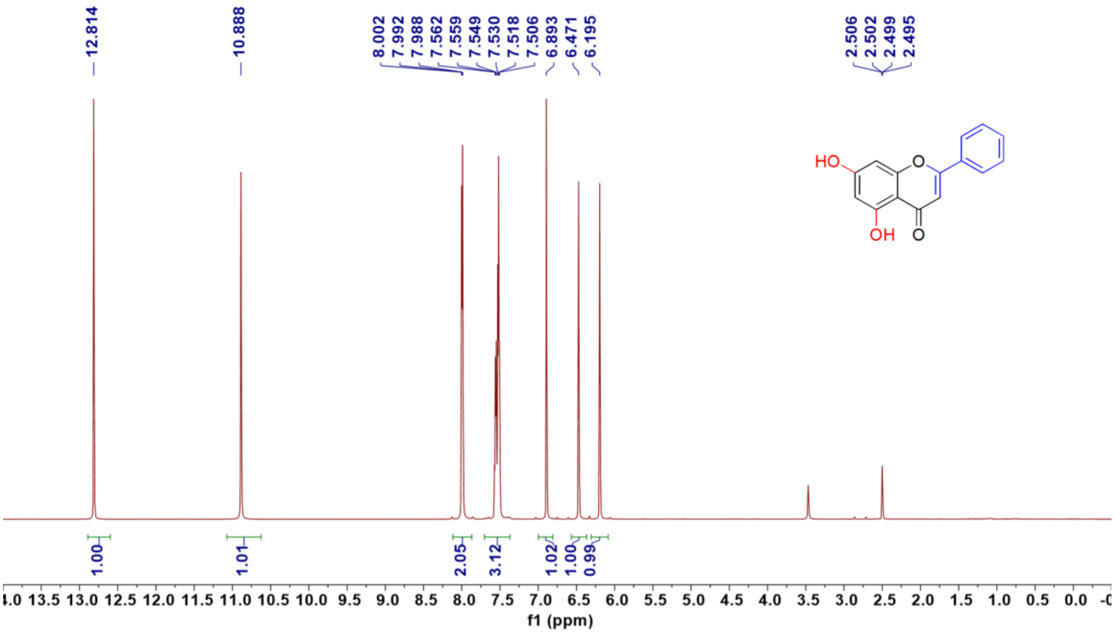

Supplementary Figure 44. ^{1}H-NMR spectrum of the as-synthesized chrysin (29b). ^{1}H NMR (600 MHz, DMSO-d_6) δ 12.81 (s, 1H), 10.89 (s, 1H), 8.12 – 7.87 (m, 2H), 7.70 – 7.37 (m, 3H), 6.89 (s, 1H), 6.47 (s, 1H), 6.20 (s, 1H).

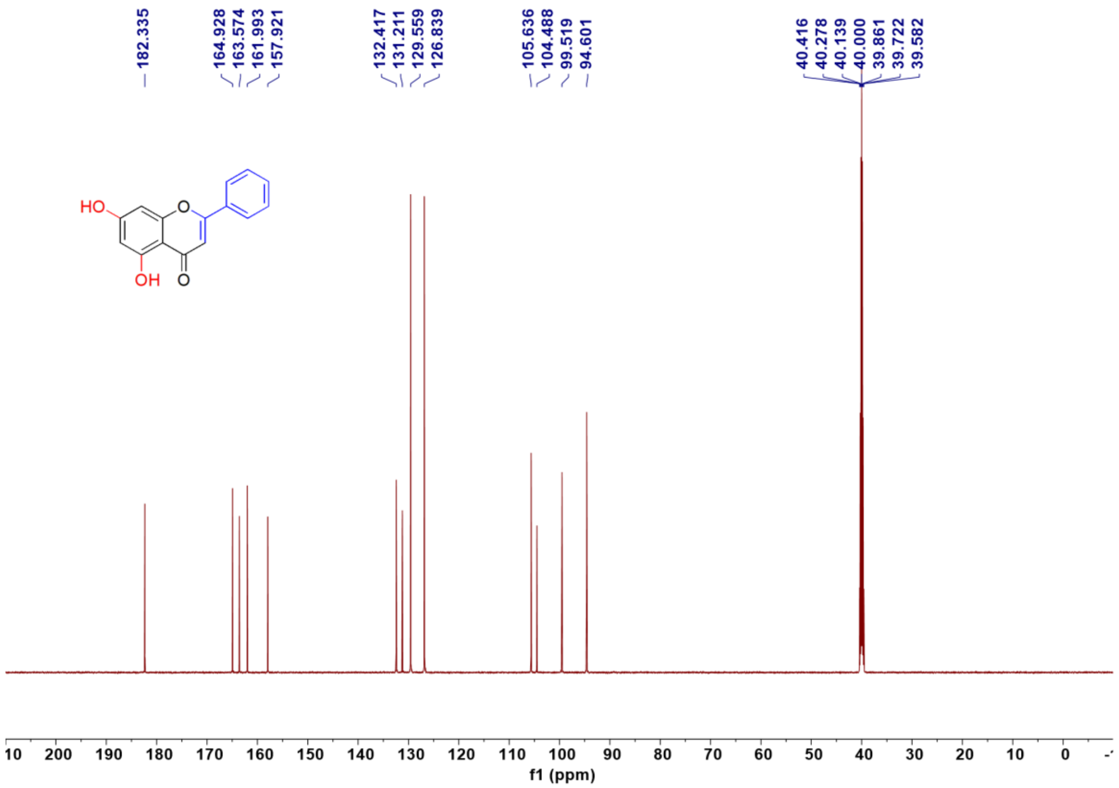

Supplementary Figure 45. ^{13}C-NMR spectrum of the as-synthesized chrysin (29b).

^{13}C NMR (151 MHz, DMSO-d_6) δ 182.34, 164.93, 163.57, 161.99, 157.92, 132.42, 131.21, 129.56, 126.84, 105.64, 104.49, 99.52, 94.60.

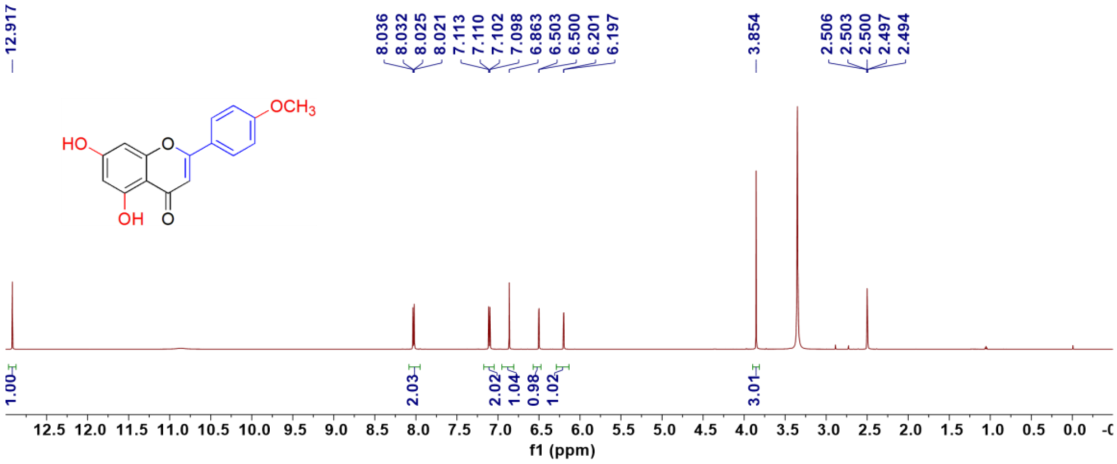

Supplementary Figure 46. ^{1}H-NMR spectrum of the as-synthesized chrysin (30c).

^{1}H NMR (600 MHz, DMSO-*d6*) δ 12.92 (s, 1H), 8.08 – 7.95 (m, 2H), 7.17 – 7.05 (m, 2H), 6.86 (s, 1H), 6.50 (d, J = 2.1 Hz, 1H), 6.20 (d, J = 2.1 Hz, 1H), 3.85 (s, 3H).

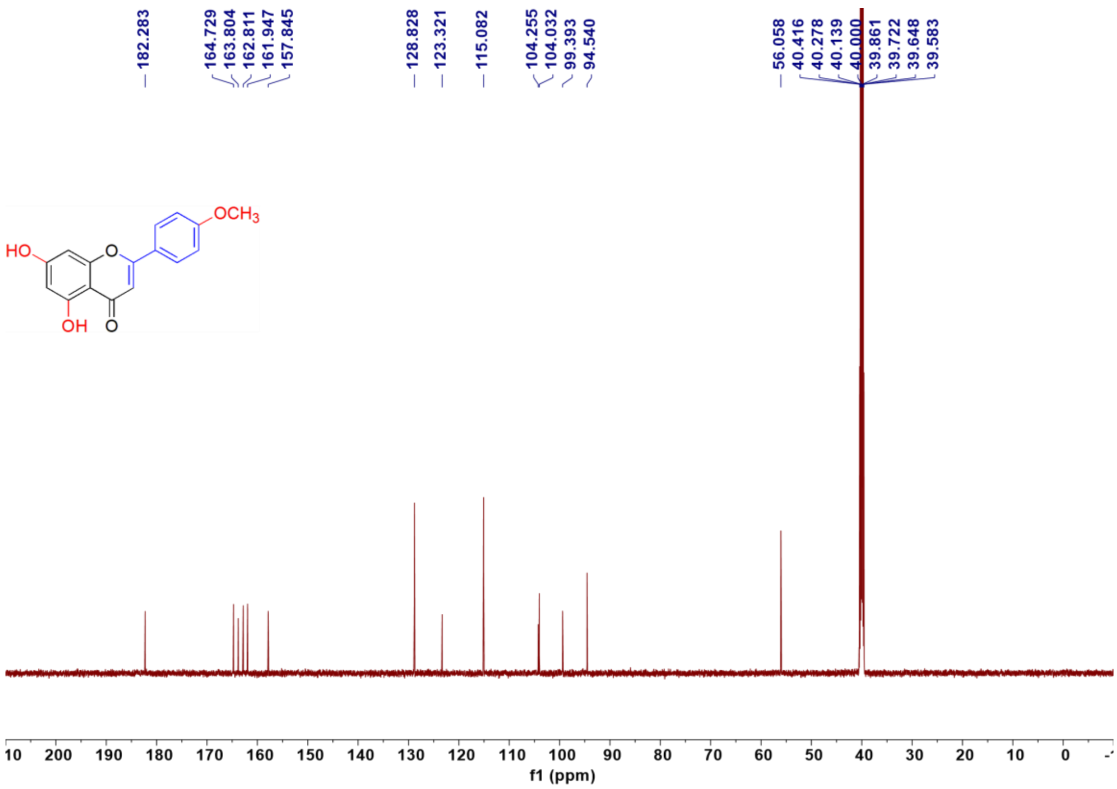

Supplementary Figure 47. ^{13}C-NMR spectrum of the as-synthesized chrysin (30c).

^{13}C NMR (151 MHz, DMSO-d6) δ 182.28, 164.73, 163.80, 162.81, 161.95, 157.85, 128.83, 123.32, 115.08, 104.26, 104.03, 99.39, 94.54, 56.06.

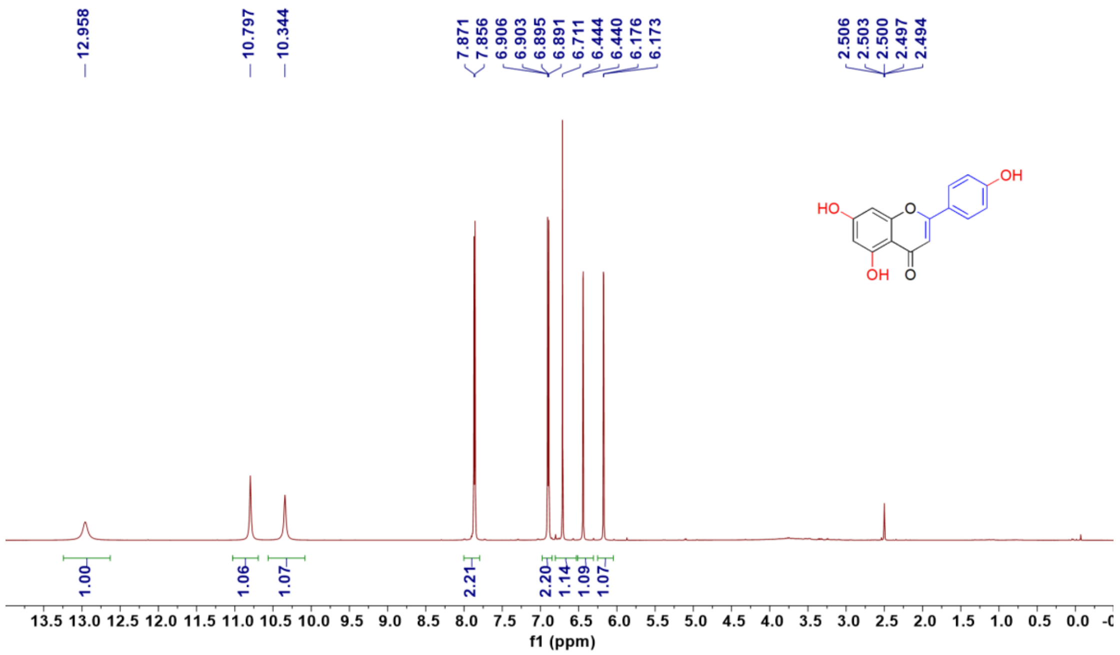

Supplementary Figure 48. ^{1}H-NMR spectrum of the as-synthesized apigenin (31c). ^{1}H NMR (600 MHz, DMSO-d_6) δ 12.96 (s, 1H), 10.80 (s, 1H), 10.34 (s, 1H), 7.86 (d, J = 8.8 Hz, 2H), 6.98 – 6.85 (m, 2H), 6.71 (s, 1H), 6.44 (d, J = 2.1 Hz, 1H), 6.17 (d, J = 2.1 Hz, 1H).

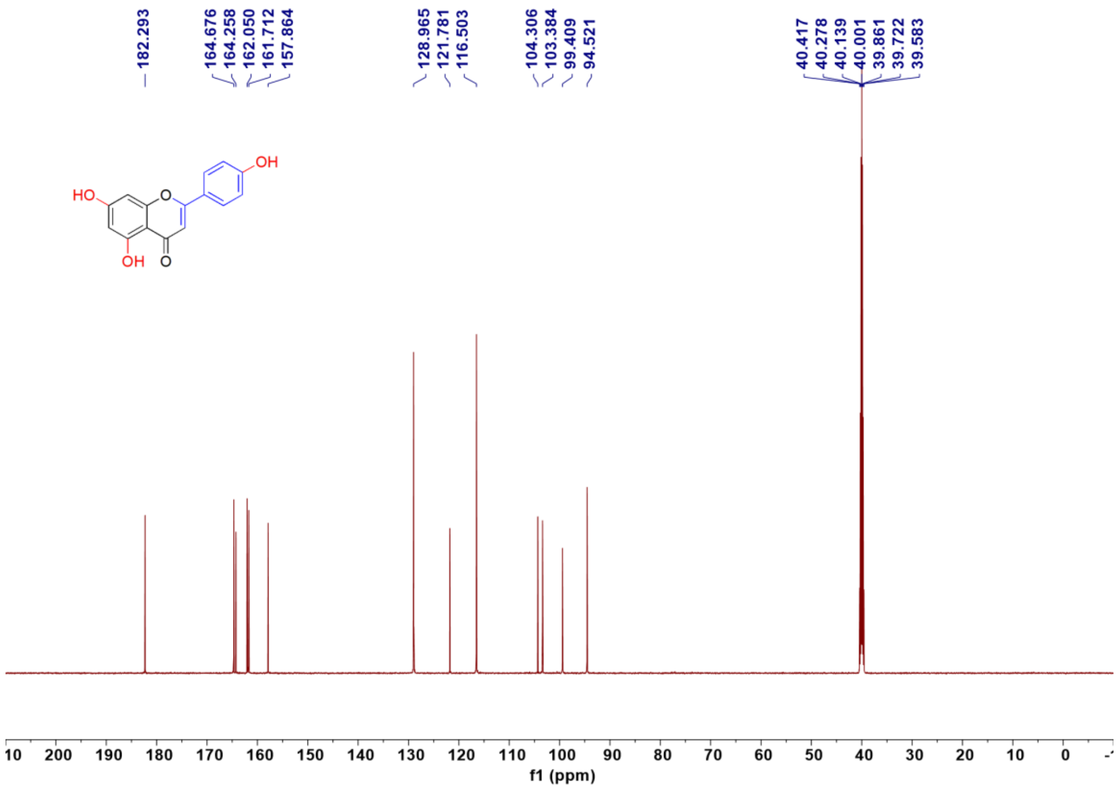

Supplementary Figure 49. ^{13}C-NMR spectrum of the as-synthesized apigenin (31c).

^{13}C NMR (151 MHz, DMSO-d_6) δ 182.29, 164.68, 164.26, 162.05, 161.71, 157.86, 128.97, 121.78, 116.50, 104.31, 103.38, 99.41, 94.52.

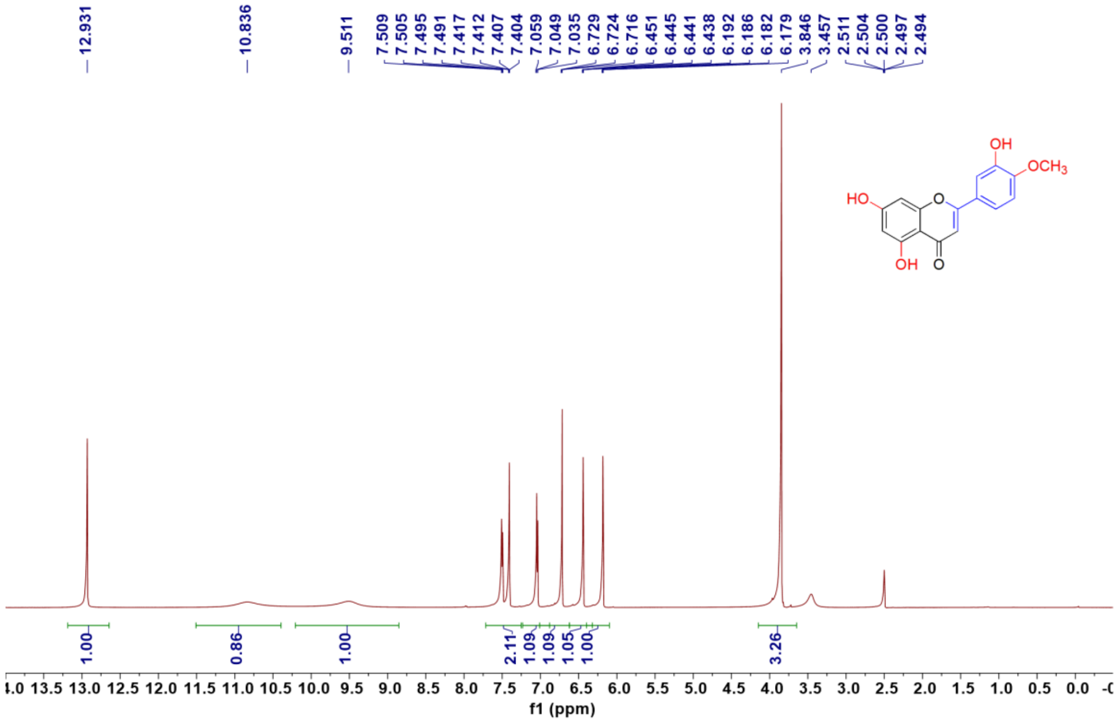

Supplementary Figure 50. ^{1}H-NMR spectrum of the as-synthesized diosmetin (32c).

^{1}H NMR (600 MHz, DMSO-d_6) δ 12.93 (s, 1H), 10.84 (s, 1H), 9.51 (s, 1H), 7.72 – 7.25 (m, 2H), 7.05 (t, J = 7.1 Hz, 1H), 6.72 (d, J = 5.1 Hz, 1H), 6.62 – 6.32 (m, 1H), 6.40 – 6.10 (m, 1H), 3.85 (s, 3H).

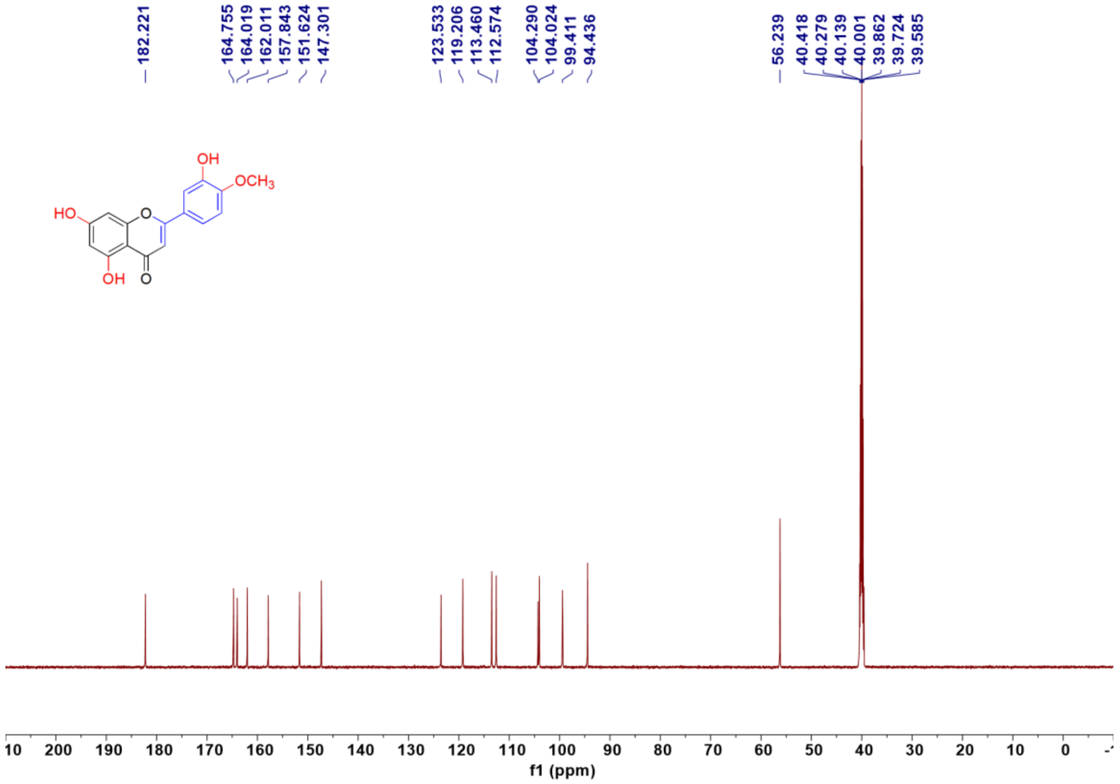

Supplementary Figure 51. ^{13}C-NMR spectrum of the as-synthesized diosmetin (32c).

^{13}C NMR (151 MHz, DMSO-d_6) δ 182.22, 164.75, 164.02, 162.01, 157.84, 151.62, 147.30, 123.53, 119.21, 113.46, 112.57, 104.29, 104.02, 99.41, 94.44, 56.24.

Supplementary Table 1. Preparation conditions of the samples.

Sample	Medium	M_a-based ZIF-8	M_b-based phthalocyanine
CuN_4/CoN_4@NC	KCl-KBr	Cu-ZIF-8	Co-Ph
CuN_4@NC	KCl-KBr	Cu-ZIF-8	–
Co_x@NC	KCl-KBr	–	Co-Ph
CoN_4@NC	KCl-KBr	ZIF-8	Co-Ph
Cu_x/Co_x@NC-750	–	Cu-ZIF-8	Co-Ph
Cu-ZIF-8-750	–	Cu-ZIF-8	–
Co-Ph-750	–	–	Co-Ph
The samples only involved in the reaction as counterparts.			
NC	KCl-KBr	ZIF-8	–
Co-ZIF-8-750	–	Co-ZIF-8	–
The synthesis versatility of the infiltration deposition strategy.			
CuN_4/FeN_4@NC	KCl-KBr	Cu-ZIF-8	Fe-Ph
CoN_4/CuN_4@NC	KCl-KBr	Co-ZIF-8	Cu-Ph
CoN_4/FeN_4@NC	KCl-KBr	Co-ZIF-8	Fe-Ph
NiN_4/CuN_4@NC	KCl-KBr	Ni-ZIF-8	Cu-Ph
NiN_4/CoN_4@NC	KCl-KBr	Ni-ZIF-8	Co-Ph
NiN_4/FeN_4@NC	KCl-KBr	Ni-ZIF-8	Fe-Ph
MnN_4/CuN_4@NC	KCl-KBr	Mn-ZIF-8	Cu-Ph
MnN_4/CoN_4@NC	KCl-KBr	Mn-ZIF-8	Co-Ph
MnN_4/FeN_4@NC	KCl-KBr	Mn-ZIF-8	Fe-Ph

Supplementary Table 2. Specific surface areas, pore volumes, and elemental contents of the as-synthesized materials.

Samples	S_{BET} ($m^2\ g^{-1}$)	$S_{Langmuir}$ ($m^2\ g^{-1}$)	V_{pore} ($cm^3\ g^{-1}$)	Elemental contents (wt%)		
				M_a	M_b	N
CuN_4/CoN_4@NC	1112.2	1913.4	0.87	1.46 (Cu)	1.42 (Co)	13.4
CuN_4@NC	2070.1	4080.7	2.14	1.81	−	11.3
CoN_4@NC	1598.7	1724.6	1.24	2.06	−	7.8
Co_x@NC	245.7	482.9	0.19	8.90	−	6.6
Cu_x/Co_x@NC-750	144.0	217.7	0.08	1.50 (Cu)	1.34 (Co)	7.4
Cu-ZIF-8-750	56.1	104.6	0.04	3.79	−	7.1
Co-Ph-750	26.3	55.4	0.02	4.1	−	6.2
CuN_4/FeN_4@NC	937.1	1778.6	0.70	1.43 (Cu)	1.48 (Fe)	12.1
CoN_4/CuN_4@NC	982.1	1603.4	0.76	1.53 (Co)	1.39 (Cu)	14.0
CoN_4/FeN_4@NC	853.0	1664.4	0.63	1.52 (Co)	1.42 (Fe)	13.3
NiN_4/CuN_4@NC	956.6	1732.3	0.82	1.45 (Ni)	1.49 (Cu)	12.9
NiN_4/CoN_4@NC	972.6	1818.1	0.76	1.44 (Ni)	1.46 (Co)	12.8
NiN_4/FeN_4@NC	886.2	1644.7	0.63	1.42 (Ni)	1.45 (Fe)	11.4
MnN_4/CuN_4@NC	899.8	1821.5	0.82	1.48 (Mn)	1.37 (Cu)	11.8
MnN_4/CoN_4@NC	868.7	1659.0	0.64	1.39 (Mn)	1.53 (Co)	13.4
MnN_4/FeN_4@NC	872.3	1566.3	0.64	1.41 (Mn)	1.44 (Fe)	12.6
Recycled CuN_4/CoN_4@NC	1222.8	1938.5	0.90	1.45 (Cu)	1.44 (Co)	13.8

Supplementary Table 3. Structural parameters of various samples extracted from the EXAFS fitting (S_0^2=0.88).

Sample	Shell	C.N.	R (Å)	$\sigma^2 \times 10^3$ (Å^2)	ΔE_0 (eV)	R factor
Cu foil	Cu–Cu	12*	2.540±0.002	8.59±0.3	6.597±0.38	0.002
Cu_2O	Cu–O	3.2	1.92±0.01	7.2±1.3	0.2±1.3	0.009
	Cu–Cu	11.5	3.02±0.02	32.7±3.6	2.2±1.4	
CuO	Cu–O	4.1	1.95±0.01	5.1±2.1	1.0±2.0	0.019
	Cu–Cu	12.1	2.97±0.04	33.2±8.4	0.0±3.4	
CuN_4@NC	Cu–N	4.2	1.98±0.02	2.91±2.7	3.55±5.3	0.010

C.N.: coordination numbers; *R*: bond distance; σ^2: Debye-Waller factors; ΔE_0: the inner potential correction. *R* factor: goodness of fit. *Fitting with fixed parameter.

Supplementary Table 4. Structural parameters of various samples extracted from the EXAFS fitting (S_0^2=0.77).

Sample	Shell	C.N.	R (Å)	$\sigma^2 \times 10^3$ (Å^2)	ΔE_0 (eV)	R factor
Co foil	Co–Co	12*	2.540±0.002	8.59±0.3	6.597±0.38	0.002
CoO	Co–O	5.7	2.11±0.01	8.5±1.3	0.2±1.7	0.004
	Co–Co	12.5	3.01±0.01	9.7±1.0	−3.2±1.1	
Co_3O_4	Co–O	4.2	1.92±0.01	1.9±0.8	2.3±1.3	0.006
	Co–Co	4.4	2.88±0.02	4.1±2.6	2.5±3.2	
	Co–O	5.0	3.34±0.02	3.2±3.0	−2.8±3.4	
CoN_4@NC	Co–N	4.1	1.89±0.02	4.3±2.2	−4.0±3.2	0.008

C.N.: coordination numbers; *R*: bond distance; σ^2: Debye-Waller factors; ΔE_0: the inner potential correction. *R* factor: goodness of fit. *Fitting with fixed parameter.

Supplementary Table 5. Yields of the CuN_4/CoN_4@NC using the medium-induced infiltration deposition strategy.

Entry	Weight (g)			Yield (%)
	Cu-ZIF-8	Co-Ph	CuN_4/CoN_4@NC	
1	0.1365	0.02	0.0569	41.7
2	0.1358	0.02	0.0574	42.3
3	0.1305	0.02	0.0549	42.1
4	0.1346	0.02	0.0564	41.9
5	0.1329	0.02	0.0555	41.8

Supplementary Table 6. Structural parameters of various samples extracted from the EXAFS fitting (S_0^2=0.84, 0.72).

Sample	Shell	C.N.	R (Å)	$\sigma^2\times10^3$ (Å^2)	ΔE_0 (eV)	R factor
Cu foil	Cu–Cu	12*	2.540±0.002	8.59±0.3	6.597±0.38	0.002
Cu_2O	Cu–O	3.2	1.92±0.01	7.2±1.3	0.2±1.3	0.009
	Cu–Cu	11.5	3.02±0.02	32.7±3.6	2.2±1.4	
CuO	Cu–O	4.1	1.95±0.01	5.1±2.1	1.0±2.0	0.019
	Cu–Cu	12.1	2.97±0.04	33.2±8.4	0.0±3.4	
Cu-ZIF-8	Cu–N	4.0	1.928±0.007	2.7±0.9	6.67±2.05	0.014
CuN_4/CoN_4@NC (for Cu)	Cu–N	3.8	1.966±0.016	3.99±1.0	0.588±2.3	0.016
Co foil	Co–Co	12*	2.490±0.002	6.11±0.3	6.84±0.44	0.002
CoO	Co–O	5.7	2.11±0.01	8.5±1.3	0.2±1.7	0.004
	Co–Co	12.5	3.01±0.01	9.7±1.0	−3.2±1.1	
Co_3O_4	Co–O	4.2	1.92±0.01	1.9±0.8	2.3±1.3	0.006
	Co–Co	4.4	2.88±0.02	4.1±2.6	2.5±3.2	
	Co–O	5.0	3.34±0.02	3.2±3.0	−2.8±3.4	
Co-Ph	Co–N	4.0	1.869±0.031	2.04±2.1	4.379±1.74	0.015
CuN_4/CoN_4@NC (for Co)	Co–N	3.9	1.890±0.019	7.58±1.1	0.809±2.58	0.007

C.N.: coordination numbers; *R*: bond distance; σ^2: Debye-Waller factors; ΔE_0: the inner potential correction. *R* factor: goodness of fit. *Fitting with fixed parameter.

S_0^2 was set as 0.84 for Cu–N in CuN_4/CoN_4@NC, which was obtained from the experimental EXAFS fit of Cu foil reference by fixing C.N. as the known crystallographic value and was fixed to all the samples.

S_0^2 was set as 0.72 for Co–N in CuN_4/CoN_4@NC, which was obtained from the experimental EXAFS fit of Co foil reference by fixing C.N. as the known crystallographic value and was fixed to all the samples.

Supplementary Table 7. Relative energy of the samples in the calculation.

Sample with medium	Energy (eV)	Sample	Energy (eV)
CuN_4/ZnN_4@NC-$K_{16}Cl_4Br_{12}$	−611.14	CuN_4/ZnN_4@NC	−510.75
CuN_4/vacN_4@NC-$K_{16}Cl_4Br_{12}$	−609.39	CuN_4/vacN_4@NC	−508.02
CuN_4/CoN_4@NC-$K_{16}Cl_4Br_{12}$	−616.29	CuN_4/CoN_4@NC	−520.88
vacN_4/ZnN_4@NC-$K_{16}Cl_4Br_{12}$	−607.29	vacN_4/ZnN_4@NC	−506.08
CuN_4@NC-$K_8Cl_2Br_6$	−582.49	CuN_4@NC	−535.23
ZnN_4@NC-$K_8Cl_2Br_6$	−580.67	ZnN_4@NC	−533.24
vacN_4@NC-$K_8Cl_2Br_6$	−576.77	vacN_4@NC	−529.76
$K_8C_{12}Br_6$	−45.67	–	–
Cu-$K_8Cl_2Br_6$	−49.40	Cu atom	−0.24
Zn-$K_8Cl_2Br_6$	−47.96	Zn atom	−0.011
Co-$K_8Cl_2Br_6$	−50.98	–	–
ZnN_4-$K_8Cl_2Br_6$	−72.49	–	–
defect@NC-$K_8Cl_2Br_6$	−530.58	–	–
CuN_4-defect@NC-$K_8Cl_2Br_6$	−563.33	–	–

Supplementary Table 8. Bader charge of the CuN_4@NC, CoN_4@NC, and CuN_4/CoN_4@NC models.

Sample	Atom	Bader charge (*e*)
CuN_4@NC	Cu	0.923
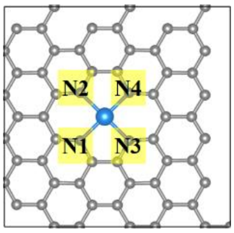	N1	−1.221
	N2	−1.216
	N3	−1.158
	N4	−1.157
CoN_4@NC	Co	0.863
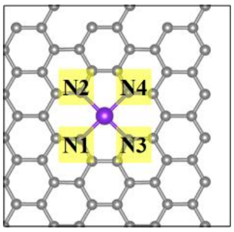	N1	−1.221
	N2	−1.221
	N3	−1.148
	N4	−1.146
	Cu	0.939
	Co	0.849
CuN_4/CoN4@NC	N1	−1.204
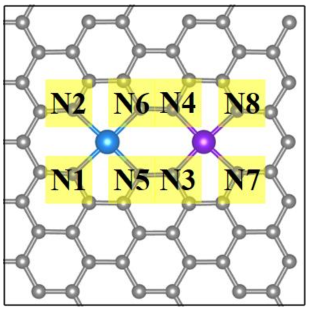	N2	−1.170
	N3	−0.492
	N4	−0.543
	N5	−0.796
	N6	−0.784
	N7	−1.166
	N8	−1.184

Supplementary Table 9. Results of the one-pot cascade reaction to flavone over different catalysts. [a]

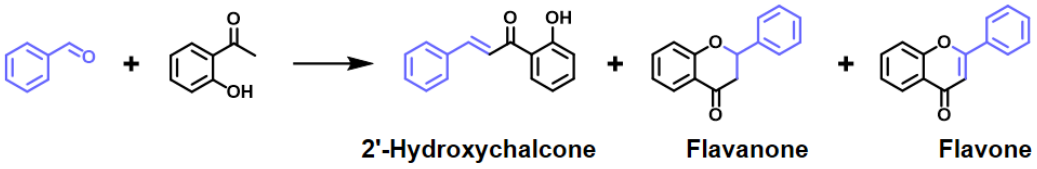

Entry	Catalyst	Conv. (%)	Yield (%)		
			2'-Hydroxychalcone	Flavanone	Flavone
1	–	5.7	5.7	–	–
2	NC	10.2	8	2.2	–
3	CuN_4/CoN_4@NC	99	–	–	99
4	CuN_4@NC	52	7	31	14
5	CoN_4@NC	71	4	24	43
6	Co_x@NC	48	6	15	27
7	Cu_x/Co_x@NC-750	55	4	19	32
8	CuN_4@NC+CoN_4@NC	79	5	23	51
9	Cu-ZIF-8-750	17	4	6	7
10	Co-Ph-750	26	6	9	11
11	600-Cu/Co@NC	48	26	14	8
12	900-Cu/Co@NC	81	20	15	46
13[b]	CuN_4/CoN_4@NC	94	8	79	7

[a] Reaction conditions: benzaldehyde (1 mmol), 2'-hydroxyacetophenone (1.5 mmol), catalyst (total metal, 1.4 mol% relative to benzaldehyde), *n*-hexanol (4 mL), O_2 (2 bar), 140 °C, 12 h. Conversion and yield were determined by GC–MS based on benzaldehyde.

[b] N_2 (1 bar).

Supplementary Table 10. Catalytic results of oxidative dehydrogenation of flavanone to flavone over different catalysts.

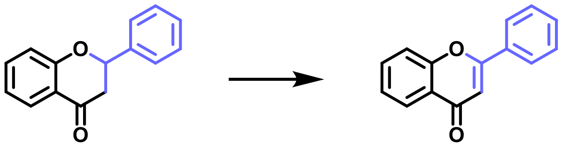

Entry	Catalyst	Conv. (%)	Yield of flavone (%)
1	CuN_4/CoN_4@NC	99	99
2	CuN_4@NC	20	20
3	CoN_4@NC	49	49
4	Co_x@NC	33	33
5	CuN_4@NC+CoN_4@NC	60	60
6	Cu_x/Co_x@NC-750	43	43

Reaction conditions: flavanone (1 mmol), catalyst (total metal, 1.4 mol% relative to flavanone), *n*-hexanol (4 mL), O_2 (2 bar), 140 °C, 12 h. Conversion and yield were determined by GC–MS.

Supplementary Table 11. Structural parameters of various samples extracted from the EXAFS fitting (S_0^2=0.87, 0.76).

Sample	Shell	C.N.	R (Å)	$\sigma^2\times10^3$ (Å^2)	ΔE_0 (eV)	R factor
Cu foil	Cu–Cu	12*	2.54±0.01	8.3±0.3	3.8±0.5	0.002
Cu_2O	Cu–O	3.2±0.4	1.92±0.01	7.2±1.3	0.2±1.3	0.009
	Cu–Cu	11.5±3.2	3.02±0.02	32.7±3.6	2.2±1.4	
CuO	Cu–O	4.1±0.8	1.95±0.01	5.1±2.1	1.0±2.0	0.019
	Cu–Cu	12.1±2.6	2.97±0.04	33.2±8.4	0.0±3.4	
Recycled (for Cu) CuN_4/CoN_4@NC	Cu–N	4.5±0.4	1.98±0.01	4.7±1.5	−1.2±1.8	0.013
Co foil	Co–Co	12*	2.50±0.01	6.2±0.2	6.7±0.3	0.001
CoO	Co–O	5.7±0.9	2.11±0.01	8.5±1.3	0.2±1.7	0.004
	Co–Co	12.5±1.5	3.01±0.01	9.7±1.0	−3.2±1.1	
Co_3O_4	Co–O	4.2±0.4	1.92±0.01	1.9±0.8	2.3±1.3	0.006
	Co–Co	4.4±1.8	2.88±0.02	4.1±2.6	2.5±3.2	
	Co–O	5.0±2.6	3.34±0.02	3.2±3.0	−2.8±3.4	
Recycled (for Co) CuN_4/CoN_4@NC	Co–N	4.8±0.9	1.94±0.01	12.7±1.6	0.3±1.1	0.010

C.N.: coordination numbers; *R*: bond distance; σ^2: Debye-Waller factors; ΔE_0: the inner potential correction. *R* factor: goodness of fit. *Fitting with fixed parameter.

Supplementary Table 12. The adsorption and disassociation energies of O_2 on different samples and the related O–O bond lengths.

Sample	O_2 adsorption energy (eV)	O–O bond length (Å)	Activation energy (eV)	O_2 disassociation energy (eV)
CuN_4@NC	−0.22	1.25	–	–
CoN_4@NC	−0.88	1.29	2.96	1.53
CuN_4/CoN_4@NC (Configuration-I)	−0.82	1.29	3.21	0.85
CuN_4/CoN_4@NC (Configuration-II)	−0.82	1.29	2.36	1.05
