# Supplementary material for: Atomic design of dual-metal hetero-single-atoms for high-efficiency synthesis of natural flavones
Source: Nat Commun. 2022 Dec 22;13:7873. doi: 10.1038/s41467-022-35598-3 (PMC9780242; doi:10.1038/s41467-022-35598-3)
Supplement: Supplementary file 2 — Description of Additional Supplementary Files [file 41467_2022_35598_MOESM2_ESM.pdf]

Description of Additional Supplementary Files

File Name: Supplementary Movie 1
Description: **3D reconstruction of a CuN_4/CoN_4@NC particle,** the perspective view of inner structure.

File Name: Supplementary Movie 2
Description: **3D reconstruction of a CuN_4/CoN_4@NC particle,** the sectional view of different thickness.

File Name: Supplementary Movie 3
Description: The pyrolysis process recorded by *in-situ* TEM.
